# Causal analysis of 731 immunophenotypes and heart failure: A bidirectional Mendelian randomization study

**DOI:** 10.1097/MD.0000000000042530

**Published:** 2025-05-23

**Authors:** Zhenyu Yang, Jixin Li, Fengzhao Liu, Xiaohan Xiu, Weibo Zhong, Zhigang Sun, Xinyu Zhu, Mengzhu Chen, Xihao Chen, Haohong Zheng, Dandan Guo

**Affiliations:** a The Second Clinical Medical College of Heilongjiang University of Chinese Medicine, Harbin, China; b Department of Acupuncture, Xiyuan Hospital, China Academy of Chinese Medical Sciences, Beijing, China; c Department of Cardiovascular Medicine, Xiyuan Hospital, China Academy of Chinese Medical Sciences, Beijing, China; d Department of Critical Care Medicine, The First Affiliated Hospital of Chongqing Medical University, Chongqing, China; e Department of Cardiovascular 1, The Second Affiliated Hospital of Heilongjiang University of Chinese Medicine, Harbin, China.

**Keywords:** heart failure, immunophenotypes, Mendelian randomization

## Abstract

The aim of this study was to elucidate the causal relationship between immunophenotypes and heart failure (HF) using bidirectional Mendelian randomization (MR) analysis. Summary-level data for HF and immunophenotypes were obtained from public genome-wide association study data. Five robust MR methods were employed to delineate the causal effects between the 2. Further analyses included horizontal pleiotropic analysis, Cochran *Q* analysis, MR-Egger intercept test, and leave-one-out analysis. Finally, we used the screened immunophenotypes as outcomes and HF as exposure for reverse MR analyses. Eight immunophenotypes demonstrated an increased risk of HF, including immunoglobin D^+^ (IgD^+^) CD38br absolute cell (AC); double positive (CD4^+^CD8^+^) %leukocyte; CD28^−^ CD127^−^ CD25^++^ CD8br %T cell; CD28^−^ CD127^−^ CD25^++^ CD8br %CD8br; CD28^+^ CD45RA^+^ CD8br %T cell; CD19 on IgD^+^ CD38br; CD27 on IgD^−^ CD38^dim^; CD45 on lymphocyte. Conversely, 7 immunophenotypes exhibited a reduced risk of HF, including Activated Treg AC; Im myeloid-derived suppressor cell %CD33^dim^ human leukocyte antigen DR^−^ (HLA DR^−^) CD66b^−^; CD33^dim^ HLA DR^+^ CD11b^+^ %CD33^dim^ HLA DR^+^; CD20 on IgD^−^ CD38^dim^; side scatter-A (SSC-A) on CD14^+^ monocyte; SSC-A on HLA DR^+^ natural killer cell; CD11b on CD14^+^ monocyte. Importantly, we did not find any horizontal multidimensional outliers, genetic heterogeneity, directional pleiotropy, or a single nucleotide polymorphism that determines ultimate causality. The results of the reverse MR analysis were not statistically significant. In this study, the genetic correlation between 15 immunophenotypes and HF was revealed by MR analysis, which provides a reference for future clinical treatment.

## 1. Introduction

Heart failure (HF) represents a clinical syndrome characterized by insufficient cardiac output due to abnormal myocardial diastolic or systolic function, stemming from various causative factors. This condition leads to circulatory dysfunction in patients.^[1,2]^ Regarded as the prominent cardiovascular ailment of the 21st century, HF stands as the ultimate stage of diverse heart diseases. Individuals affected by HF commonly exhibit inflammatory and metabolic disorders, such as diabetes mellitus, atrial fibrillation, and chronic kidney disease, resulting in an unfavorable clinical prognosis.^[3]^ Owing to its high prevalence, complications, and mortality rates, HF imposes a considerable economic burden on society.^[4]^ A 2019 meta-analysis^[5]^ that included 1.5 million patients with HF showed that the combined 1-, 2-, 5-, and 10-year survival rates for patients with HF were 87%, 73%, 57%, and 35%, respectively. Epidemiological studies^[6]^ estimate approximately 64.3 million individuals affected by HF worldwide, with about 3 million new cases annually. Given the escalating global population and aging demographics, the worldwide prevalence of HF is anticipated to surge further.

With the increasing research on the pathomechanisms of HF, there is growing evidence that immunophenotypes are involved in the development of HF.^[7–10]^ Nonetheless, existing findings regarding the association between immune inflammation and HF have displayed inconsistencies, potentially attributed to limited sample sizes, study design flaws, and confounding factors beyond the current scope of research. Notably, investigations have presented varied perspectives: 1 study demonstrated the potential of the immune system to repair damaged cardiac tissue and suggested the promise of immune cells as a new therapeutic intervention,^[7]^ while another study emphasized the detrimental effects of immune cell activation and myocardial infiltration on the pathogenesis of HF.^[8]^ Additionally, a cohort study showcased a lower HF risk associated with a higher proportion of CD4^+^ T helper (Th) 1 cells.^[10]^ Despite extensive research, primarily observational in nature, the susceptibility of these results to reverse causality hampers the conclusive inference of a causal link between immunophenotypes and HF. Establishing causality through randomized controlled trials demands significant human and financial resources, time, and ethical considerations, posing challenges in general population studies. Alternatively, Mendelian randomization (MR) emerges as a viable proxy for exploring the causal relationship between immunophenotypes and HF.

MR is a robust method in epidemiologic and genetic studies that utilizes genetic variation as instrumental variables (IVs) to explore causal relationships between exposures and outcomes.^[11]^ Based on Mendel second law, the random assignment of genetic variation from parents to offspring during gametogenesis safeguards the genotype-phenotype link from biases introduced by confounding factors and reverse causation often observed in observational studies.^[12]^ Encouraged by these insights, this study undertook the first comprehensive MR analysis to elucidate the potential causal connections between immunophenotypes and HF.

## 2. Methods

### 2.1. Research design

**Figure 1.**
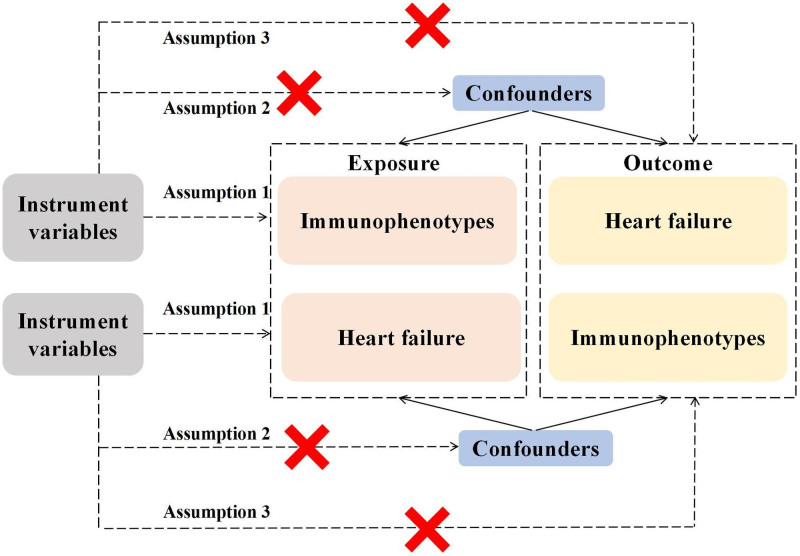
The flow chart of this study.

In this study, 731 immunophenotypes were used as exposures, and HF was used as the outcome to screen appropriate single nucleotide polymorphisms (SNPs) as IVs for MR analysis. Subsequent analyses included horizontal pleiotropic analysis, Cochran *Q* analysis, MR-Egger intercept test, and leave-one-out analysis to confirm the reliability of causality.^[13]^ In addition, we used all screened positive exposures as endpoints for reverse MR analysis to determine reverse causality. This MR study rested upon 3 core assumptions: association hypothesis: IVs closely associated with exposure factors; independence hypothesis: IVs should be independent of any confounding factors related to exposure and outcome; and exclusivity hypothesis: IVs do not directly influence outcomes but affect them solely through exposure factors. The study adopted a 2-sample MR design to explore the causal correlation between immune cells and HF (Fig. 1).

### 2.2. Data source

Genome-wide association study (GWAS) aggregate statistics for each immunophenotype are publicly available in the GWAS catalog (entry numbers from GCST90001391 to GCST90002121).^[14]^ Based on a sample of 3757 Sardinian samples (43% male, 57% female), this GWAS analysis adjusted for sex, age, and age^2^ followed by approximately 22 million SNPs genotyped by high-density array.^[15]^ In this study, 122 important independent association signals were detected at 70 sites, and the molecules and mechanisms involved in the regulation of cellular characteristics were identified. The study analyzed absolute cell (AC) counts (n = 118), median fluorescence intensities (MFI) reflecting surface antigen levels (n = 389), morphological parameters (MP, forward scatter [FSC] and side scatter [SSC], which are proportional to the cell volume, and intracellular cell volume. cell volume, and intracellular complexity and the surface texture of cells, respectively) (n = 32), relative cell (RC) counts (n = 192) by flow cytometry. The pooled data for HF is the largest GWAS currently available containing 977,323 individuals of European ancestry (*n*_case_ = 47,309, *n*_control_ = 930,014, *n*_SNPs_ = 7773,021), which was obtained from the HERMES Consortium (GWAS ID: ebi-a-GCST009541, https://gwas.mrcieu.ac.uk/datasets/ebi-a-GCST009541/). All of the above data have been widely used in previous MR studies and have high reliability.^[16–20]^

### 2.3. Screening of instrumental variables

Based on the GWAS database described above, quality control criteria were developed through the TwoSampleMR software package in R.4.3.1 to screen SNPs for MR analysis. First, according to a recent study,^[21]^ the threshold, kilobase pair (kb), and parameter *r*^2^ as exposed SNPs were set to loose thresholds of *P* < 5 × 10^–6^, 10,000, and 0.001, respectively. In addition, for HF, the significance threshold is set at 5 × 10^–8^. Second, to ensure that there was a strong correlation between SNPs and exposure, we also calculated *F* statistics and excluded IVs with low *F* statistics (<10), as they were considered weak IVs.^[22,23]^ The *F* statistic is calculated as *F* = β^2^/SE^2^, where SE is the standard error and β is the effect size.^[24,25]^ Finally, SNPs with palindromic structures should be excluded when harmonizing exposures and results using the harmonise_data function.^[25]^ To ensure that each SNP is not directly related to the outcome but strongly related to the exposure, SNPs with *P*_outcome_ > *P*_exposure_ should be eliminated after harmonizing exposure and outcome.

### 2.4. Statistical analysis

Various robust analytical MR methods were employed, including weighted median (WM), inverse variance weighted (IVW), MR-Egger, weighted mode, and simple mode. IVW can calculate the Wald ratio for each SNP to estimate the effect of exposure on the outcome and is the predominant analytical method.^[26]^ MR-Egger, on the other hand, estimates causal effects by means of slope coefficients.^[24]^ Compared to IVW, WM has smaller Type I errors and is complementary to MR-Egger. simple mode and weighted mode have lower test efficacy than the previous 3 methods,^[27]^ but have also been used as estimates of causal effects. Sensitivity analyses encompassed MR Pleiotropy RESidual Sum and Outlier (MR-PRESSO) global tests, Cochran *Q* analysis, MR-Egger intercept, and leave-one-out analyses. MR-PRESSO global test is used to identify horizontal multidimensional outliers^[28]^; Cochran *Q* analysis is used to perform heterogeneity analysis; MR-Egger intercept is used to test for directional pleiotropy of genetic variation; and, finally, leave-one-out allows for testing the stability of the results by removing every SNP in the exposure.

### 2.5. Visual analysis

For the main results of MR analysis, we plotted scatter plots, in which each black dot corresponds to a different SNP, and different slashes represent different analysis methods. Odds ratios (OR) > 1 indicated positive correlations, whereas OR < 1 indicated negative correlations. Sensitivity analyses were represented by leave-one-out sensitivity analysis plots.^[23]^ In addition, the funnel plots show whether the selected IVs are biased or not, and the forest plots show the effect of each SNP on the results.

## 3. Results

### 3.1. Overview

Setting the threshold criteria at *P* < 5 × 10^−6^ for exposure, kb at 10,000, and parameter *r*^2^ at 0.001, we identified 15 immune cells potentially causally linked with HF (Table 1).

**Table 1:** 15 immune cells.

Trait type	Panel	Name	ID
AC	B cell	IgD^+^ CD38br AC	ebi-a-GCST90001392
AC	Regulatory cell	Activated Treg AC	ebi-a-GCST90001486
RC	Myeloid cell	Im MDSC %CD33^dim^ HLA DR^−^ CD66b^−^	ebi-a-GCST90001516
RC	Myeloid cell	CD33^dim^ HLA DR^+^ CD11b^+^ %CD33^dim^ HLA DR^+^	ebi-a-GCST90001526
RC	TBNK	DP (CD4^+^CD8^+^) %leukocyte	ebi-a-GCST90001608
RC	Regulatory cell	CD28^−^ CD127^−^ CD25^++^ CD8br %T cell	ebi-a-GCST90001673
RC	Regulatory cell	CD28^−^ CD127^−^ CD25^++^ CD8br %CD8br	ebi-a-GCST90001674
RC	Regulatory cell	CD28^+^ CD45RA^+^ CD8br %T cell	ebi-a-GCST90001688
MFI	B cell	CD19 on IgD^+^ CD38br	ebi-a-GCST90001729
MFI	B cell	CD20 on IgD^−^ CD38^dim^	ebi-a-GCST90001757
MFI	B cell	CD27 on IgD^−^ CD38^dim^	ebi-a-GCST90001804
MFI	Myeloid cell	CD45 on lymphocyte	ebi-a-GCST90002041
MP	TBNK	SSC-A on CD14^+^ monocyte	ebi-a-GCST90002074
MP	TBNK	SSC-A on HLA DR^+^ NK	ebi-a-GCST90002077
MFI	Myeloid cell	CD11b on CD14^+^ monocyte	ebi-a-GCST90002091

AC = absolute cell, DP = double positive, HLA DR = human leukocyte antigen DR, IgD = immunoglobulin D, MDSC = myeloid-derived suppressor cell, MFI = median fluorescence intensity, MP = morphological parameter, NK = natural killer cell, RC = relative cell, SSC-A = side scatter-A, TBNK = T cells, B cells and NK cells.

### 3.2. Instrumental variables

In this study, sufficient IVs were selected for MR analysis, and the *F* statistics of all SNPs was >10, which indicated that there were no weak IVs. At the same time, we excluded a small number of palindromic SNPs and ensured that the *P*_outcome_ > *P*_exposure_ of the remaining SNPs, and the final IVs included in MR analysis are shown in Tables S1 to S15, Supplemental Digital Content, https://links.lww.com/MD/O979. Screening all SNPs for repeat values, we found that rs1801274 corresponded to 3 immune cells [CD33^dim^ human leukocyte antigen DR (HLA DR^+^) CD11b^+^ %CD33^dim^ HLA DR^+^, CD11b on CD14^+^ monocyte and CD27 on immunoglobin D^−^ (IgD^−^) CD38^dim^], and rs115642223 and rs111856210 corresponded to 2 immune cells (CD28^−^ CD127^−^ CD25^++^ CD8br %T cell and CD28^−^ CD127^−^ CD25^++^ CD8br %CD8br).

### 3.3. Impact of immune cells on HF

#### 3.3.1. *Effect of IgD*^*+*^ *CD38br AC on HF*

**Figure 2.**
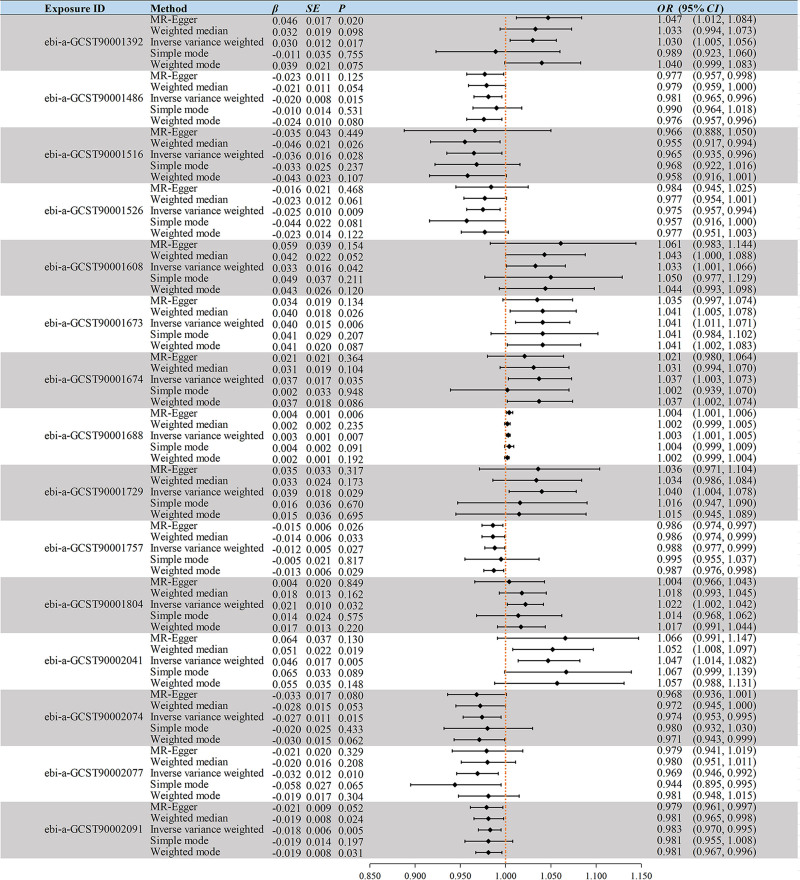
Mendelian randomization’s 5 types of analytical results. β = effect size, CI = confidence interval, OR = odds ratio, SE = standard error.

We used 15 significant IVs and 5 robust analytical MR methods to analyze the effect of IgD^+^ CD38br AC on HF. The OR values of all 4 methods except simple mode were > 1 and statistically significant (*P* < .05) for MR-Egger and IVW, as shown in Figure 2.

#### 3.3.2. Effect of activated Treg AC on HF

Our analysis utilized 5 significant IVs and applied 5 robust analytical MR methods to investigate the effect of Activated Treg AC on HF. OR values of the 5 methods were all <1, and IVW was statistically significant (*P* < .05), as shown in Figure 2.

#### 3.3.3. *Effect of Im myeloid-derived suppressor cell (MDSC) %CD33*^*dim*^ *HLA DR*^−^ *CD66b*^−^ *on HF*

Our analysis involved 7 significant IVs and applied 5 robust analytical MR methods to examine the effect of Im MDSC %CD33^dim^ HLA DR^−^ CD66b^−^ on HF. The OR values for all 5 methods were <1 and statistically significant for WM and IVW (*P* < .05), as detailed in Figure 2.

#### 3.3.4. *Effect of CD33*^*dim*^ *HLA DR*^*+*^ *CD11b*^*+*^ *%CD33*^*dim*^ *HLA DR*^*+*^ *on HF*

The analysis utilized 10 significant IVs and applied 5 robust analytical MR methods to explore the effect of CD33^dim^ HLA DR^+^ CD11b^+^ %CD33^dim^ HLA DR^+^ on HF. The OR values of all 5 methods were < 1 and the IVW was statistically significant (*P* < .05), as shown in Figure 2.

#### 3.3.5. *Effect of double positive (DP) (CD4*^*+*^*CD8*^*+*^*) %leukocyte on HF*

We utilized 13 significant IVs and conducted 5 robust analytical MR methods to assess the effect of DP (CD4^+^CD8^+^) %leukocyte on HF. The OR values from the 5 methods were >1 and the IVW was statistically significant (*P* < .05), detailed in Figure 2.

#### 3.3.6. *Effect of CD28*^−^ *CD127*^−^ *CD25*^*++*^ *CD8br %T cell on HF*

We employed 7 significant IVs and utilized 5 robust analytical MR methods to analyze the effect of CD28^−^ CD127^−^ CD25^++^ CD8br %T cell on HF. The OR values from the 5 methods were > 1 and statistically significant (*P* < .05) for WM and IVW, outlined in Figure 2.

#### 3.3.7. *Effect of CD28*^−^ *CD127*^−^ *CD25*^*++*^ *CD8br %CD8br on HF*

We investigated the effect of CD28^−^ CD127^−^ CD25^++^ CD8br %CD8br on HF using 7 significant IVs and 5 robust analytical MR methods. The OR values of the 5 methods were > 1 and the IVW was statistically significant (*P* < .05), as shown in Figure 2.

#### 3.3.8. *Effect of CD28*^*+*^ *CD45RA*^*+*^ *CD8br %T cell on HF*

We assessed the effect of CD28^+^ CD45RA^+^ CD8br %T cell on HF using 57 significant IVs and 5 robust analytical MR methods. The OR values of all 5 methods were > 1 and MR-Egger and IVW were statistically significant (*P* < .05), as detailed in Figure 2.

#### 3.3.9. *Effect of CD19 on IgD*^*+*^ *CD38br on HF*

We examined the effect of CD19 on IgD^+^ CD38br on HF using 10 significant IVs and 5 robust analytical MR methods. The OR values of the 5 methods were > 1 and the IVW was statistically significant (*P* < .05), as shown in Figure 2.

#### 3.3.10. *Effect of CD20 on IgD*^−^ *CD38*^*dim*^ *on HF*

We investigated the effect of CD20 on IgD^−^ CD38^dim^ on HF utilizing 20 significant IVs and 5 robust analytical MR methods. The OR values of all 5 methods were < 1 and were statistically significant (*P* < .05) for all 4 methods except simple mode, as detailed in Figure 2.

#### 3.3.11. *Effect of CD27 on IgD*^−^ *CD38*^*dim*^ *on HF*

We examined the effect of CD27 on IgD^−^ CD38^dim^ on HF using 16 significant IVs and 5 robust analytical MR methods. The OR values of the 5 methods were > 1 and the IVW was statistically significant (*P* < .05), as shown in Figure 2.

#### 3.3.12. Effect of CD45 on lymphocyte on HF

We analyzed the effect of CD45 on lymphocyte on HF using 9 significant IVs and 5 robust analytical MR methods. The OR values of the 5 methods were > 1 and statistically significant (*P* < .05) for WM and IVW, as detailed in Figure 2.

#### 3.3.13. *Effect of side scatter-A (SSC-A) on CD14*^*+*^ *monocyte on HF*

We investigated the effect of SSC-A on CD14^+^ monocyte on HF using 15 significant IVs and 5 robust analytical MR methods. The OR values of all 5 methods were < 1 and the IVW was statistically significant (*P* < .05), as shown in Figure 2.

#### 3.3.14. *Effect of SSC-A on HLA DR*^*+*^ *natural killer cell (NK) on HF*

We examined the effect of SSC-A on HLA DR^+^ NK on HF using 9 significant IVs and 5 robust analytical MR methods. The OR values of all 5 methods were < 1 and the IVW was statistically significant (*P* < .05), as shown in Figure 2.

#### 3.3.15. *Effect of CD11b on CD14*^*+*^ *monocyte on HF*

We explored the effect of CD11b on CD14^+^ monocyte on HF using 11 significant IVs and 5 robust analytical MR methods. The OR values for all 5 methods were < 1 and statistically significant for WM, IVW and weighted mode (*P* < .05), as detailed in Figure 2.

### 3.4. Sensitivity analyses and visualization of results

In the above 15 MR analyses, all MR-PRESSO global tests did not detect horizontal multidimensional outliers. Subsequently, all MR-Egger intercept tests and Cochran *Q* analyses indicated that the genetic variants had a low likelihood of directional multidimensionality or genetic heterogeneity (Table 2). The results of all the above visualizations are detailed in Figures S1 to S60, Supplemental Digital Content, https://links.lww.com/MD/O980, and importantly, according to the leave-one-out sensitivity analysis plots, by removing each SNP, there is no significant impact on the final results.

**Table 2:** Heterogeneity analysis and directional pleiotropy analysis.

Exposure	Outcome	Cochran *Q* analysis		MR-Egger intercept test	
		MR-Egger	IVW	Egger_intercept	*P*-value
IgD^+^ CD38br AC	HF	*Q* = 11.835	*Q* = 13.633	–0.006	.203
		*P* = .541	*P* = .477		
Activated Treg AC	HF	*Q* = 0.715	*Q* = 0.905	0.005	.691
		*P* = .870	*P* = .924		
Im MDSC %CD33^dim^ HLA DR^−^ CD66b^−^	HF	*Q* = 4.437	*Q* = 4.437	–0.001	.983
		*P* = .488	*P* = .618		
CD33^dim^ HLA DR^+^ CD11b^+^ %CD33^dim^ HLA DR^+^	HF	*Q* = 5.082	*Q* = 5.334	–0.004	.629
		*P* = .749	*P* = .804		
DP (CD4^+^CD8^+^) %leukocyte	HF	*Q* = 7.102	*Q* = 7.668	–0.006	.467
		*P* = .791	*P* = .810		
CD28^−^ CD127^−^ CD25^++^ CD8br %T cell	HF	*Q* = 1.407	*Q* = 1.662	0.003	.635
		*P* = .924	*P* = .948		
CD28^−^ CD127^−^ CD25^++^ CD8br %CD8br	HF	*Q* = 7.562	*Q* = 9.807	0.008	.277
		*P* = .182	*P* = .133		
CD28^+^ CD45RA^+^ CD8br %T cell	HF	*Q* = 70.550	*Q* = 71.992	–0.003	.294
		*P* = .077	*P* = .074		
CD19 on IgD^+^ CD38br	HF	*Q* = 4.243	*Q* = 4.271	0.001	.872
		*P* = .835	*P* = .893		
CD20 on IgD^−^ CD38^dim^	HF	*Q* = 24.848	*Q* = 26.164	0.003	.342
		*P* = .129	*P* = .126		
CD27 on IgD^−^ CD38^dim^	HF	*Q* = 11.004	*Q* = 12.065	0.006	.320
		*P* = .686	*P* = .674		
CD45 on lymphocyte	HF	*Q* = 4.261	*Q* = 4.545	–0.005	.611
		*P* = .749	*P* = .805		
SSC-A on CD14^+^ monocyte	HF	*Q* = 11.204	*Q* = 11.401	0.002	.665
		*P* = .594	*P* = .654		
SSC-A on HLA DR^+^ NK	HF	*Q* = 7.239	*Q* = 7.695	–0.004	.528
		*P* = .404	*P* = .464		
CD11b on CD14^+^ monocyte	HF	*Q* = 9.982	*Q* = 10.288	0.003	.612
		*P* = .352	*P* = .416		

AC = absolute cell, CD = cluster of differentiation, DP = double positive (CD4+CD8+), HF = heart failure, HLA DR = human leukocyte antigen DR, IgD = immunoglobulin D, IVW = inverse variance weighted, MDSC = myeloid-derived suppressor cell, MR = Mendelian randomization, NK = natural killer, SSC-A = side scatter-area.

### 3.5. Impact of HF on immune cells

Using HF as the exposure and 15 immunophenotypes as the outcome, our analysis revealed that the effect of HF on these 15 immunophenotypes was not statistically significant.

## 4. Discussion

Immune cells in the heart are important players in the process of HF and cardiac scarring. Although there is currently no effective cure for HF, immune cell-targeted therapies hold promise as a new avenue of treatment for HF. Through a systematic exploration of extensive publicly available genetic data, we investigated the causal relationships between 731 immunophenotypes and HF. Employing SNPs as IVs, we unveiled significant causal effects between 15 immunophenotypes across 4 immune profiles (AC, RC, MFI, and MP) and HF through bidirectional MR studies. Specifically, 8 immunophenotypes were associated with an increased risk of HF, including IgD^+^ CD38br AC, DP (CD4^+^CD8^+^) %leukocyte, CD28^−^ CD127^−^ CD25^++^ CD8br %T cell, CD28^−^ CD127^−^ CD25^++^ CD8br %CD8br, CD28^+^ CD45RA^+^ CD8br %T cell, CD19 on IgD^+^ CD38br, CD27 on IgD^−^ CD38^dim^, and CD45 on lymphocyte. Conversely, 7 immunophenotypes showed an association with a reduced risk of HF, including Activated Treg AC, Im MDSC % CD33^dim^ HLA DR^−^ CD66b^−^, CD33^dim^ HLA DR^+^ CD11b^+^ %CD33^dim^ HLA DR^+^, CD20 on IgD^−^ CD38^dim^, SSC-A on CD14^+^ monocyte, SSC-A on HLA DR^+^ NK, and CD11b on CD14^+^ monocyte.

HF is a chronic and persistent inflammatory process that indicates a disturbance of the body’s immune mechanisms, so controlling the inflammatory response due to autoimmunity is crucial for the heart. Tregs are considered pivotal suppressors of the immune response.^[29,30]^ In line with our findings, Bansal^[31]^ highlighted the role of partially dysfunctional Tregs in promoting immune activation and pathologic left ventricular remodeling in mice with HF due to myocardial infarction. Restoring Treg functionality emerges as a potential therapeutic avenue. Similarly, our study indicated positive correlations of CD28^−^ CD127^−^ CD25^++^ CD8br %T cells, CD28^−^ CD127^−^ CD25^++^ CD8br %CD8br, and CD28^+^ CD45RA^+^ CD8br %T cells with HF, whereas functionally normal Activated Treg ACs could potentially mitigate the inflammatory response associated with HF. MDSCs are myeloid-derived cells known for their immunosuppressive abilities,^[32,33]^ expanding typically in tumor microenvironments or inflammatory settings to regulate the inflammatory response and safeguard damaged tissues.^[34]^ Zhou et al^[35]^ found that cytokines secreted by MDSC inhibited cardiomyocyte hypertrophy and expression of pro-inflammatory genes in mice with hypertension-induced HF. Hence, elevating the activity of Im MDSC % CD33^dim^ HLA DR^−^ CD66b^−^ and CD33^dim^ HLA DR^+^ CD11b^+^ %CD33^dim^ HLA DR^+^ via targeted therapy emerges as a promising strategy. In the post-injury phase, persistent interstitial fibrosis and cardiac contractile dysfunction prevail, with B cells playing a pivotal role.^[36,37]^ B cells, besides antibody production,^[38]^ act as antigen-presenting cells^[39]^ participating in immune regulation by cytokine production and upregulation of surface co-stimulatory molecules.^[40]^ These mechanisms encompass inflammatory cytokine secretion,^[41]^ monocyte recruitment^[42]^ and interaction with CD4^+^ Th cells, amplifying the inflammatory response.^[43]^ Immunophenotypes such as IgD^+^ CD38br AC, CD19 on IgD^+^ CD38br, and CD27 on IgD^−^ CD38^dim^ positively correlated with HF, warranting focused attention. Monocyte recruitment stands as a key factor in HF progression. Monocytes, integral components of innate immunity, differentiate into macrophages.^[44]^ Among these, M1-type macrophages secrete pro-inflammatory cytokines, rapidly phagocytose pathogens, and mediate tissue damage, promoting immune-inflammatory responses.^[45]^ Conversely, M2-type macrophages primarily exhibit anti-inflammatory roles, contributing to myocardial tissue recovery.^[46]^ Our study pinpointed SSC-A on CD14^+^ monocyte and CD11b on CD14^+^ monocyte as potential treatment targets for HF. Pressure overload is one of the main reasons for the development of HF, in which a subset of immune cells promotes cardiac hypertrophy and fibroblast activation, whereas NK cells have cardioprotective activity. Therefore, enhancing the expression of SSC-A on HLA DR^+^ NK could treat HF to some extent.^[47]^ Finally, since rs115642223, rs111856210, and rs1801274 can act as regulators of a wide range of immune cells, we believe that they may be key loci for intervention in HF. However, how to intervene at the genetic level and ultimately maximize the benefit for HF patients is a question worth pondering.

Notably, HF of different etiologies (ischemic vs nonischemic) and hemodynamic phenotypes (“cold + wet” and “warm + dry”) may exhibit distinct immunophenotypic profiles. For instance, ischemic HF, often driven by coronary artery disease, may involve stronger inflammatory responses mediated by pro-inflammatory immune cells such as M1-type macrophages and CD28^−^ CD127^−^ CD25^++^ CD8br %T cells, whereas nonischemic HF (e.g., due to hypertension or diabetic cardiomyopathy) might involve adaptive immune dysregulation, such as Treg depletion (linked to reduced Activated Treg AC in our results) or B-cell-mediated fibrosis.^[48]^ Additionally, the hemodynamic phenotypes of HF could further modulate immune responses. For example, the “warm + dry” phenotype appears to involve B-cell-driven mechanisms (e.g., CD19 on IgD^+^ CD38br), where autoantibodies may contribute to diastolic dysfunction.^[49,50]^ However, our current study did not stratify HF by etiology or hemodynamic phenotype due to data limitations. Future research should explore these subgroups to identify tailored immunomodulatory therapies.

Our study has several advantages. First, MR analysis eliminates the disadvantages of observational studies, such as the inability to exclude confounding factors and reverse causality. Second, thanks to the large-scale and nonoverlapping GWAS, we have included more immunophenotypes in the study and obtained more reliable results than previous studies. Finally, the reverse MR analyses show that immunophenotypes have little effect on HF, greatly enhancing the robustness of our study results. This study confirms from a genetic level that multiple immunophenotypes are involved in the development of HF, which is expected to become a new target for drug development. In recent years, immunotherapy has become a research hotspot, including immune activators, immune suppressors, immune cell therapy, therapeutic antibodies, vaccines, and immune system modulators. In the future, immune activators or immune suppressors can be developed specifically for diseases related to immunophenotypes; the expression of immunophenotypes can also be controlled through the active components of traditional Chinese medicine; finally, immunophenotypes can be separated from the patient’s or donor’s body, activated in vitro, and then infused back into the patient’s body to treat HF.

Nevertheless, our study has limitations. First, horizontal pleiotropy, despite numerous sensitivity analyses, cannot be entirely assessed. Second, our study is based on a European population, and whether it can be generalized to other populations remains to be proved by more reliable evidence. For example, thrombosis susceptibility genes vary considerably across populations, so even for the same disease, MR analyses will not yield identical results due to different genetic backgrounds.^[51]^ However, as GWAS data on immune cells and HF continue to evolve, a variety of positive results differentiated by populations are bound to appear in the future. Third, because the GWAS database spans a wide range of sexes, ages, and disease severity, and does not exclude patients with co-morbidities (diabetes, chronic kidney disease, atrial fibrillation, etc), this further limits the generalization of the findings. Additionally, our focus primarily on the effect of immunophenotypes on HF led us to perform reverse MR analysis for only 15 positive results. The influence of HF on immunophenotypes requires further investigation. Lastly, using a looser threshold for assessment without conducting the false discovery rate test may have increased some false-positive results but provided a comprehensive overview of the interplay between immunophenotypes and HF.

## 5. Conclusion

Immune cells in the heart are important players in the process of HF and cardiac scarring. Although there is currently no effective cure for HF, immune cell-targeted therapies hold promise as a new avenue of treatment for HF. The results of this MR study suggest that a variety of immune cells play a key role in HF, but caution is still needed regarding potential clinical applications. Immunomodulation is a promising therapeutic approach as it can be highly selective by specifically inhibiting or enhancing molecular targets. However, at the same time, this can be a potential pitfall as multiple redundancies within the immune system may bypass the desired effect produced by the treatment, which needs to be kept in mind when designing and investigating targeted therapies against specific molecular targets within the immune signaling pathways. In conclusion, our bidirectional MR analysis assessed the causal relationship between 731 immune phenotypes and HF, utilizing a large number of samples and reducing confounding factors, providing valuable insights into HF treatment strategies. However, further studies with rigorous experiments are needed in the future to determine the specific ways in which the above immune cells protect or damage the heart and to determine whether immunomodulatory therapies could be the best option for treating HF. Of course, HF of different etiologies may respond differently to targeted therapies, and future studies should take this into account.

## Author contributions

**Conceptualization:** Zhigang Sun.

**Data curation:** Weibo Zhong.

**Funding acquisition:** Dandan Guo.

**Investigation:** Xihao Chen, Haohong Zheng.

**Methodology:** Xiaohan Xiu.

**Project administration:** Dandan Guo.

**Resources:** Jixin Li.

**Software:** Zhenyu Yang.

**Supervision:** Dandan Guo.

**Validation:** Fengzhao Liu.

**Visualization:** Zhenyu Yang.

**Writing – original draft:** Zhenyu Yang, Jixin Li.

**Writing – review & editing:** Jixin Li, Fengzhao Liu, Xiaohan Xiu, Weibo Zhong, Zhigang Sun, Xinyu Zhu, Mengzhu Chen, Xihao Chen, Haohong Zheng, Dandan Guo.

## Supplementary Material


