# Supplementary material for: Causal analysis of 731 immunophenotypes and heart failure: A bidirectional Mendelian randomization study
Source: Medicine (Baltimore). 2025 May 23;104(21):e42530. doi: 10.1097/MD.0000000000042530 (PMC12114048; doi:10.1097/MD.0000000000042530)

Figure S1: Leave-one-out sensitivity analysis plot of the effect of IgD⁺ CD38br AC on HF.

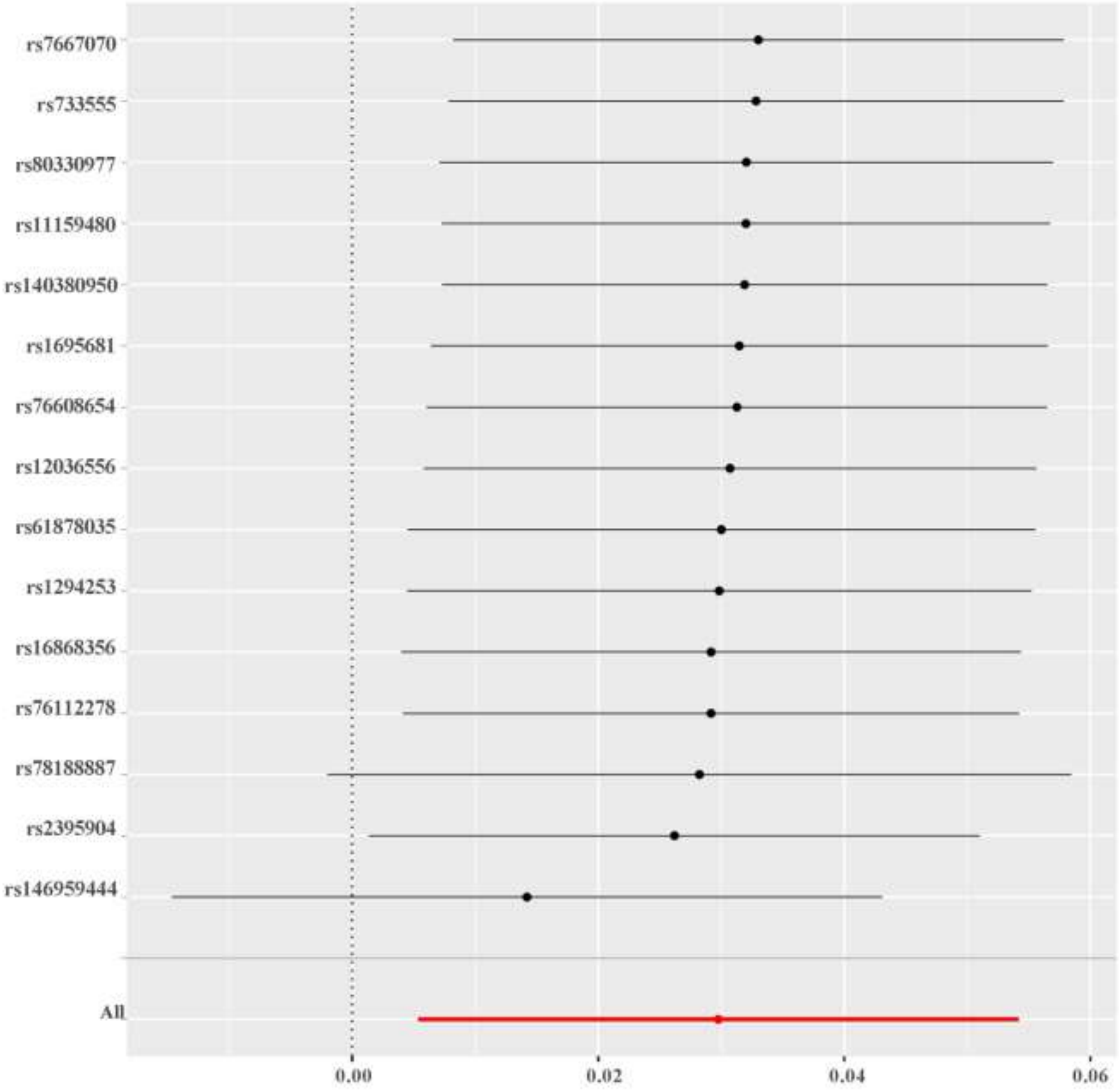

Figure S2: Funnel plot of the effect of IgD^{+} CD38br AC on HF.

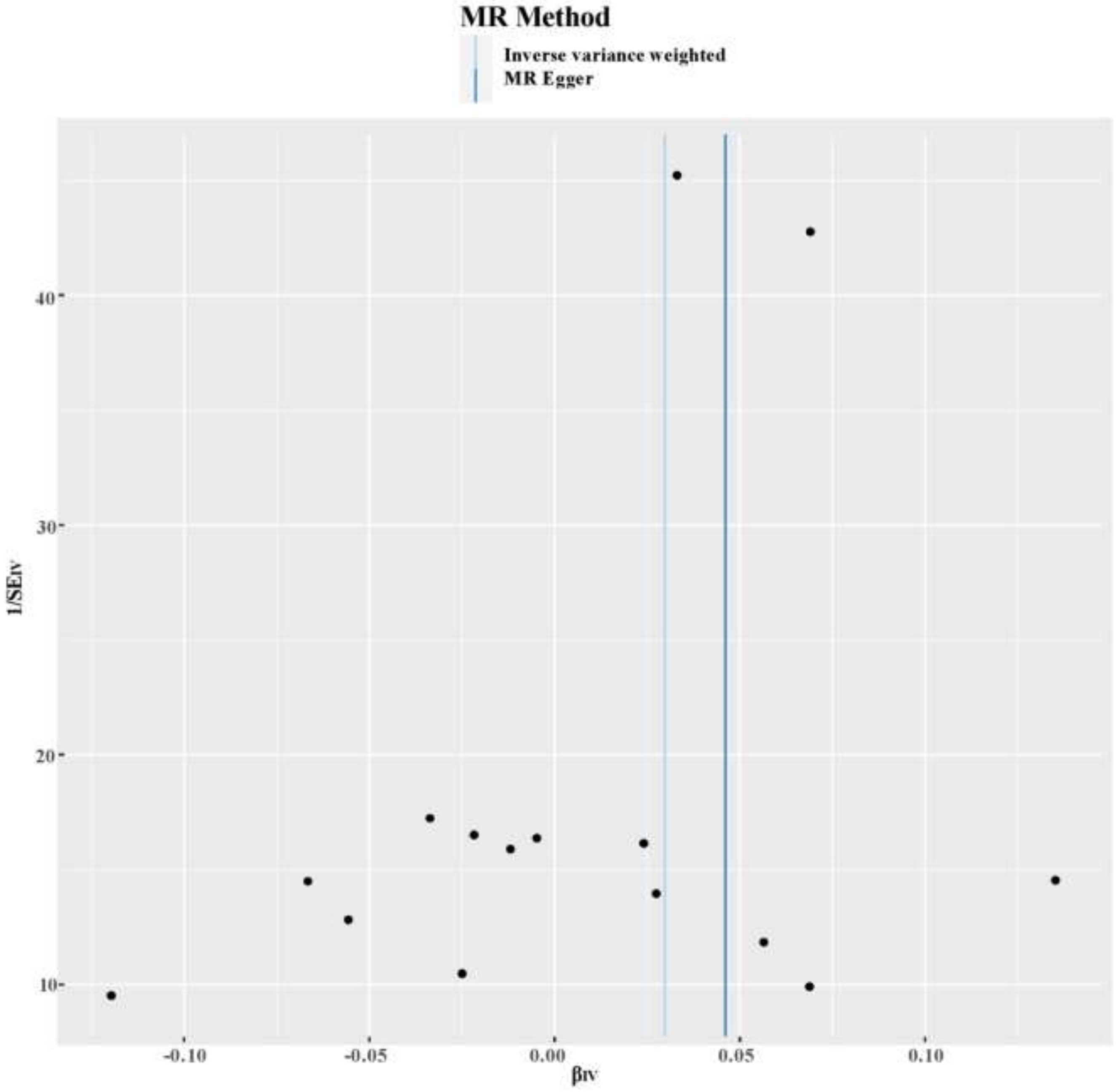

Figure S3: Scatter plot of the effect of IgD⁺ CD38br AC on HF.

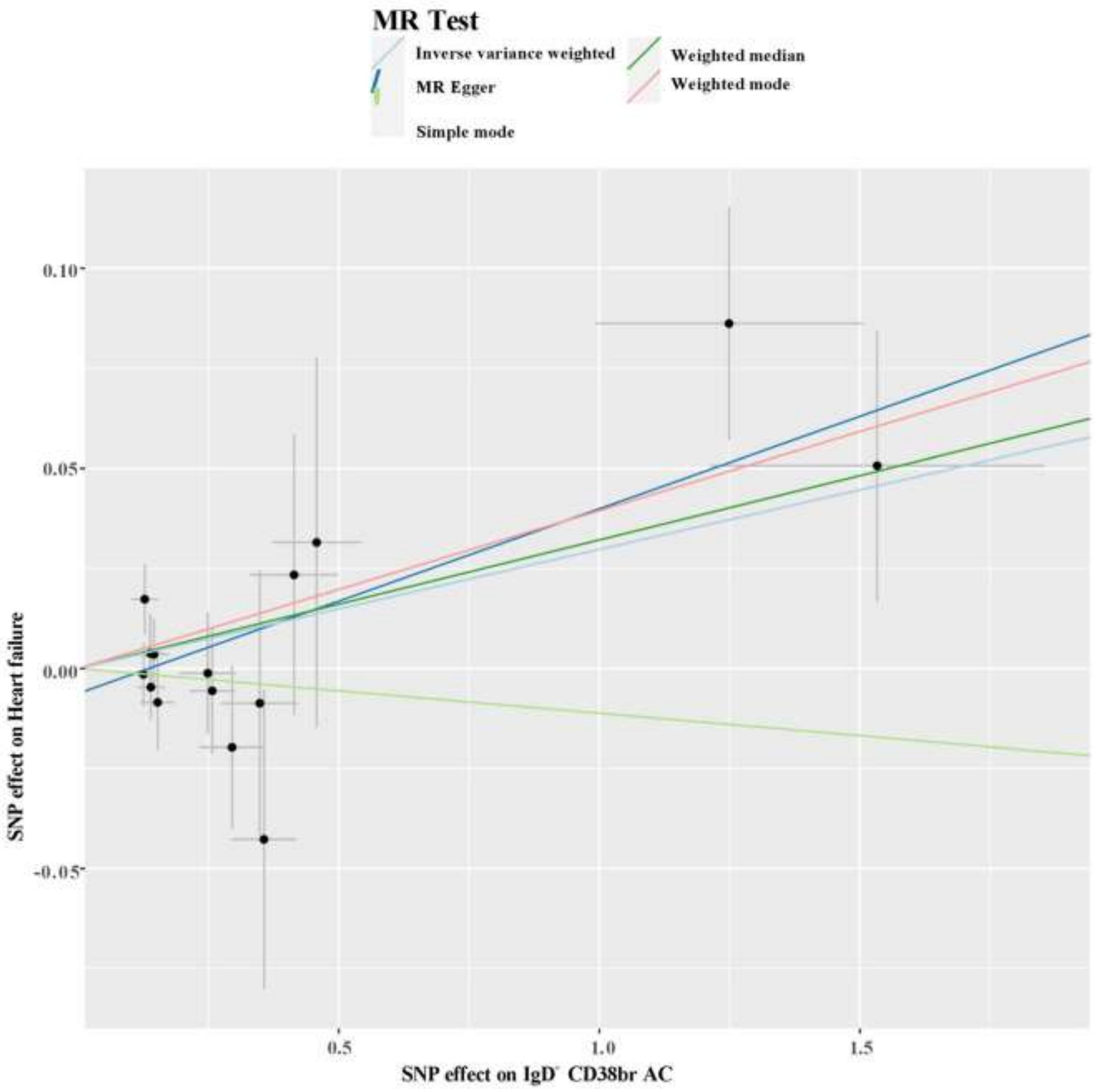

Figure S4: Forest plot of the effect of IgD^{+} CD38br AC on HF.

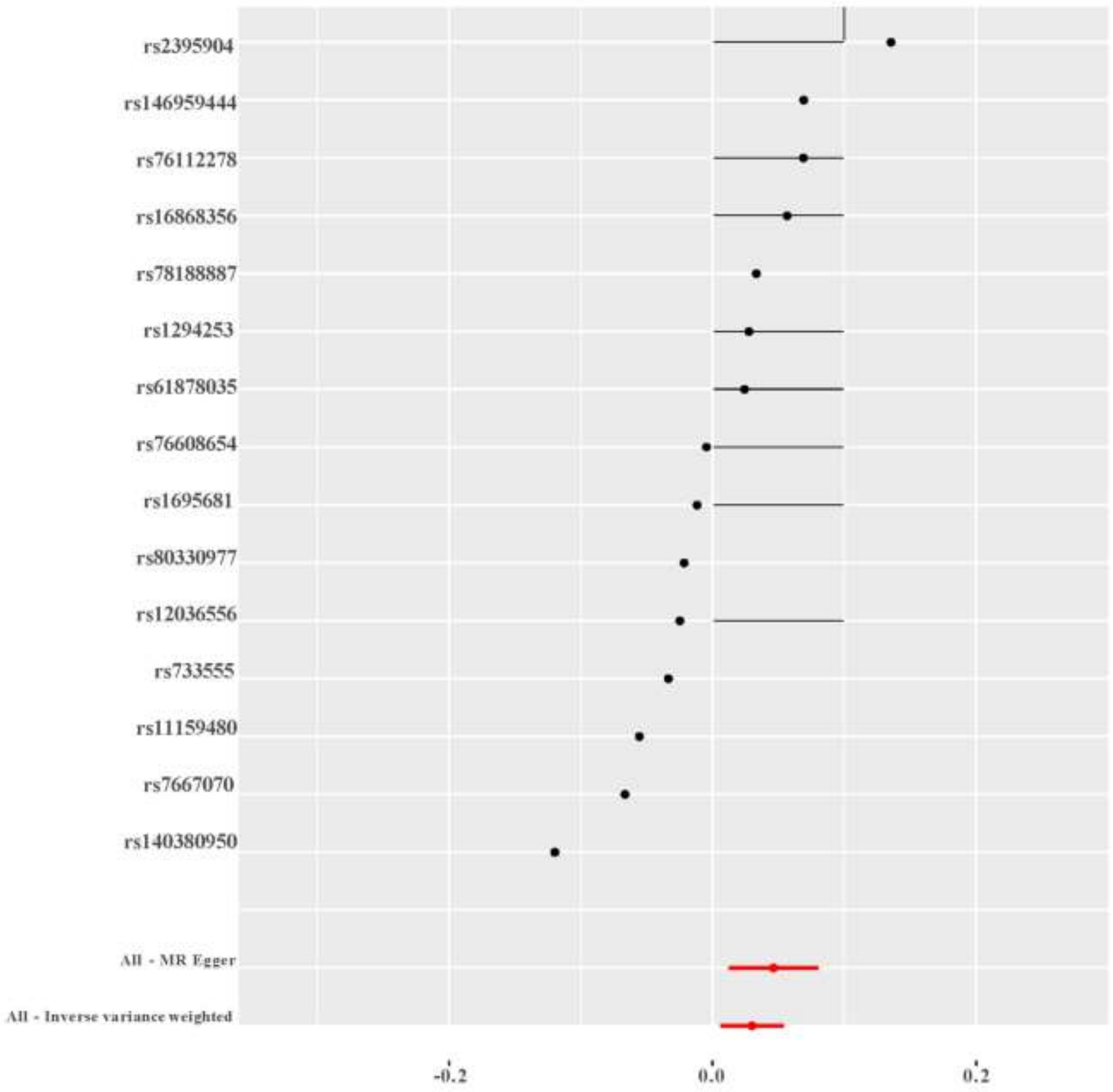

Figure S5: Leave-one-out sensitivity analysis plot of the effect of Activated Treg AC on HF.

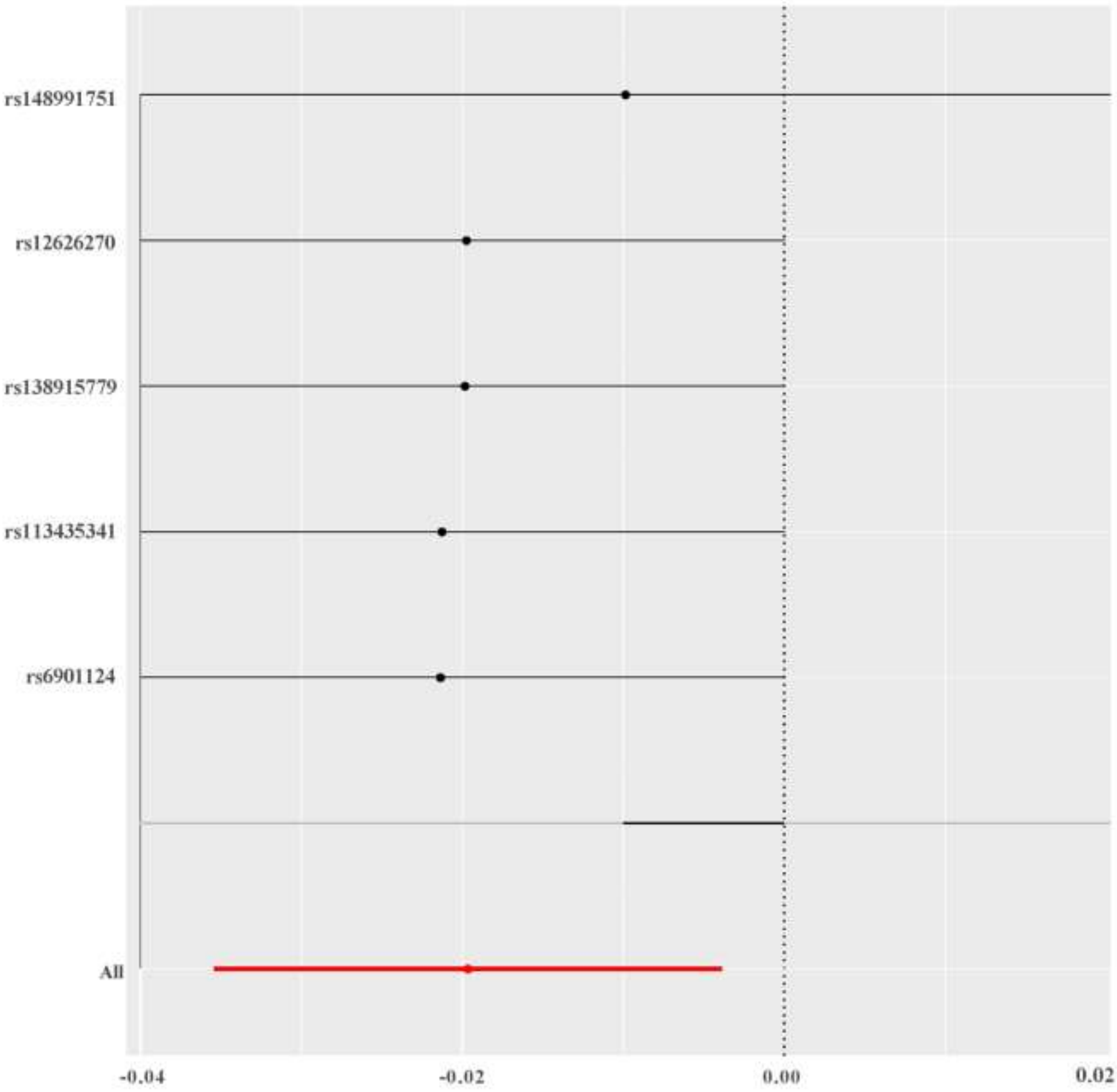

Figure S6: Funnel plot of the effect of Activated Treg AC on HF.

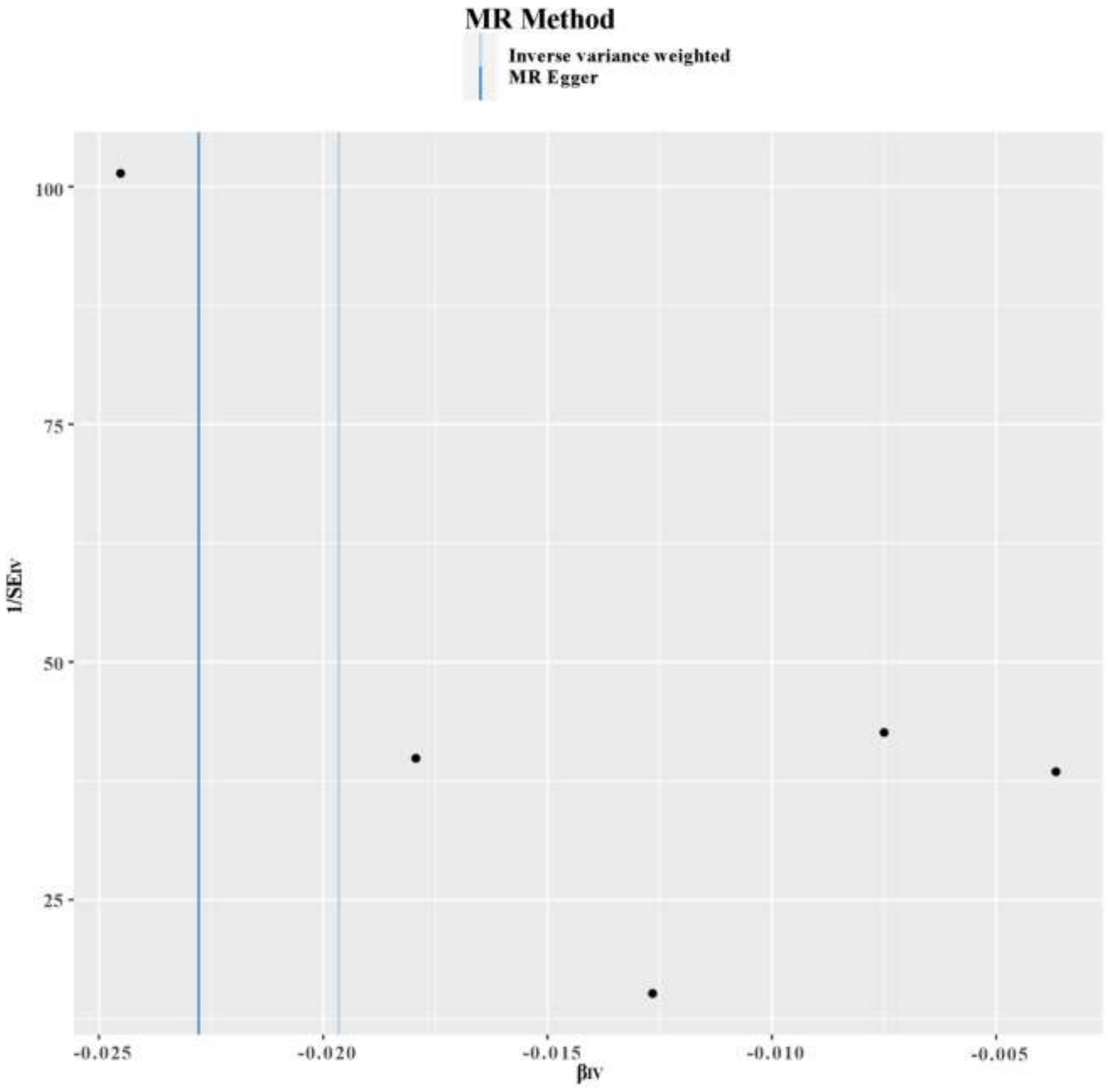

Figure S7: Scatter plot of the effect of Activated Treg AC on HF.

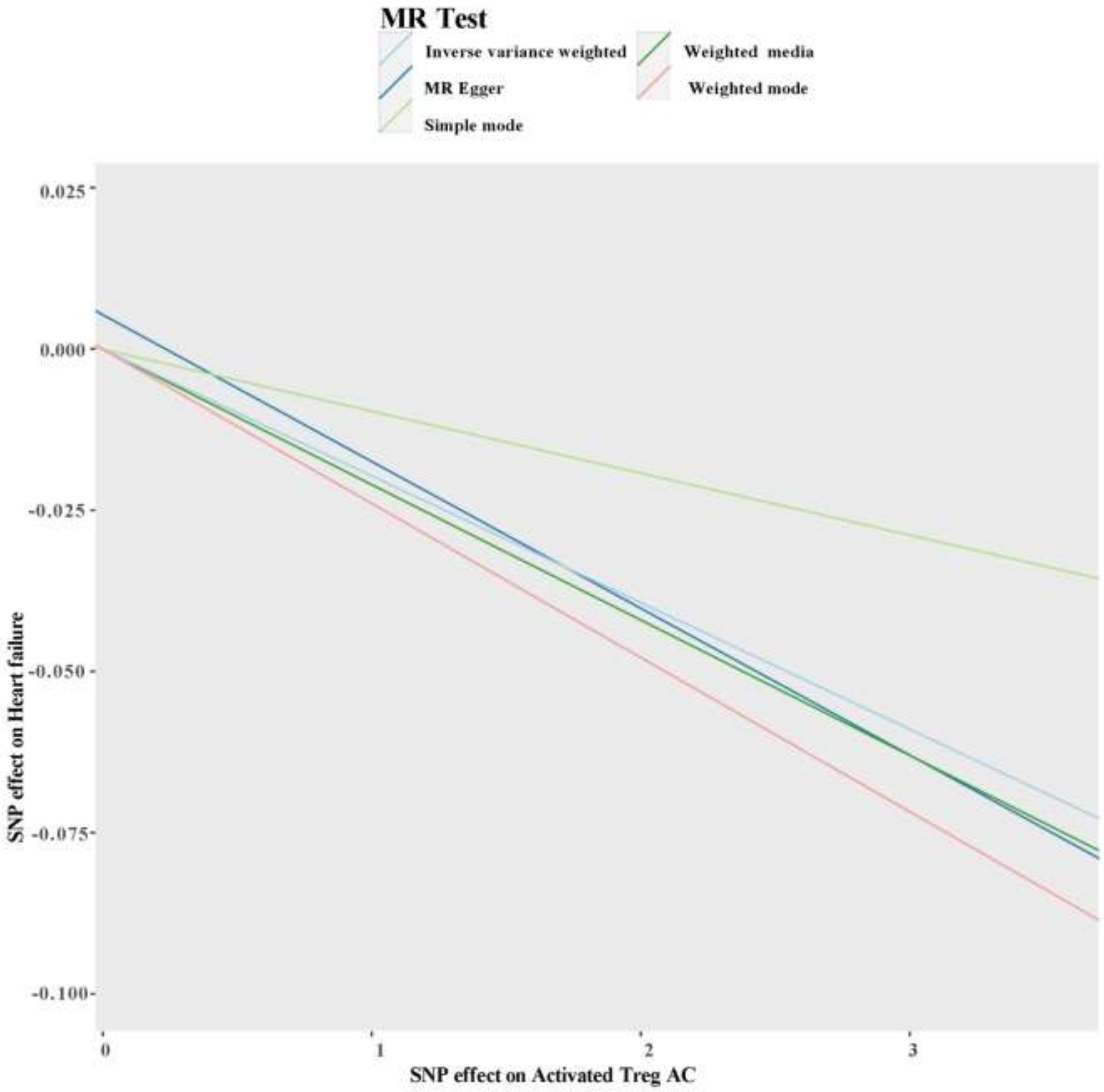

Figure S8: Forest plot of the effect of Activated Treg AC on HF.

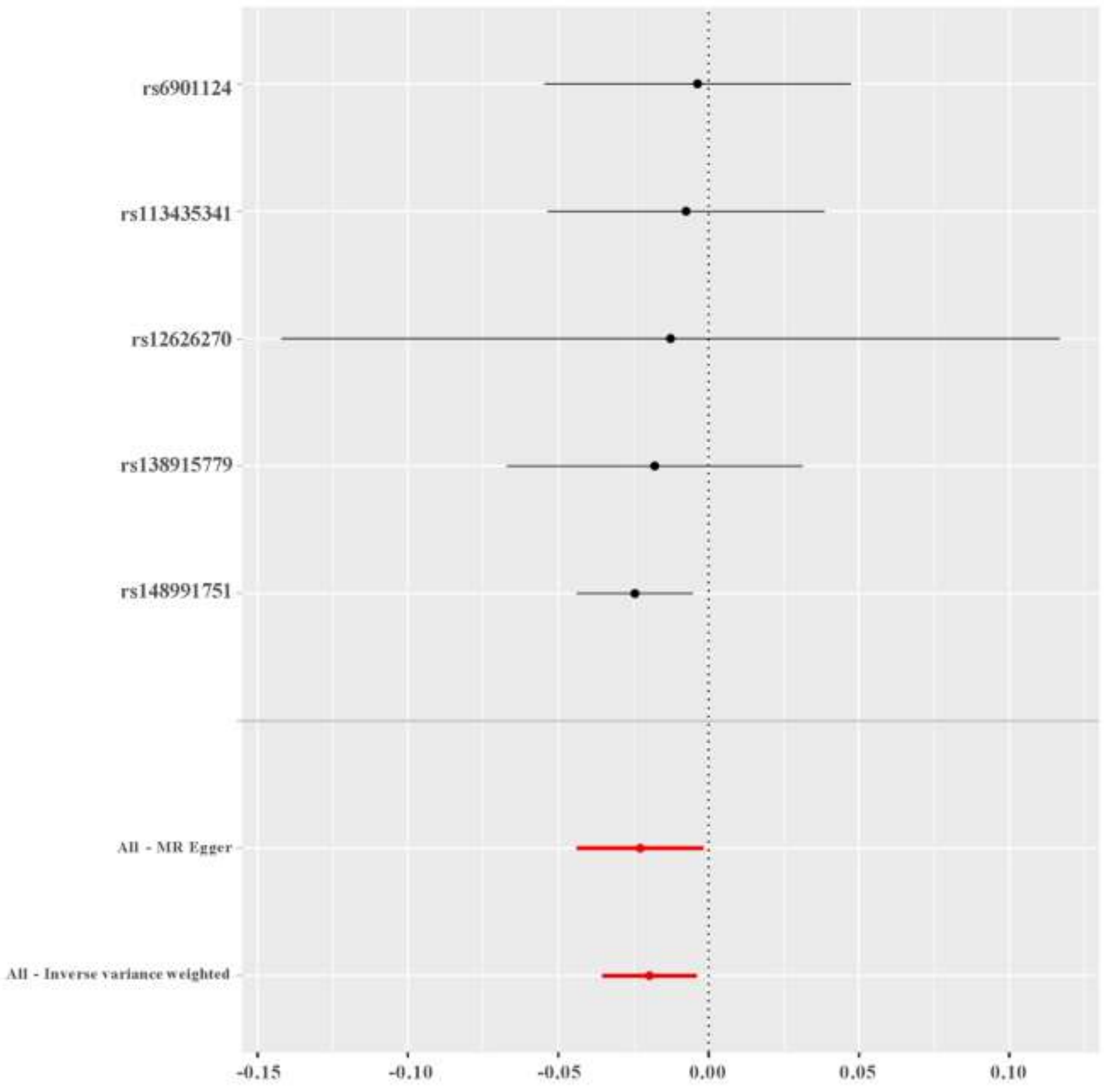

Figure S9: Leave-one-out sensitivity analysis plot of the effect of Im MDSC %CD33^{dim} HLA DR^{-} CD66b^{-} on HF.

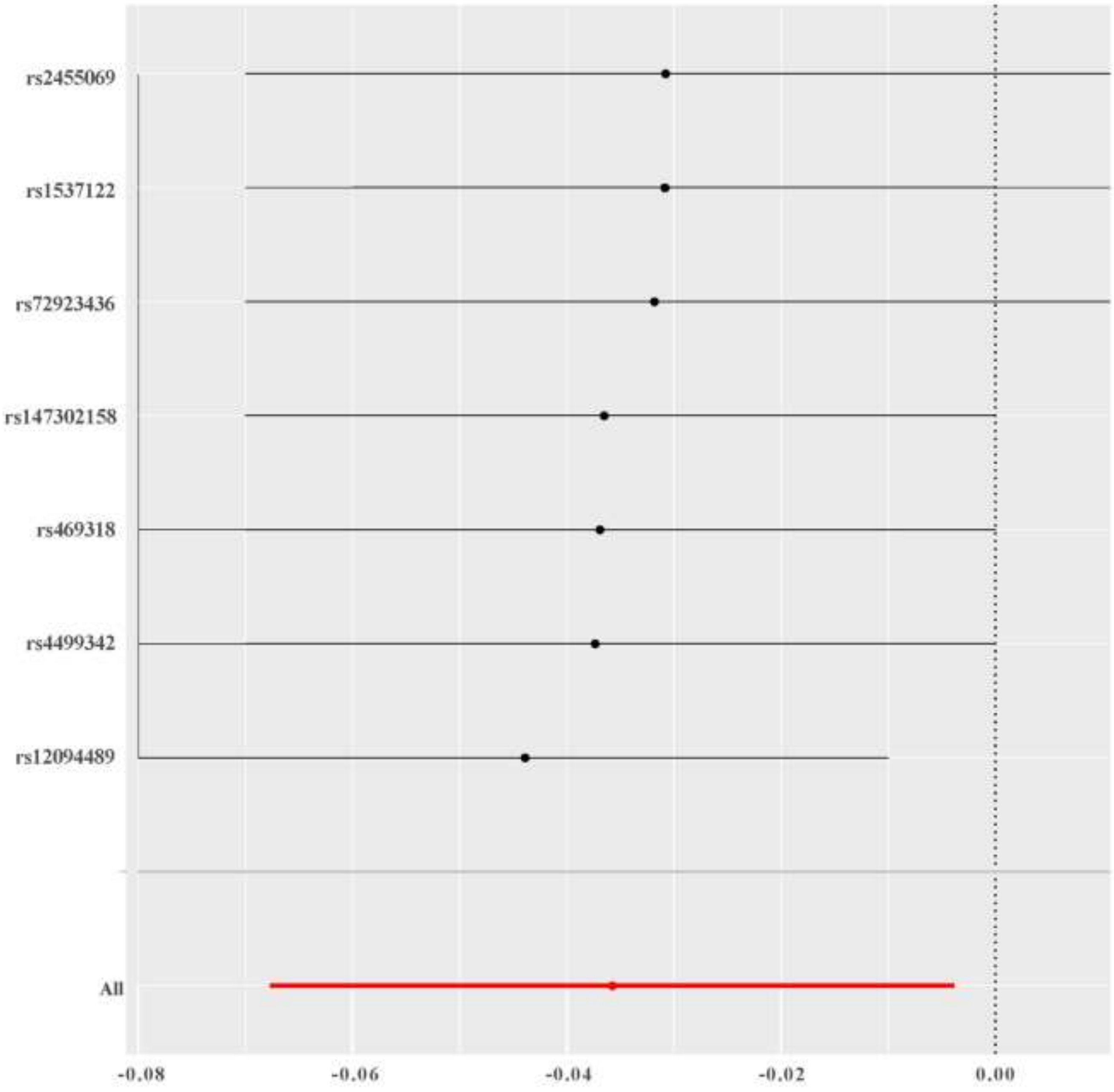

Figure S10: Funnel plot of the effect of Im MDSC %CD33^{dim} HLA DR^{-} CD66b^{-} on HF.

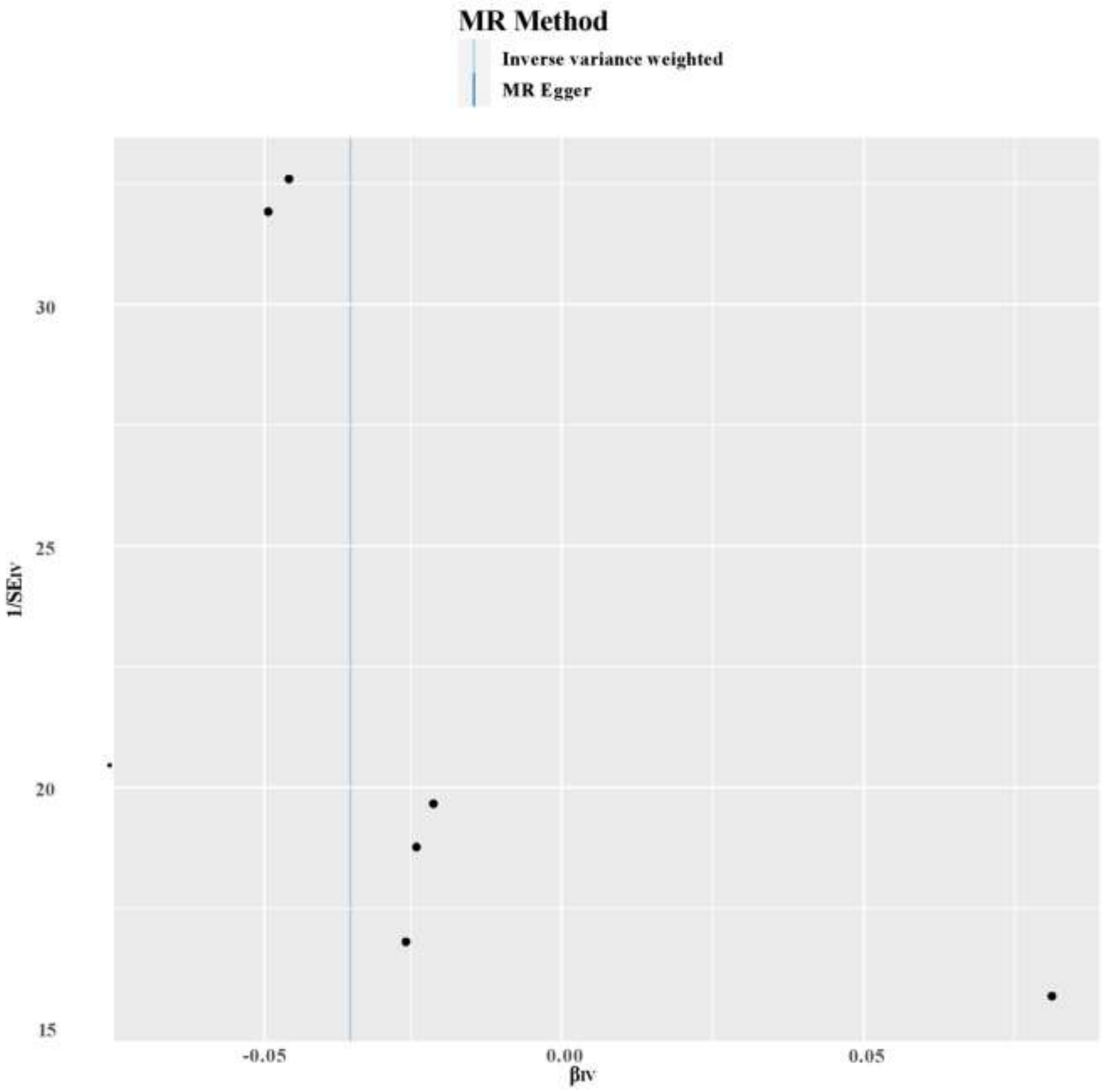

Figure S11: Scatter plot of the effect of Im MDSC %CD33^{dim} HLA DR^{-} CD66b^{-} on HF.

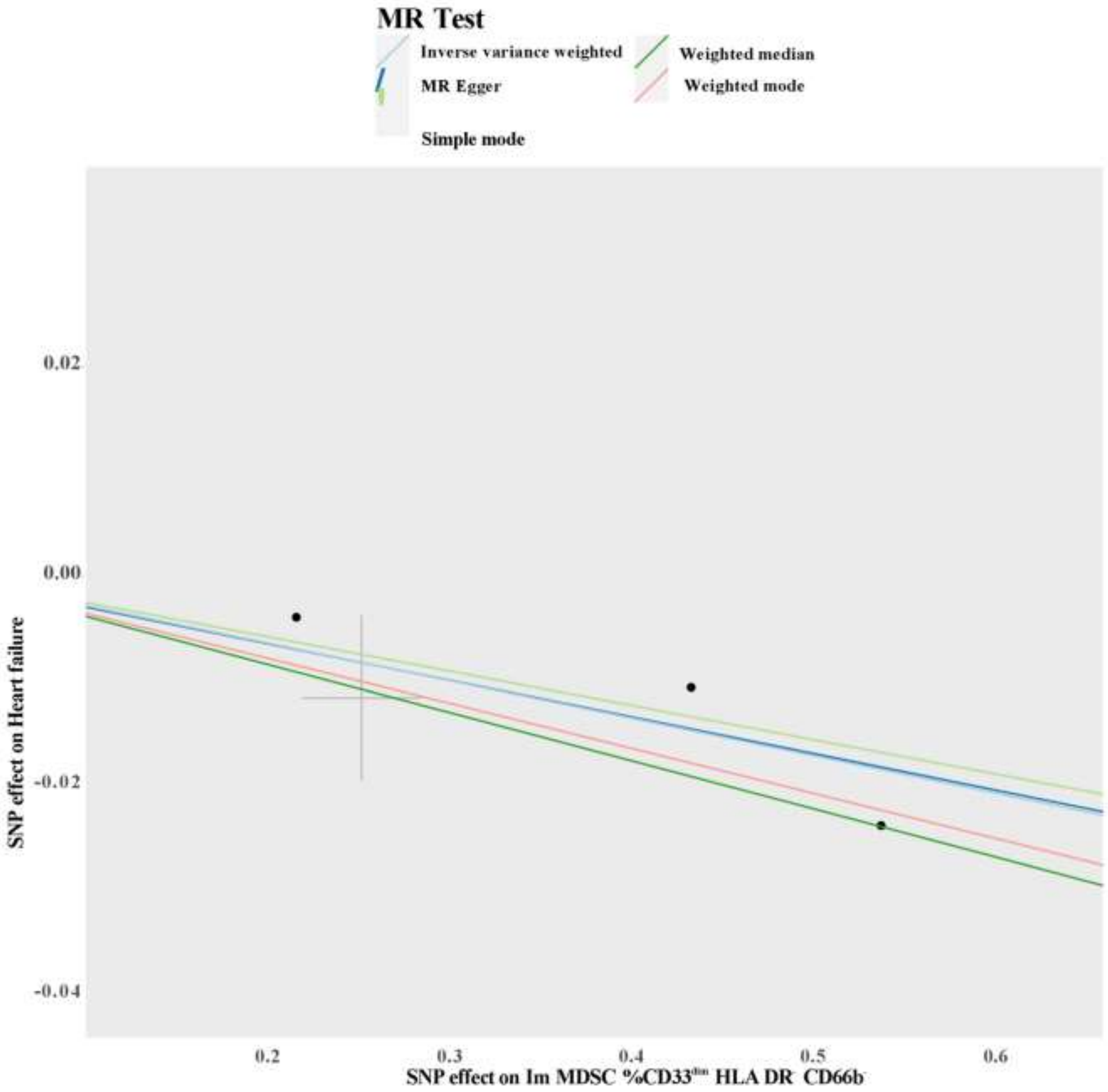

Figure S12: Forest plot of the effect of Im MDSC %CD33^{dim} HLA DR^{-} CD66b^{-} on HF.

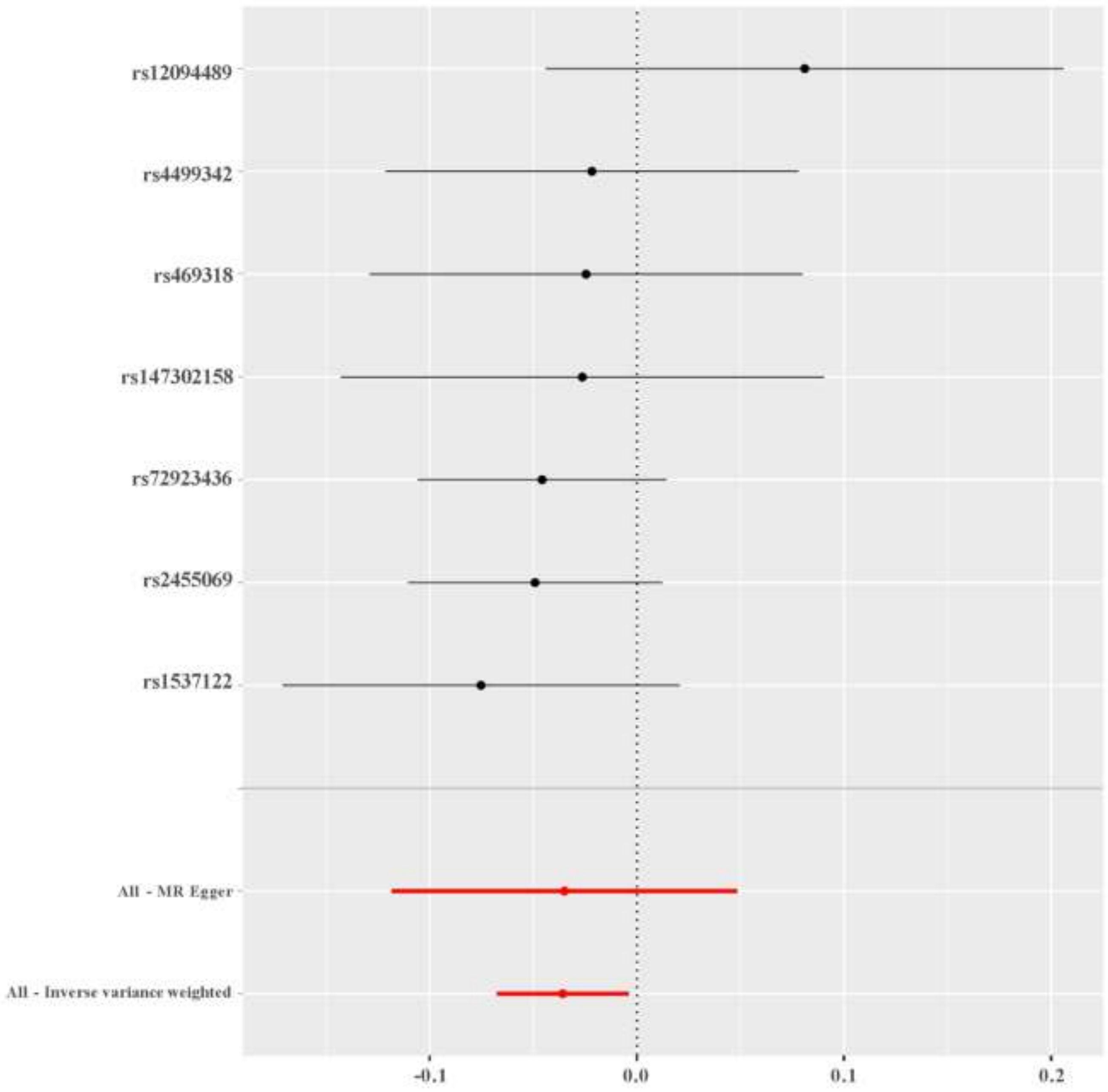

Figure S13: Leave-one-out sensitivity analysis plot of the effect of CD33^{dim} HLA DR^{+} CD11b^{+} %CD33^{dim} HLA DR^{+} on HF.

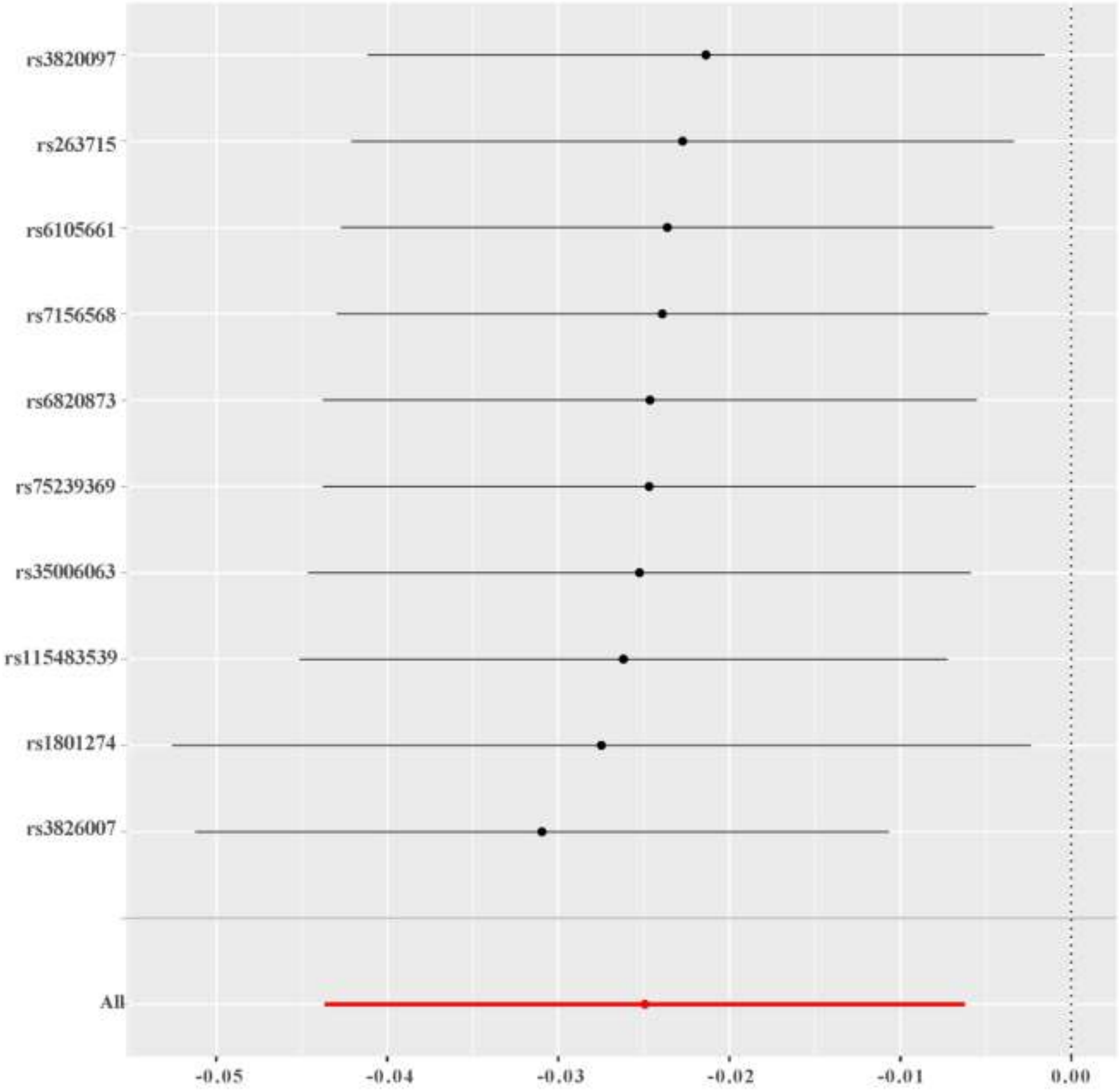

Figure S14: Funnel plot of the effect of CD33^{dim} HLA DR^{+} CD11b^{+} %CD33^{dim} HLA DR^{+} on HF.

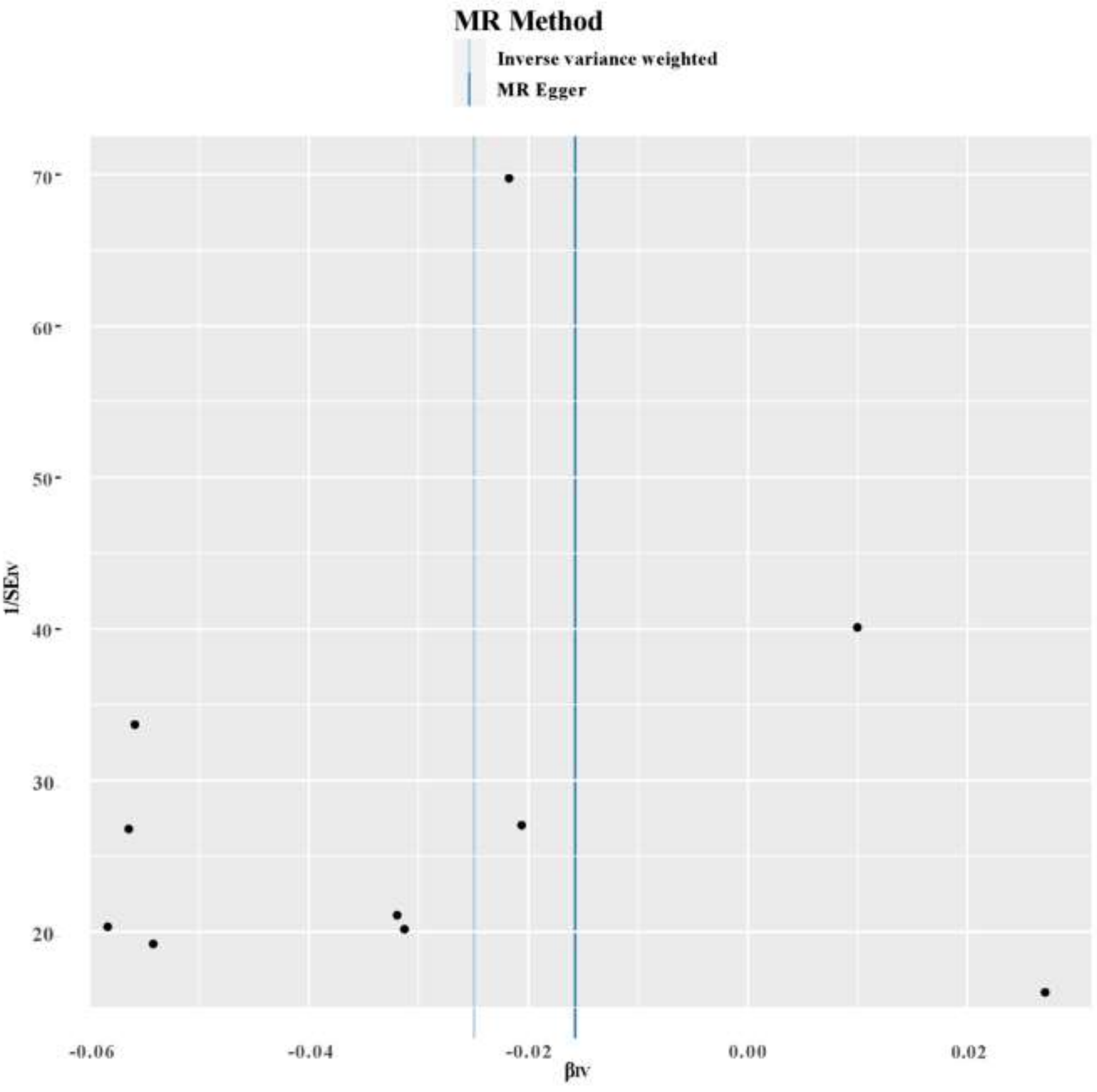

Figure S15: Scatter plot of the effect of CD33^{dim} HLA DR^{+} CD11b^{+} %CD33^{dim} HLA DR^{+} on HF.

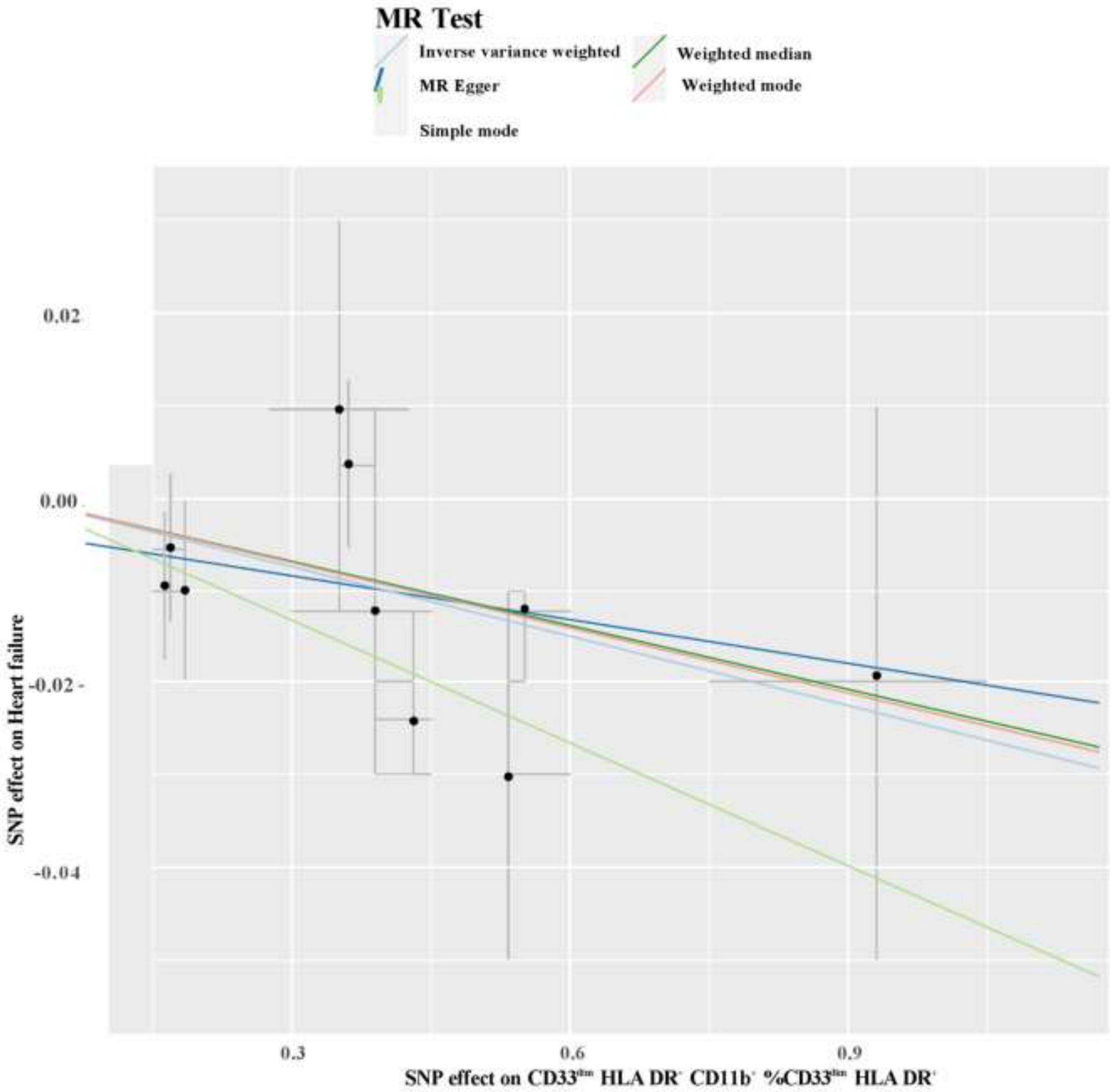

Figure S16: Forest plot of the effect of $CD33^{dim}$ HLA DR^{+} $CD11b^{+}$ %$CD33^{dim}$ HLA DR^{+} on HF.

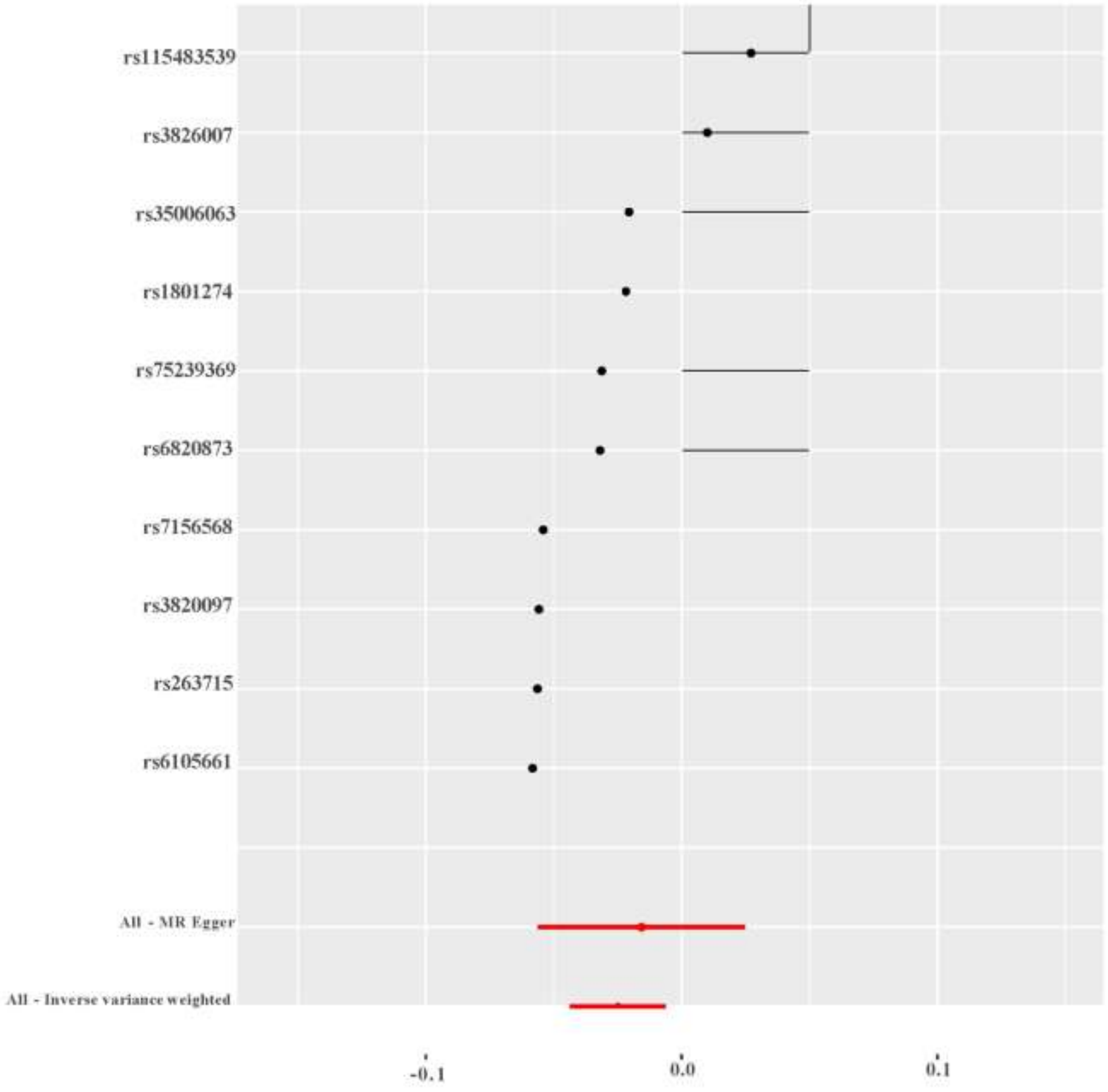

Figure S17: Leave-one-out sensitivity analysis plot of the effect of DP ($CD4^{+}CD8^{+}$) %leukocyte on HF.

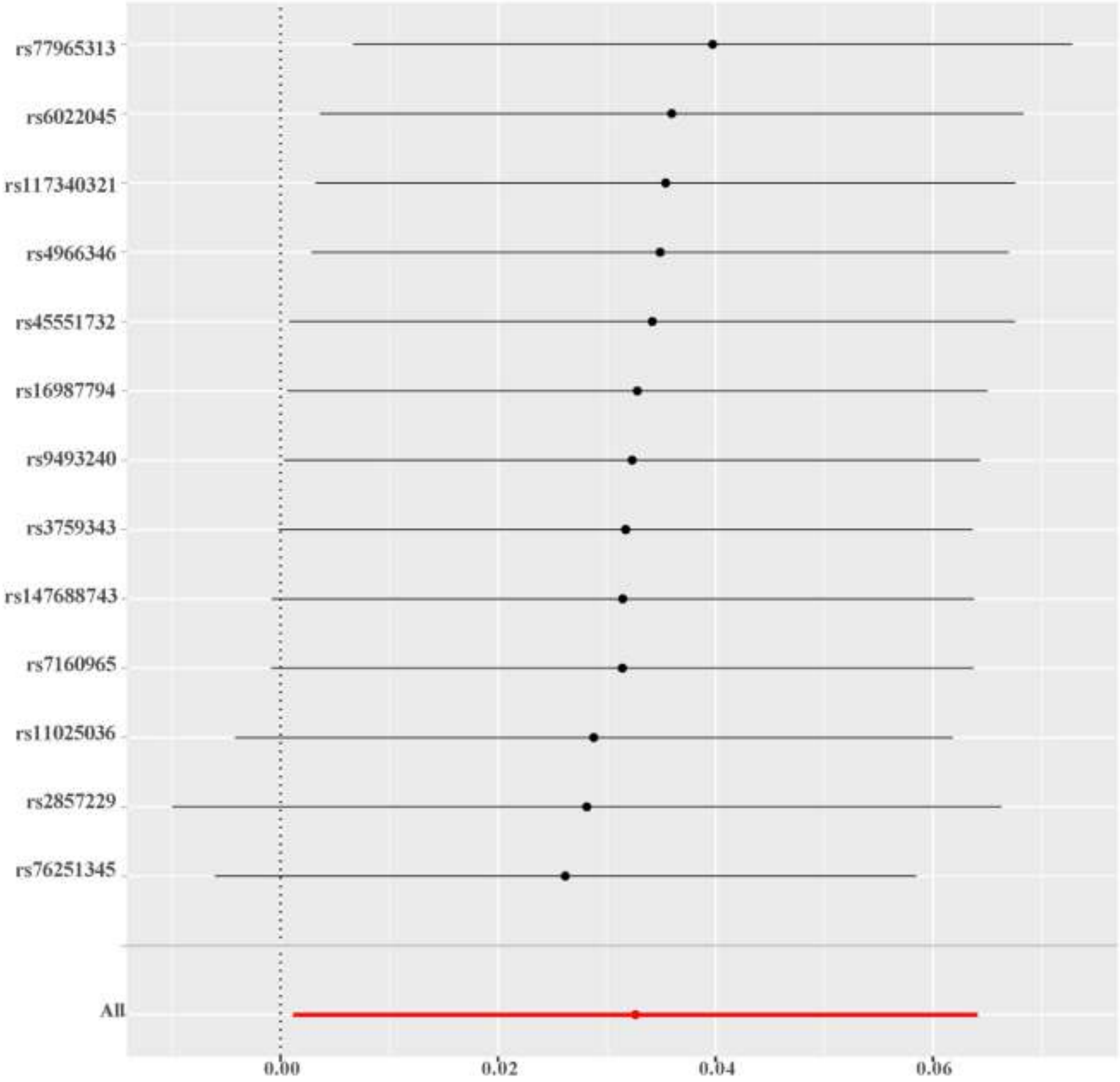

Figure S18: Funnel plot of the effect of DP ($CD4^{+}CD8^{+}$) %leukocyte on HF.

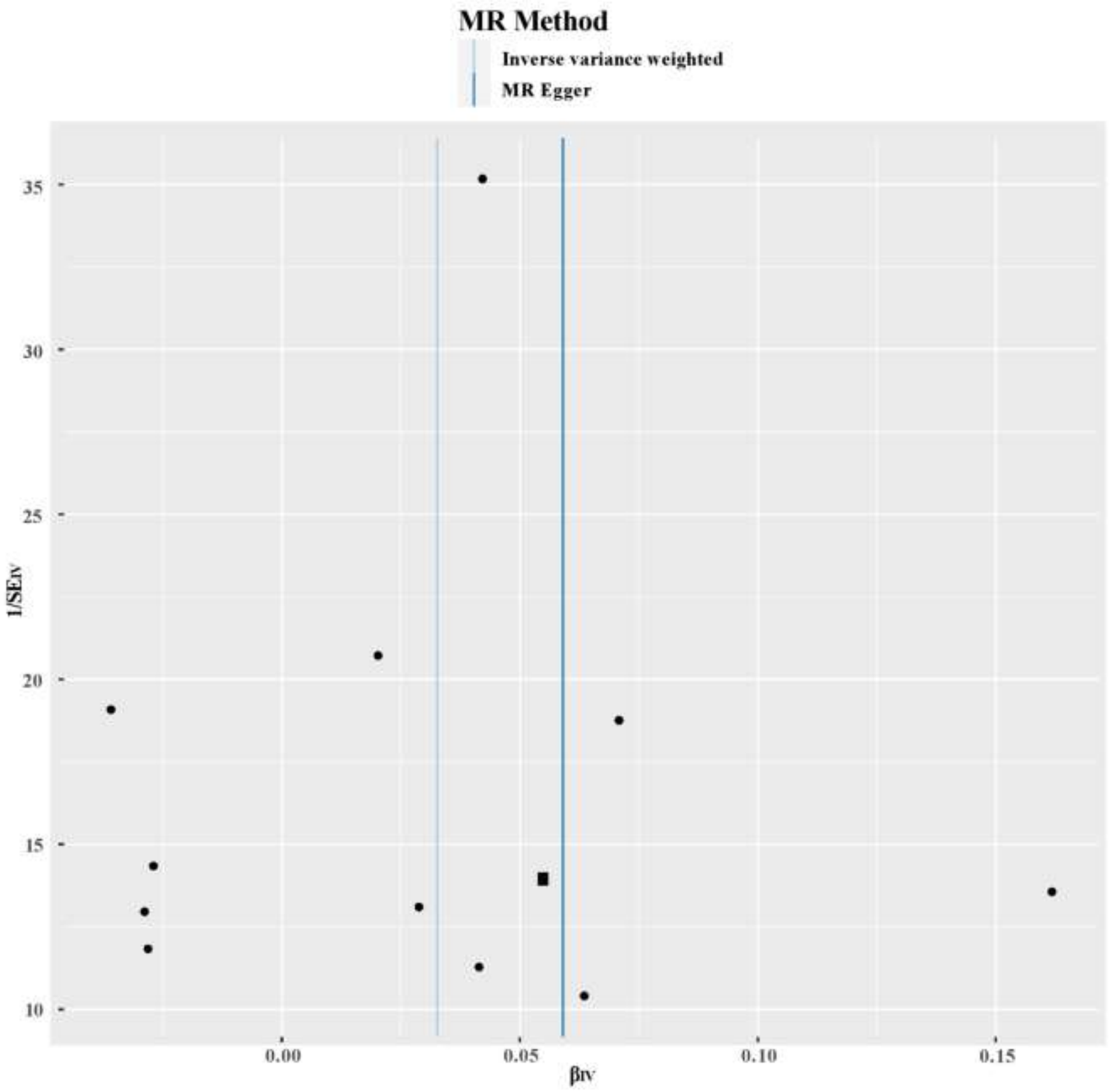

Figure S19: Scatter plot of the effect of DP ($CD4^{+}CD8^{+}$) %leukocyte on HF.

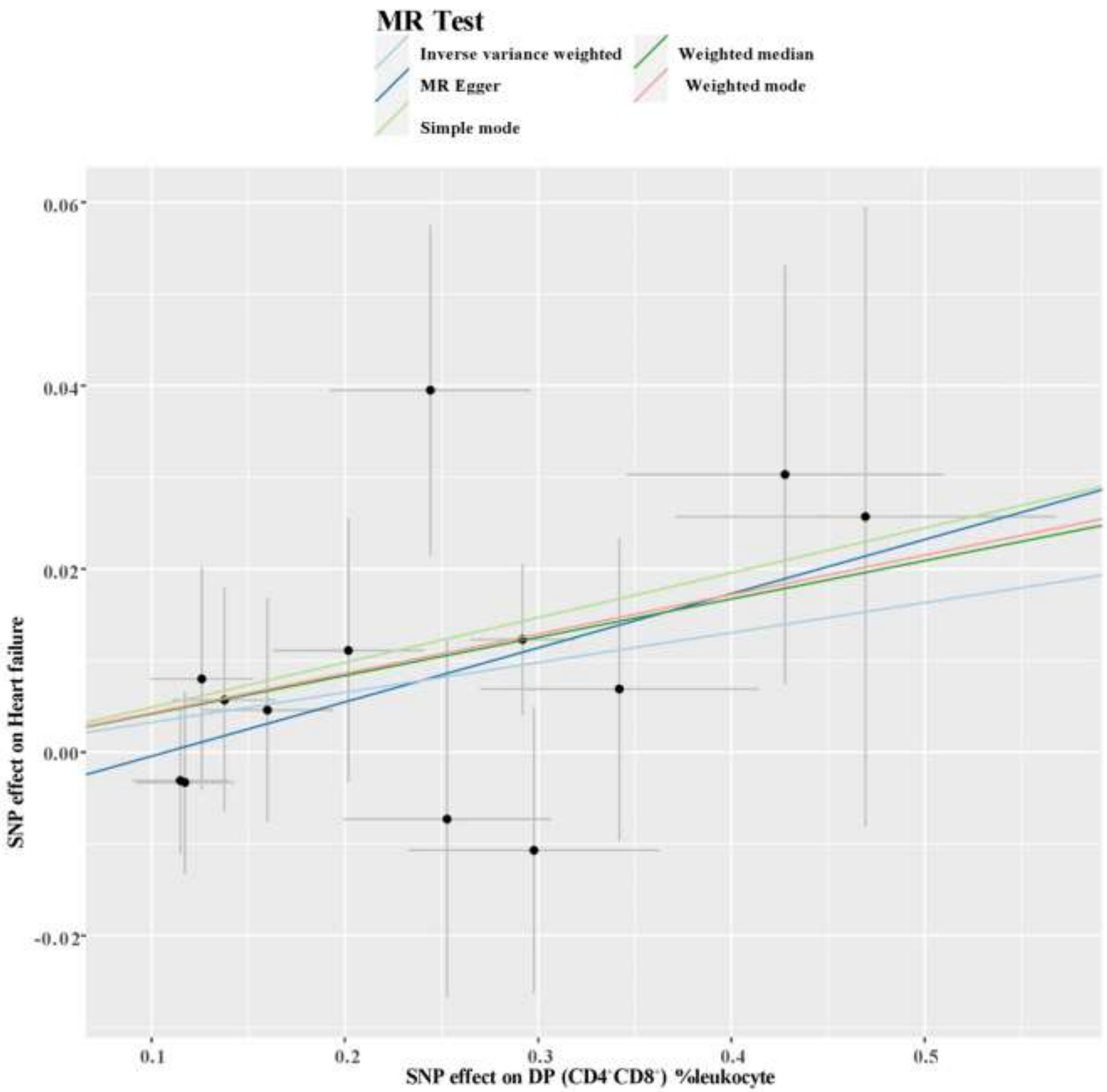

Figure S20: Forest plot of the effect of DP ($CD4^{+}CD8^{+}$) %leukocyte on HF.

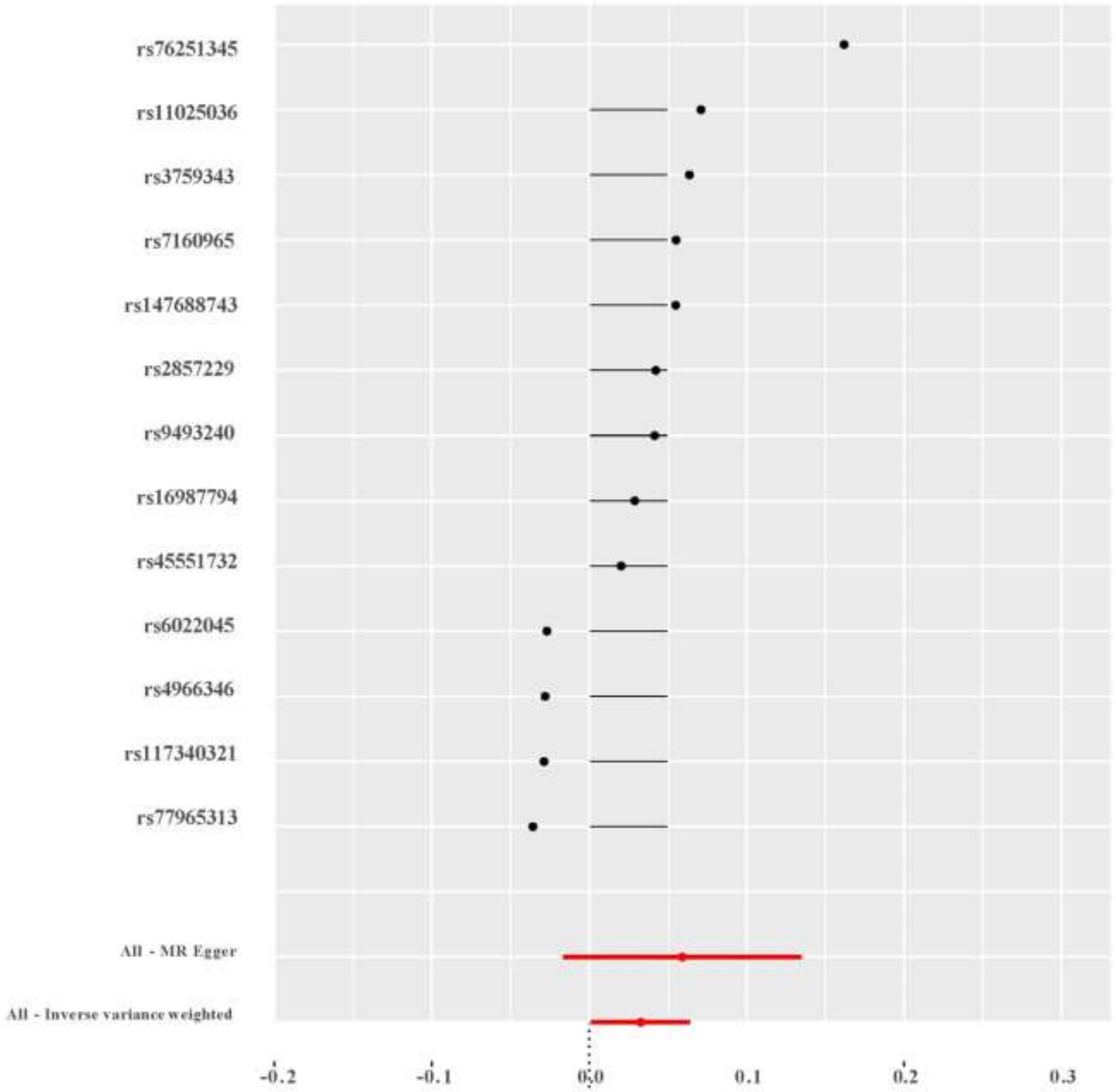

Figure S21: Leave-one-out sensitivity analysis plot of the effect of $CD28^{-}$ $CD127^{-}$ $CD25^{++}$ CD8br %T cell on HF.

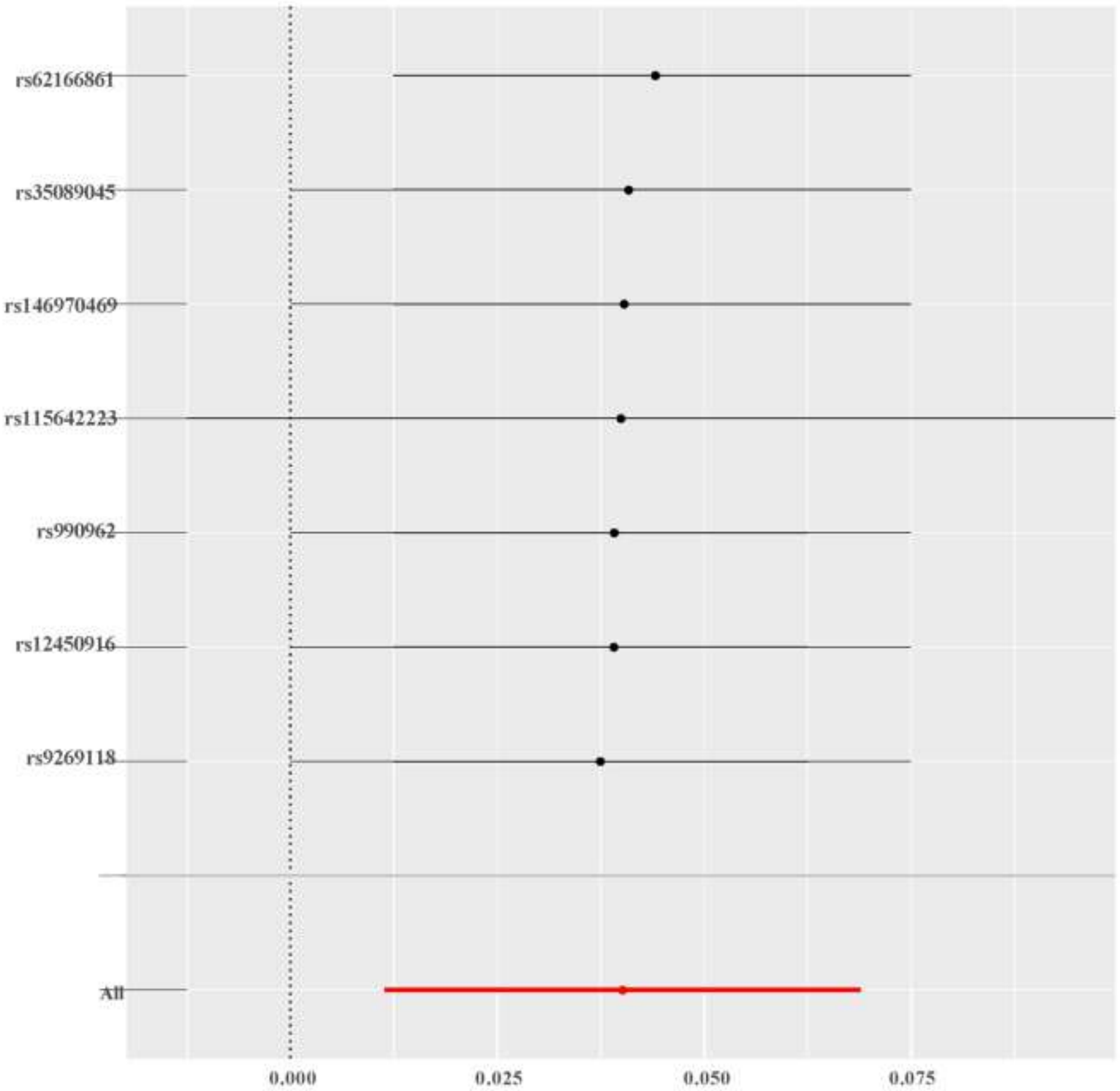

Figure S22: Funnel plot of the effect of CD28⁻ CD127⁻ CD25⁺⁺ CD8br %T cell on HF.

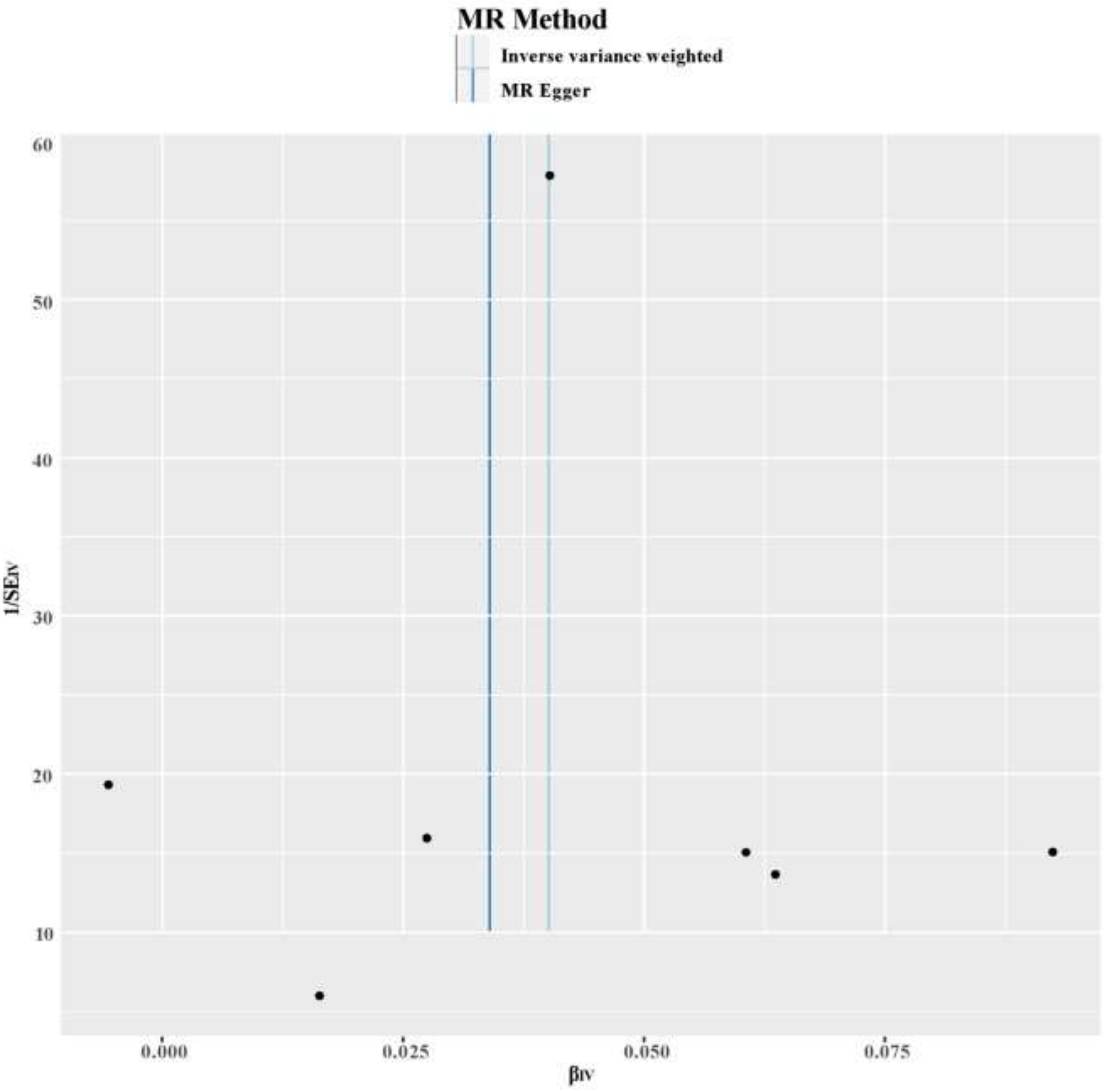

Figure S23: Scatter plot of the effect of $CD28^-$ $CD127^-$ $CD25^{++}$ CD8br %T cell on HF.

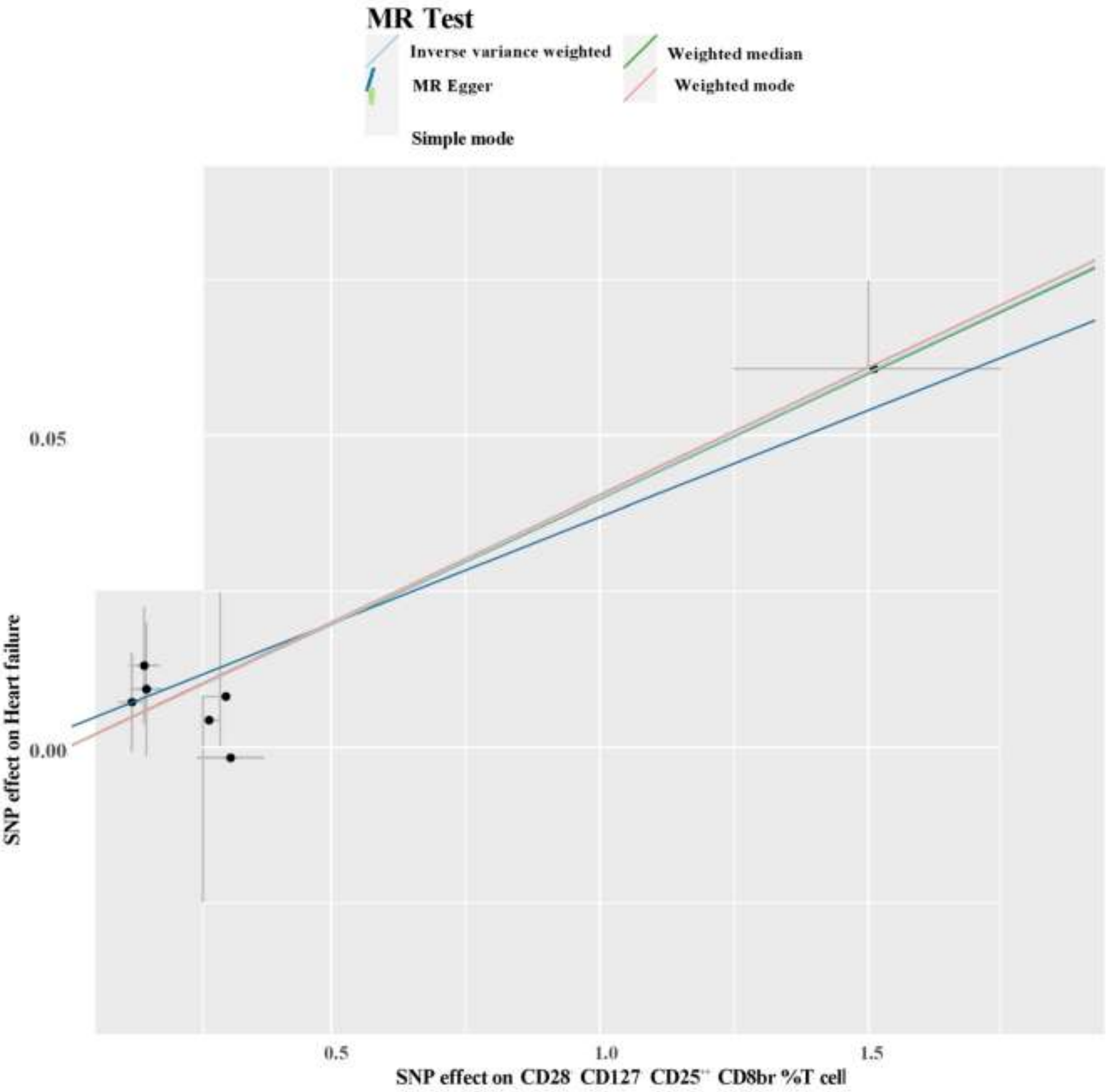

Figure S24: Forest plot of the effect of CD28$^-$ CD127$^-$ CD25^{++} CD8br %T cell on HF.

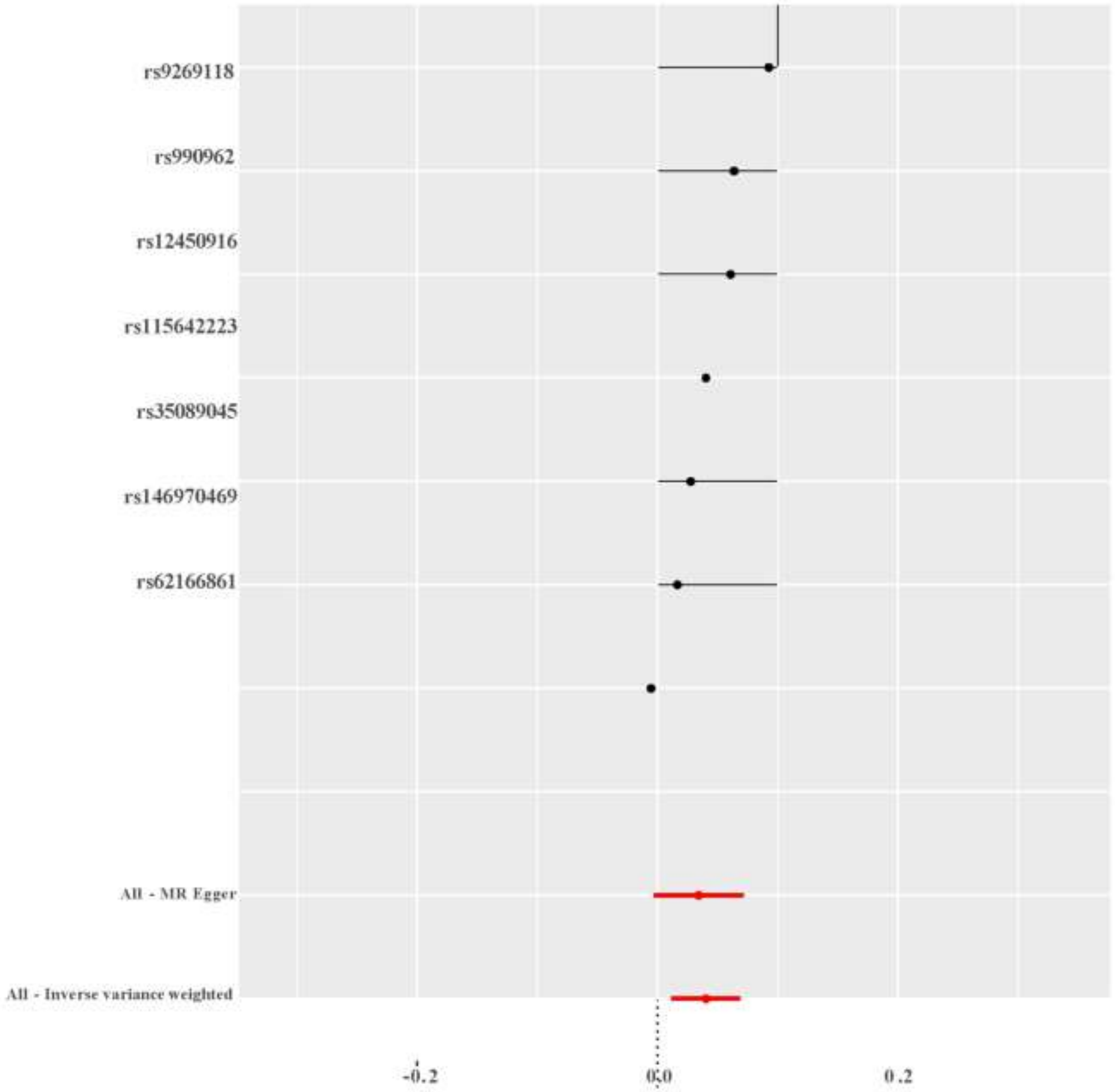

Figure S25: Leave-one-out sensitivity analysis plot of the effect of CD28^{-} CD127^{-} CD25^{++} CD8br %CD8br on HF.

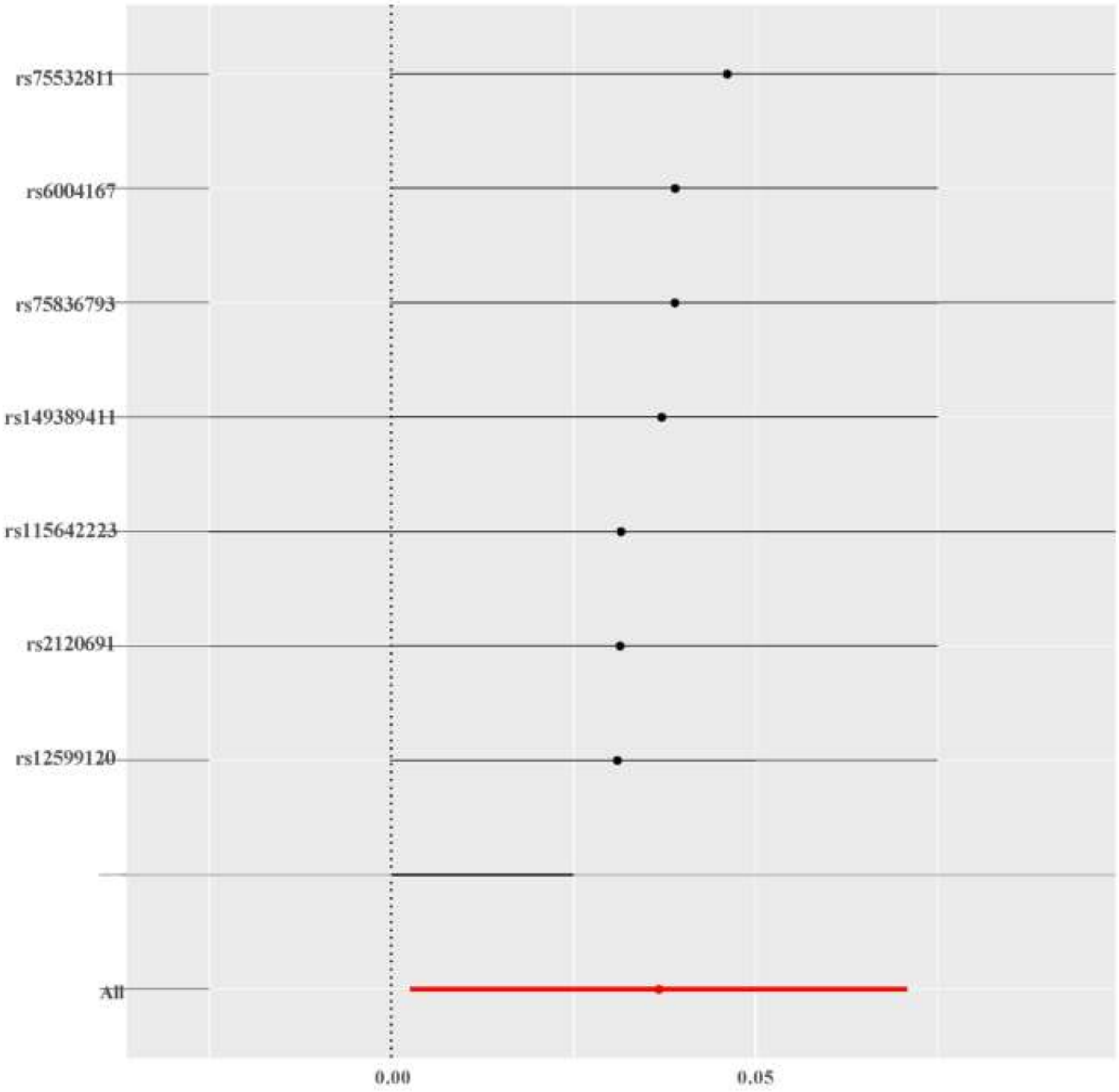

Figure S26: Funnel plot of the effect of CD28⁻ CD127⁻ CD25⁺⁺ CD8br %CD8br on HF.

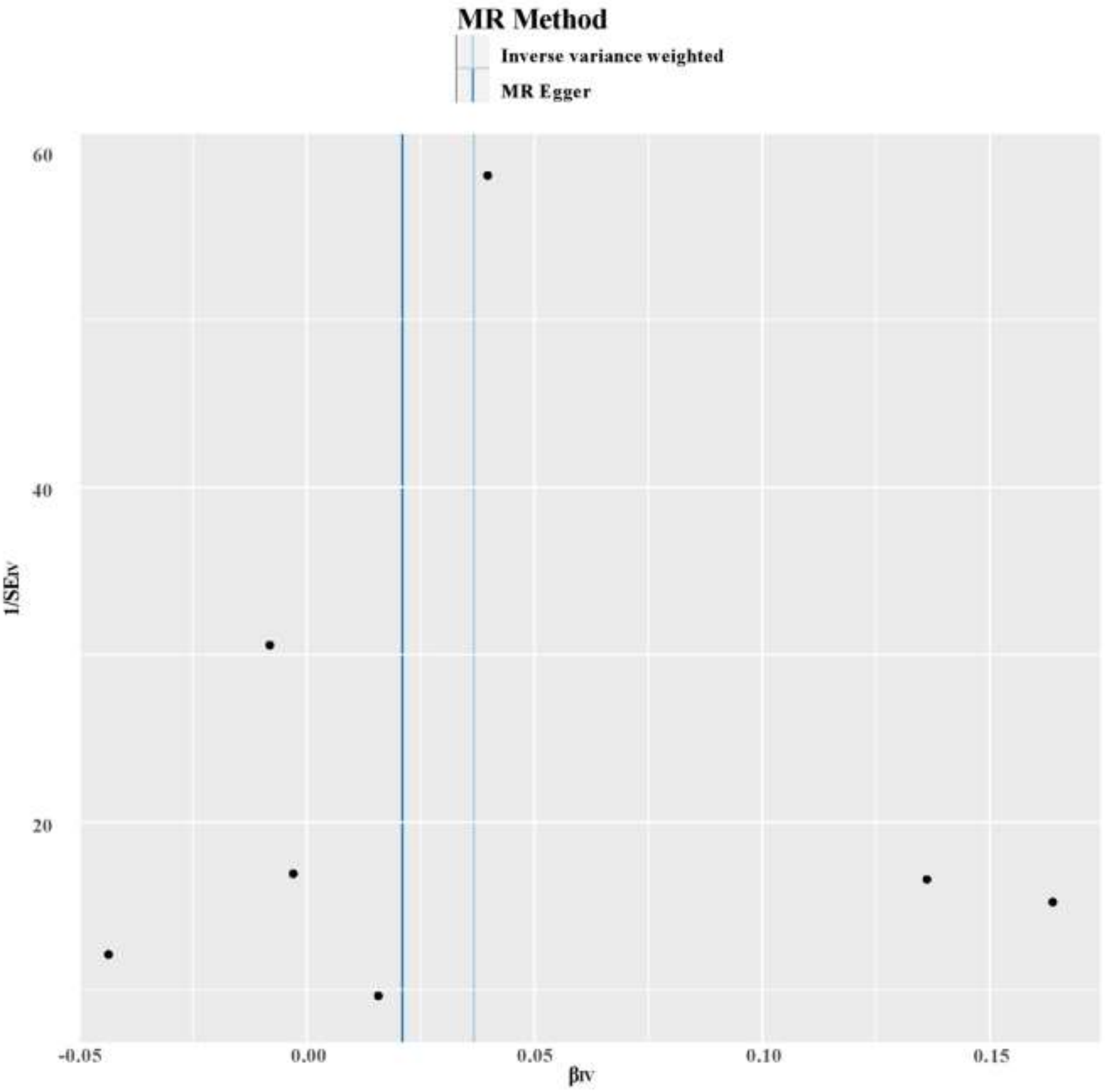

Figure S27: Scatter plot of the effect of CD28^{-} CD127^{-} CD25^{++} CD8br %CD8br on HF.

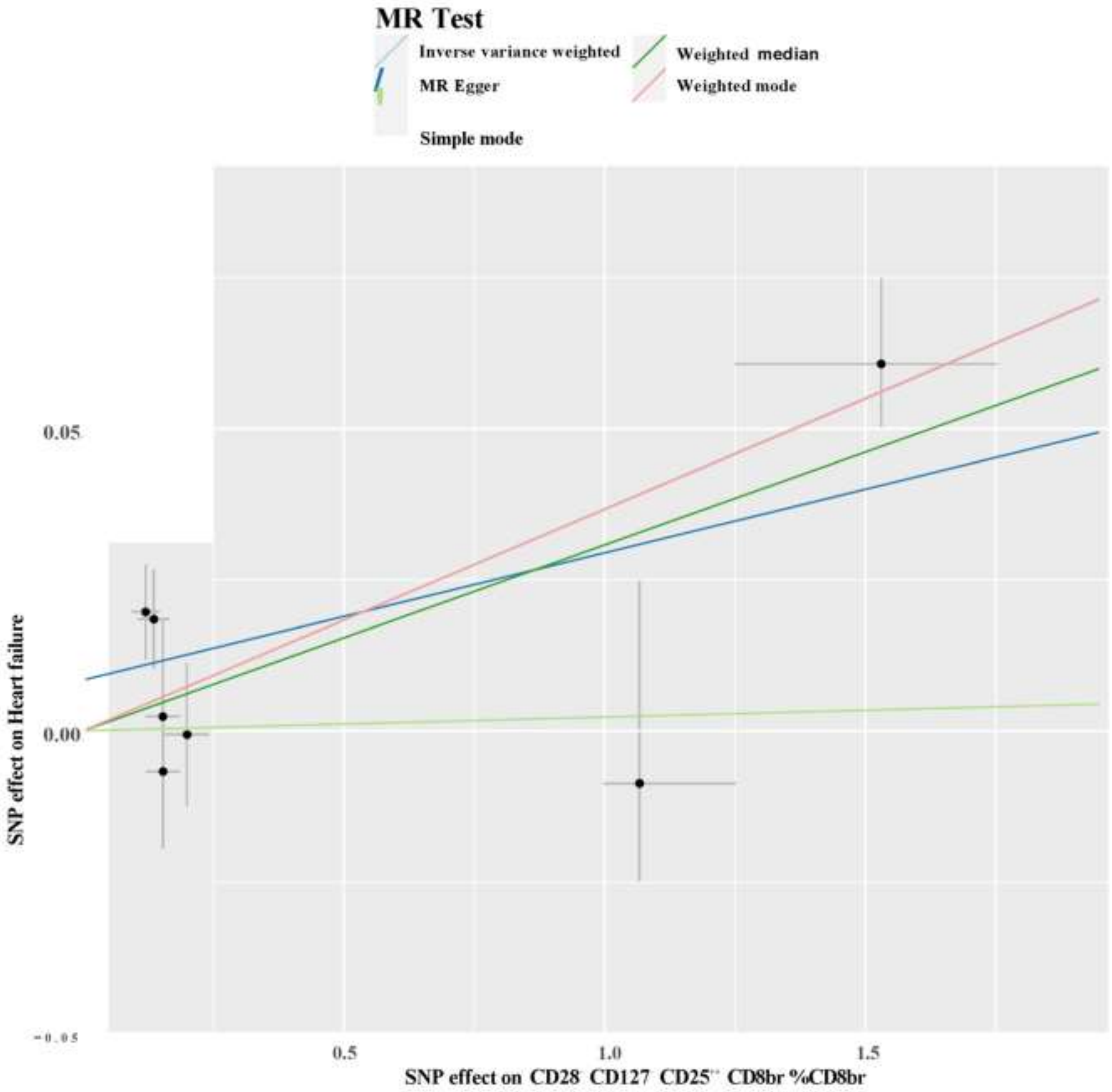

Figure S28: Forest plot of the effect of CD28⁻ CD127⁻ CD25⁺⁺ CD8br %CD8br on HF.

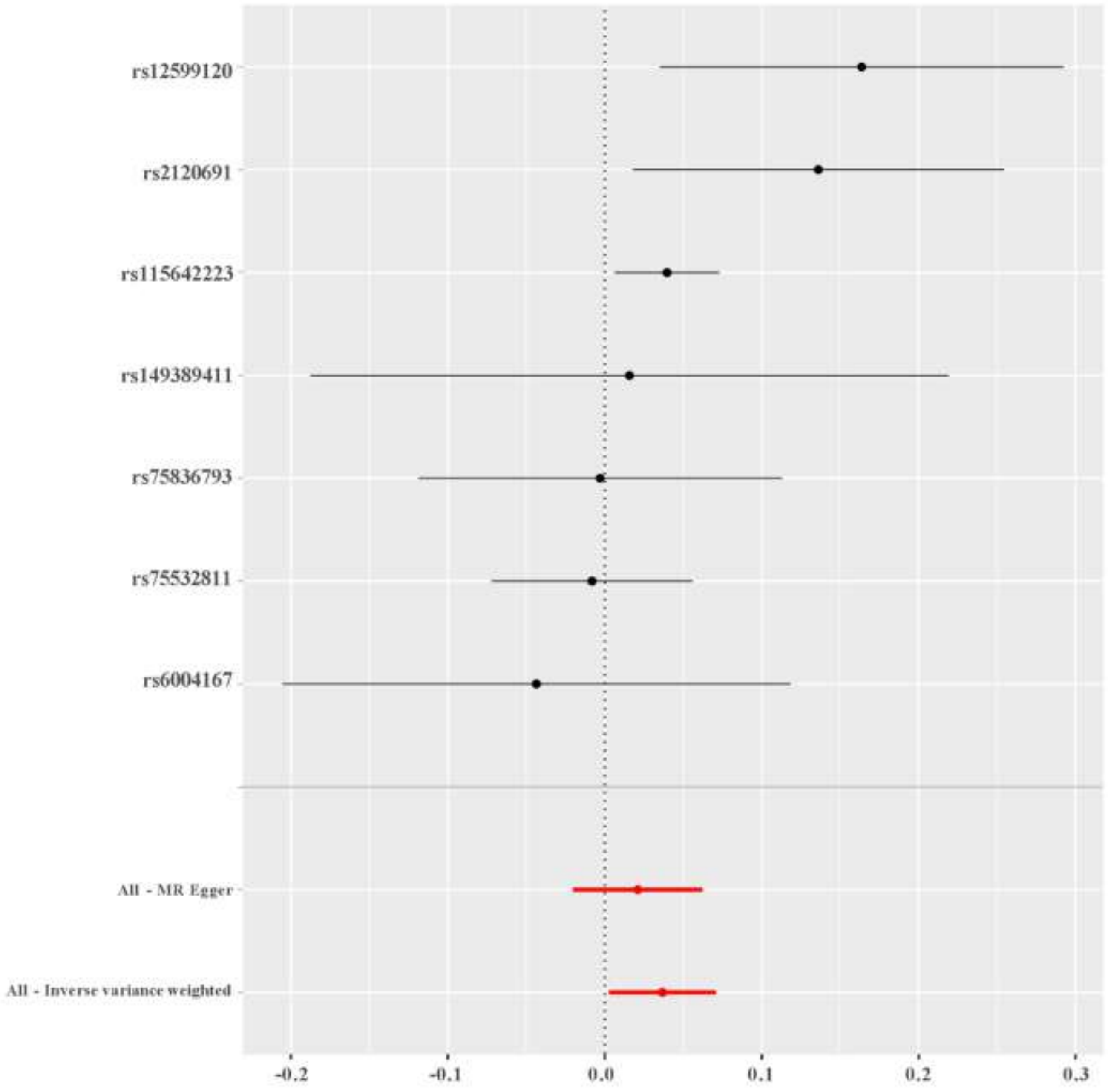

Figure S29: Leave-one-out sensitivity analysis plot of the effect of CD28^{+} CD45RA^{+} CD8br %T cell on HF.

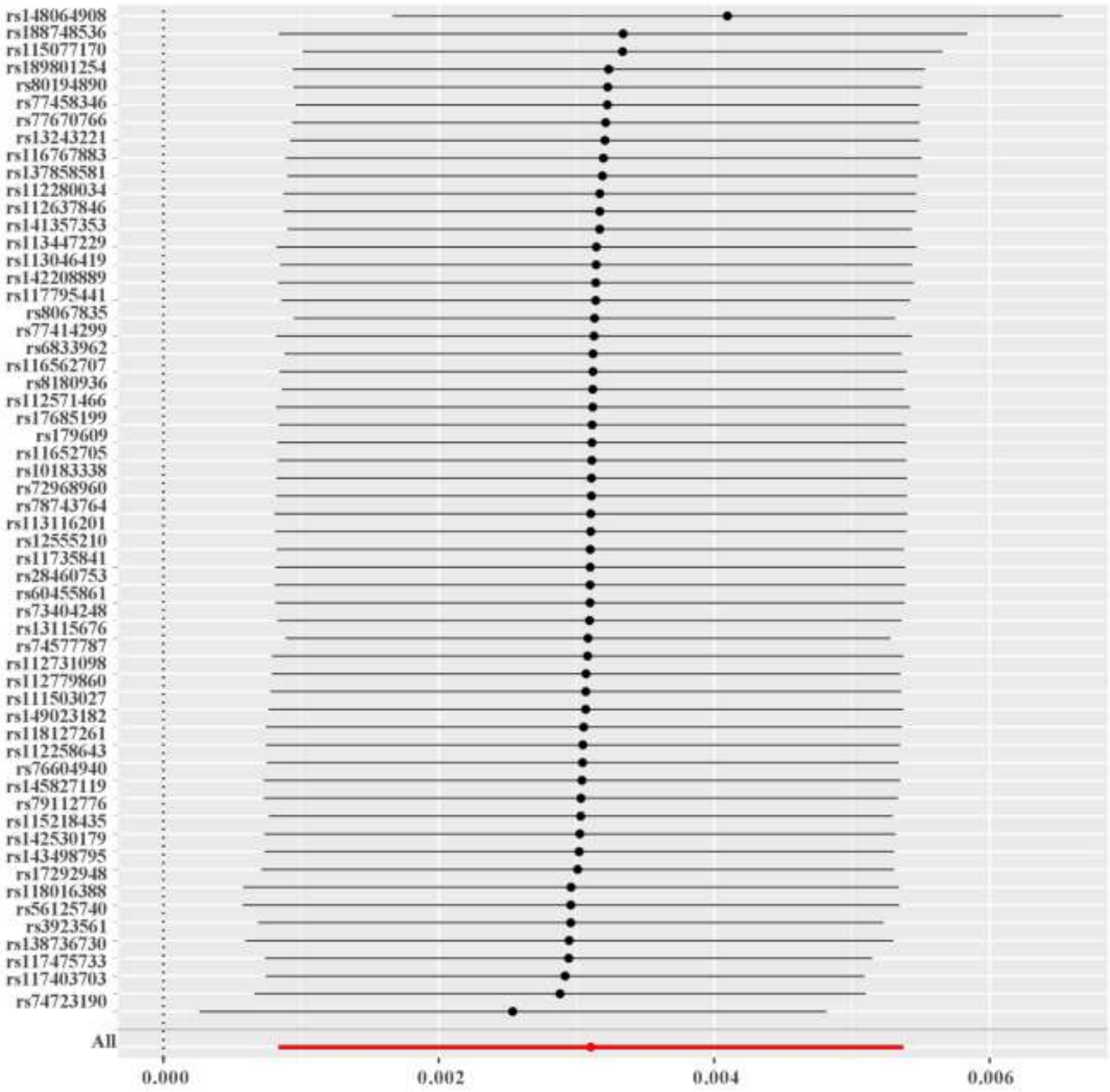

Figure S30: Funnel plot of the effect of CD28$^+$ CD45RA$^+$ CD8br %T cell on HF.

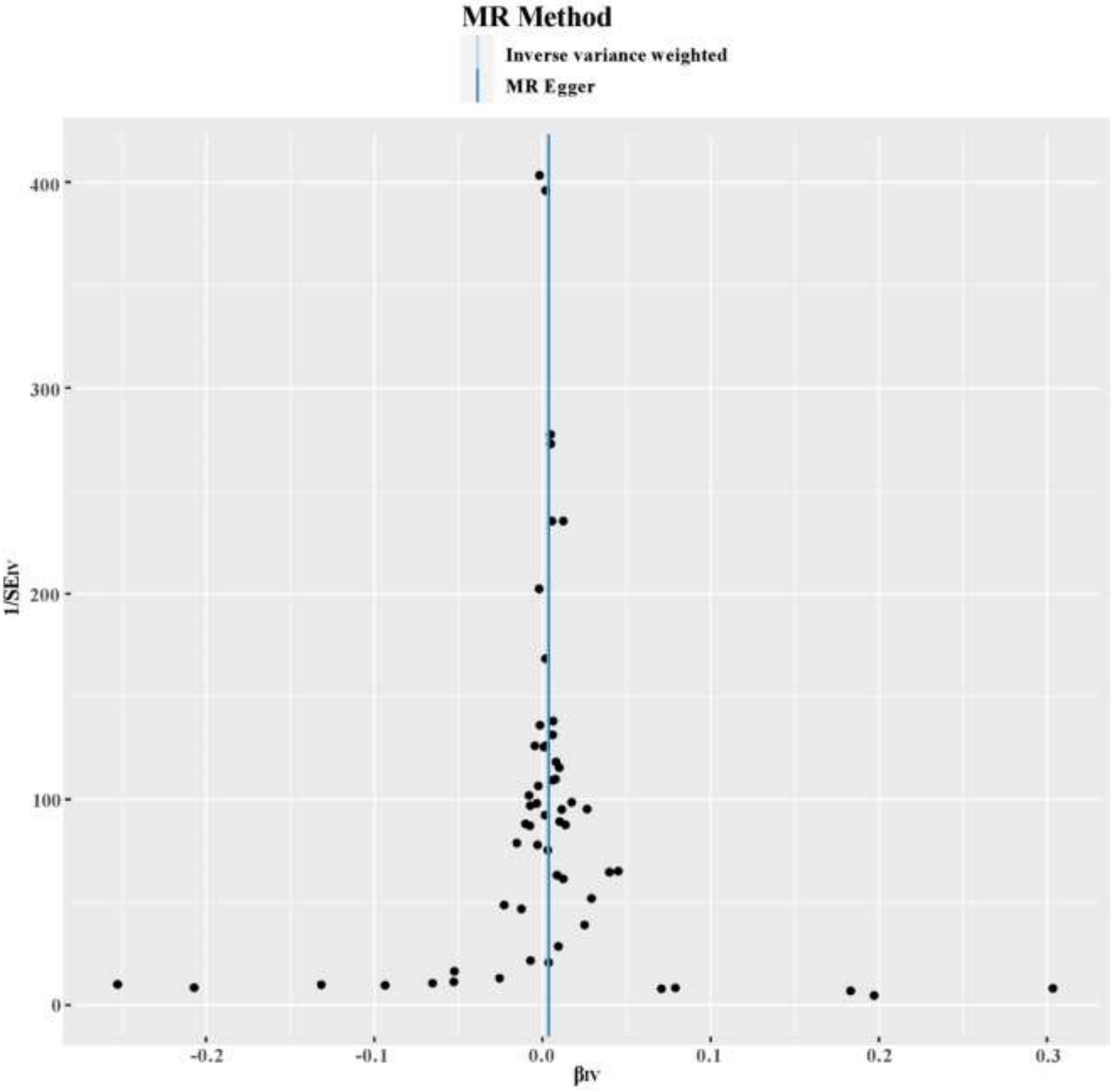

Figure S31: Scatter plot of the effect of CD28^{+} CD45RA^{+} CD8br %T cell on HF.

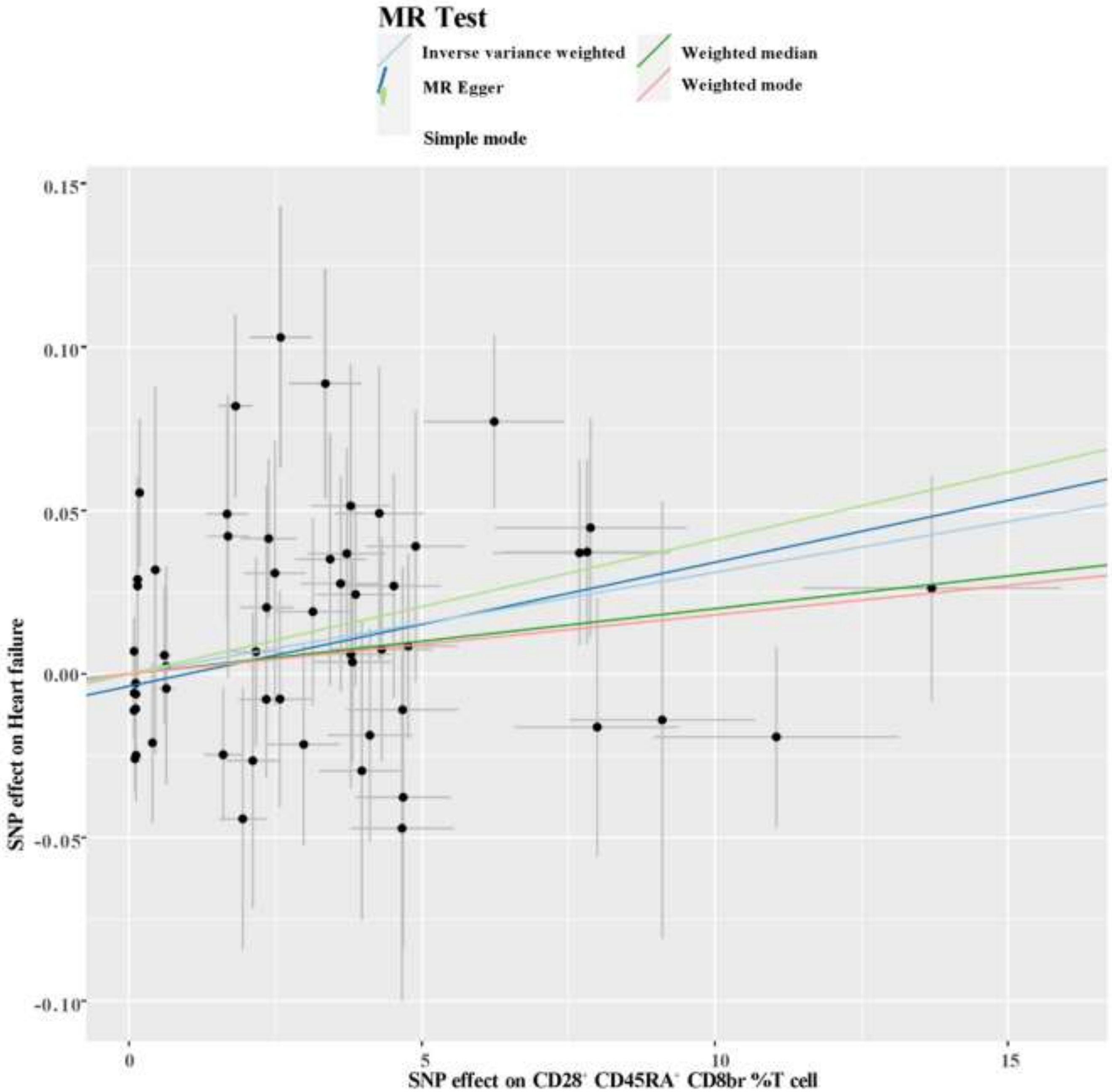

Figure S32: Forest plot of the effect of CD28^{+} CD45RA^{+} CD8br %T cell on HF.

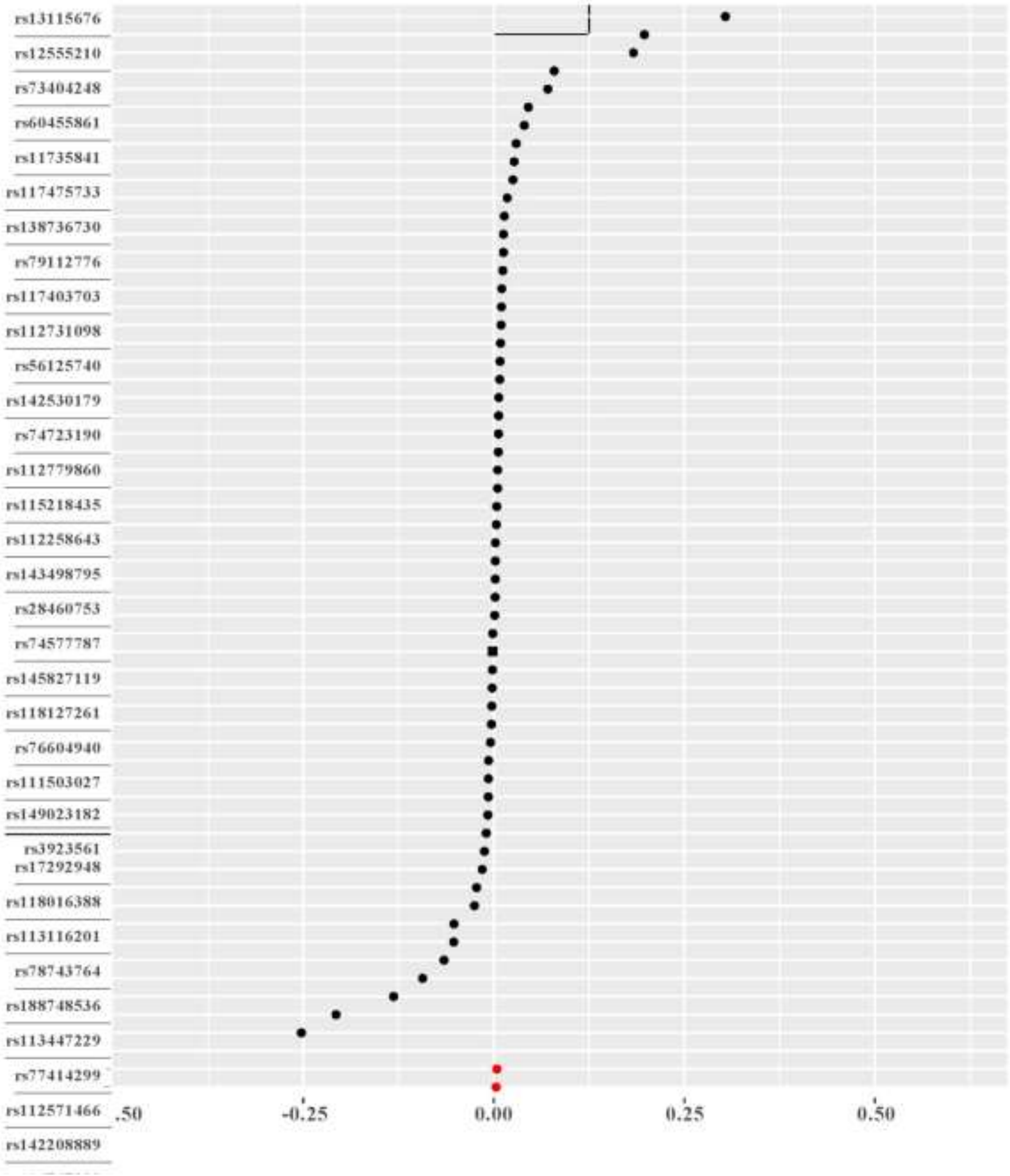

Figure S33: Leave-one-out sensitivity analysis plot of the effect of CD19 on IgD^{+} CD38br on HF.

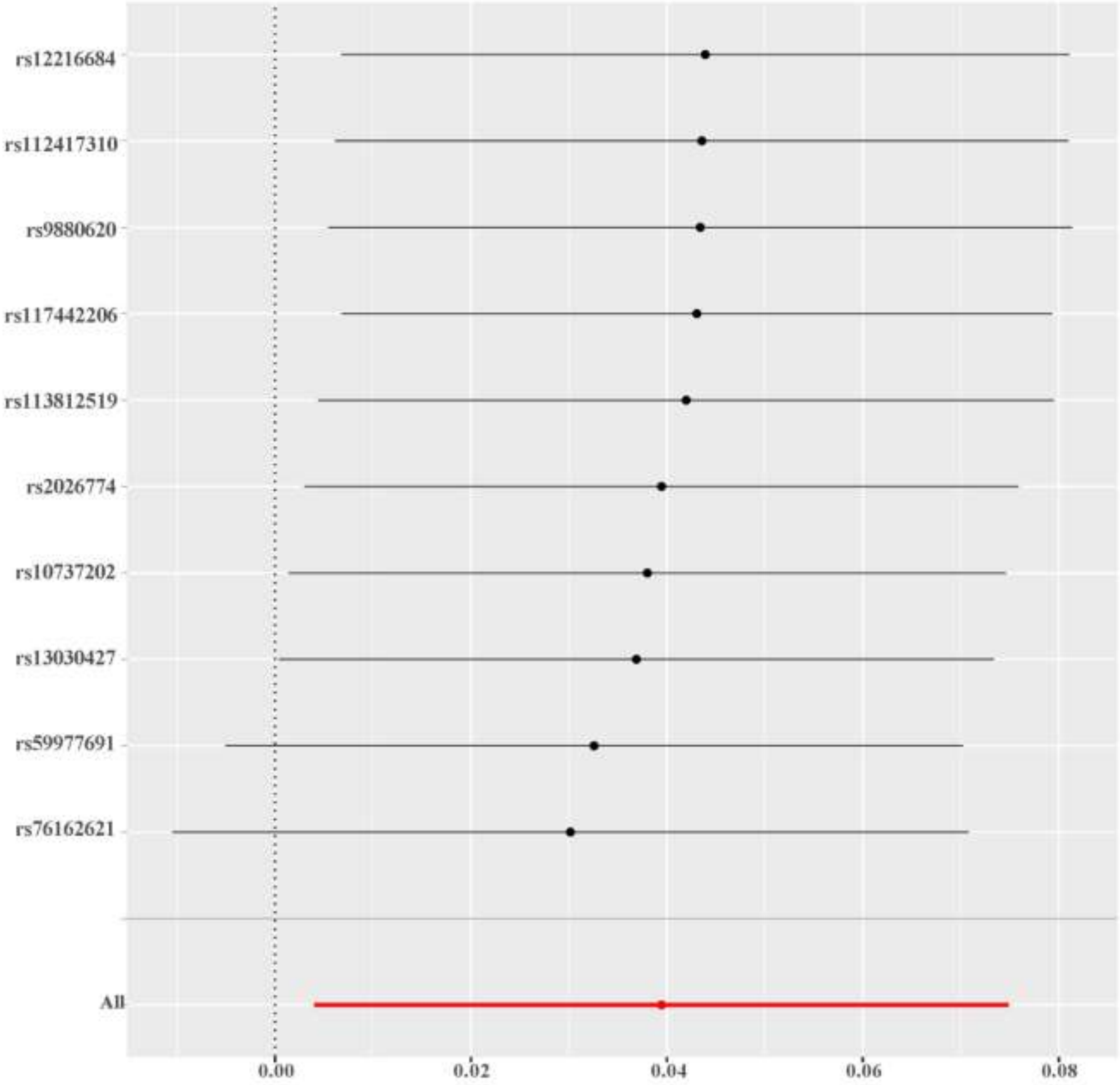

Figure S34: Funnel plot of the effect of CD19 on IgD$^+$ CD38br on HF.

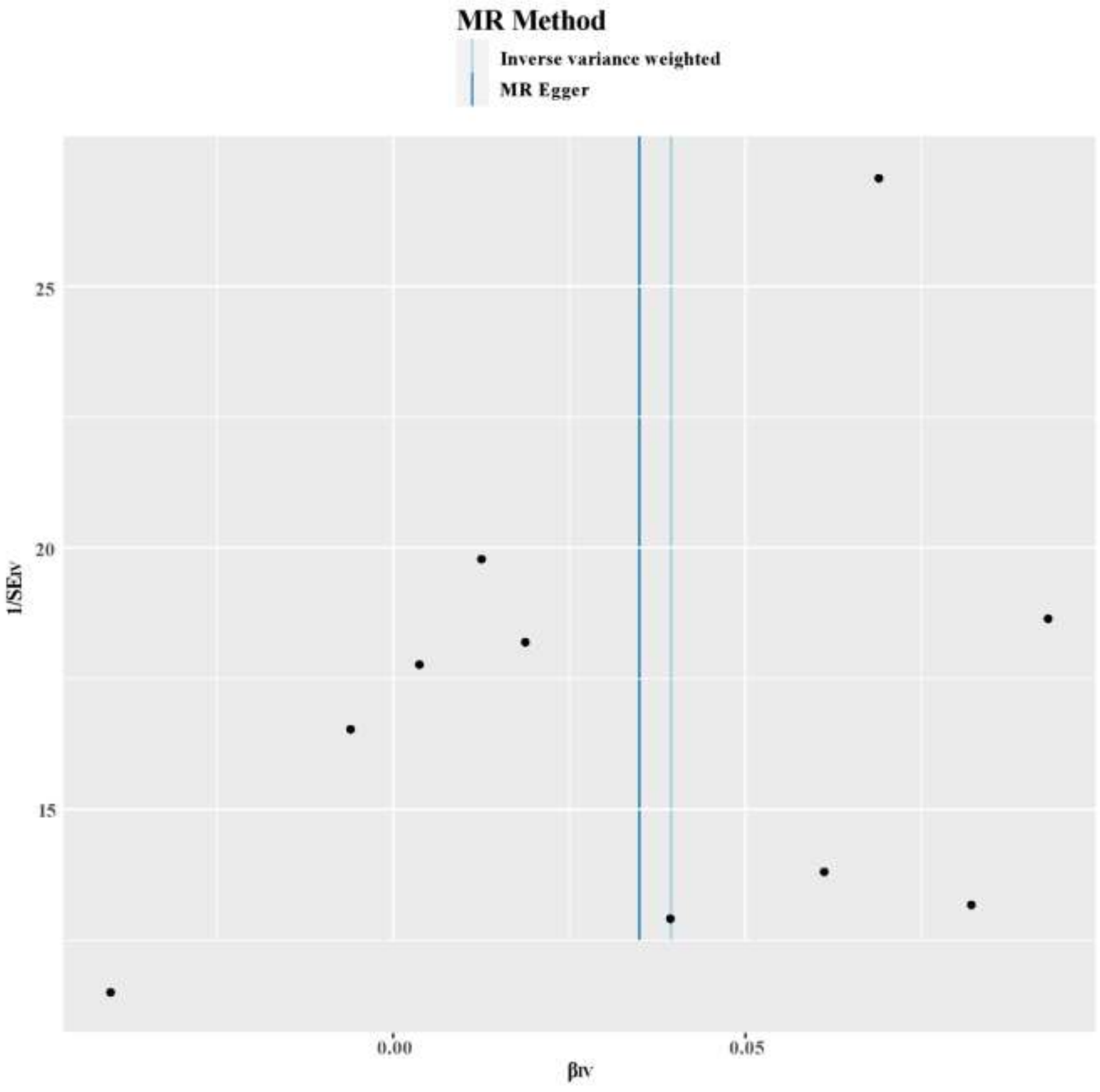

Figure S35: Scatter plot of the effect of CD19 on IgD+ CD38br on HF.

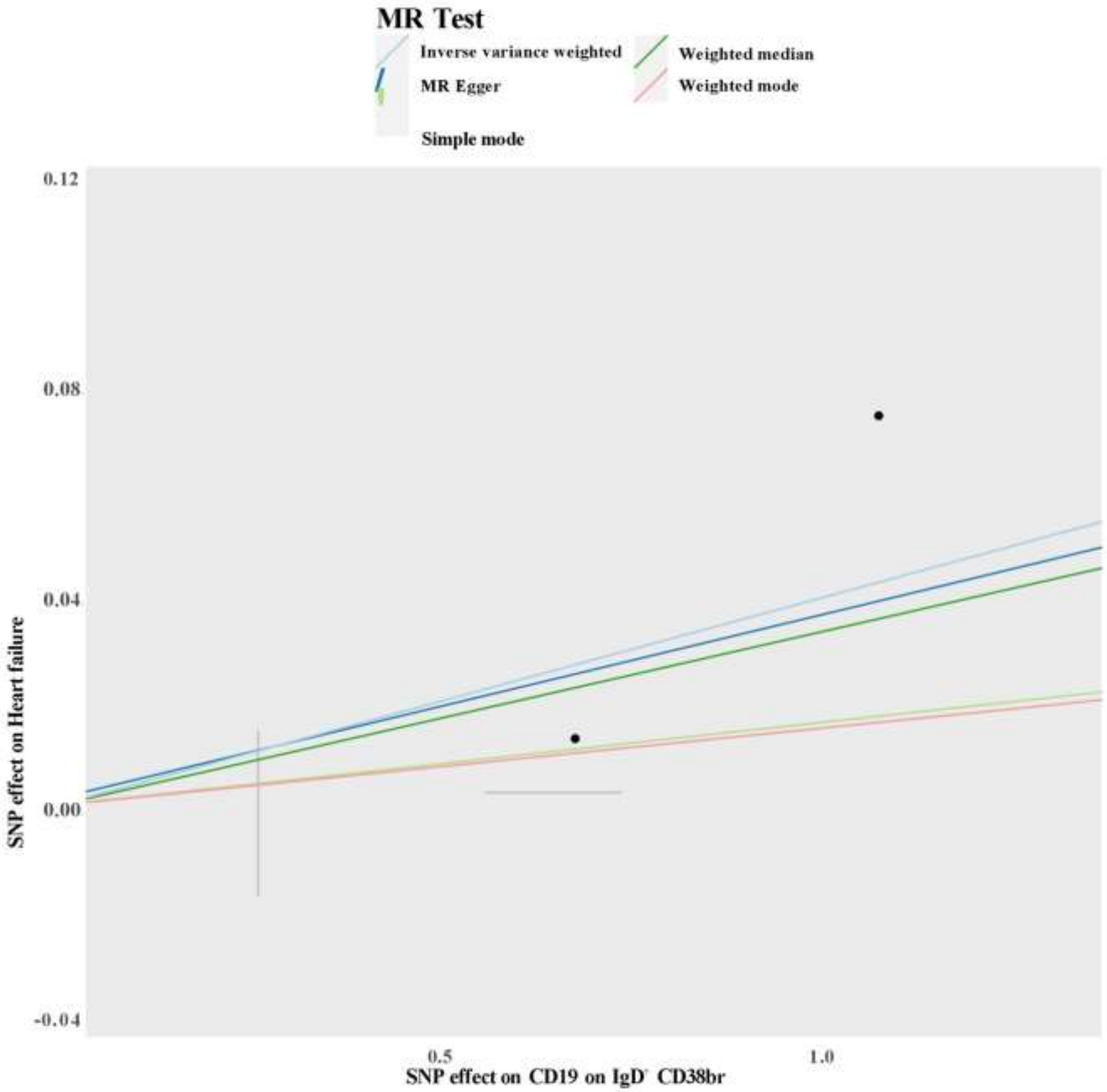

Figure S36: Forest plot of the effect of CD19 on IgD$^+$ CD38br on HF.

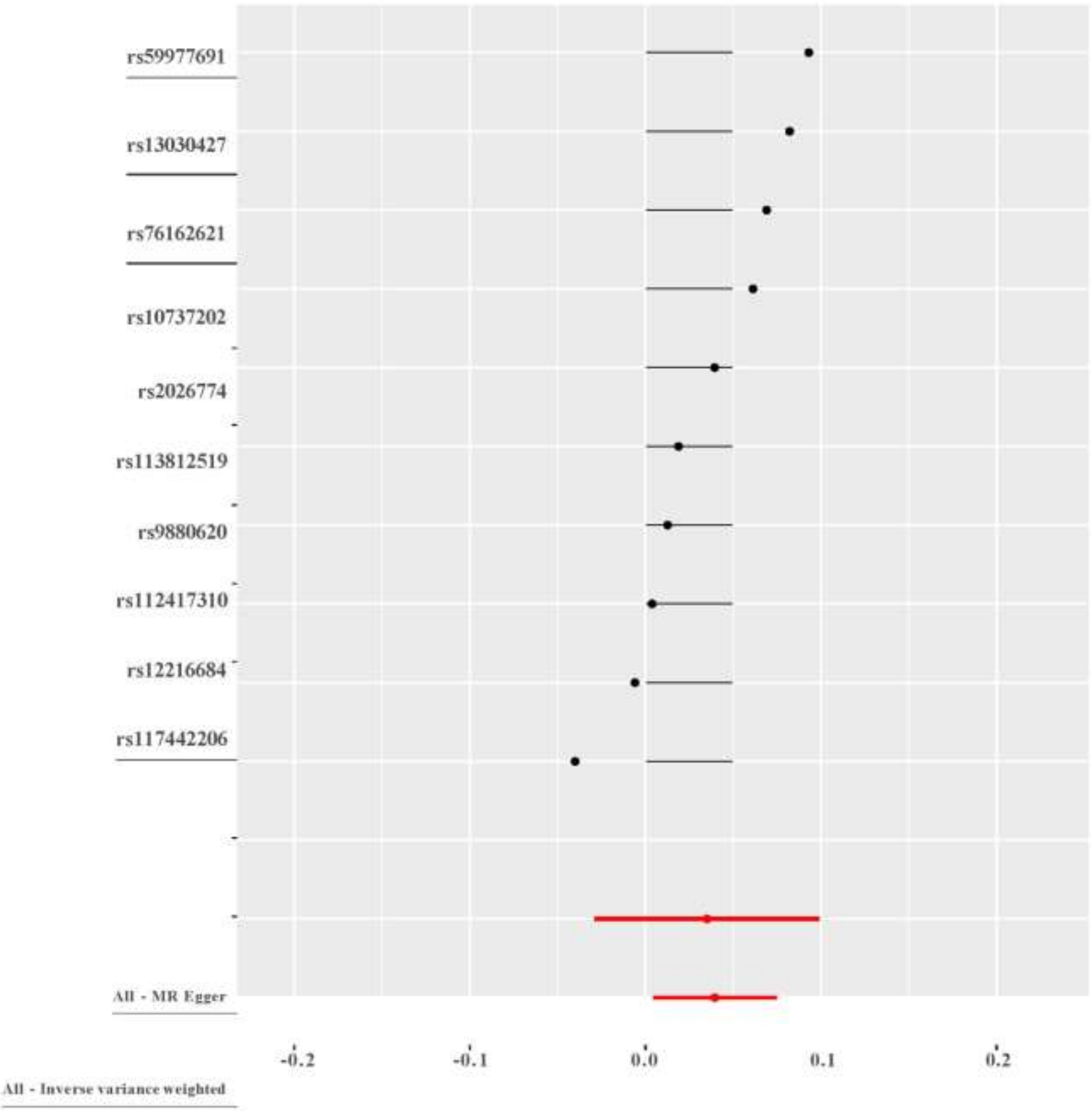

Figure S37: Leave-one-out sensitivity analysis plot of the effect of CD20 on IgD^{-} CD38^{dim} on HF.

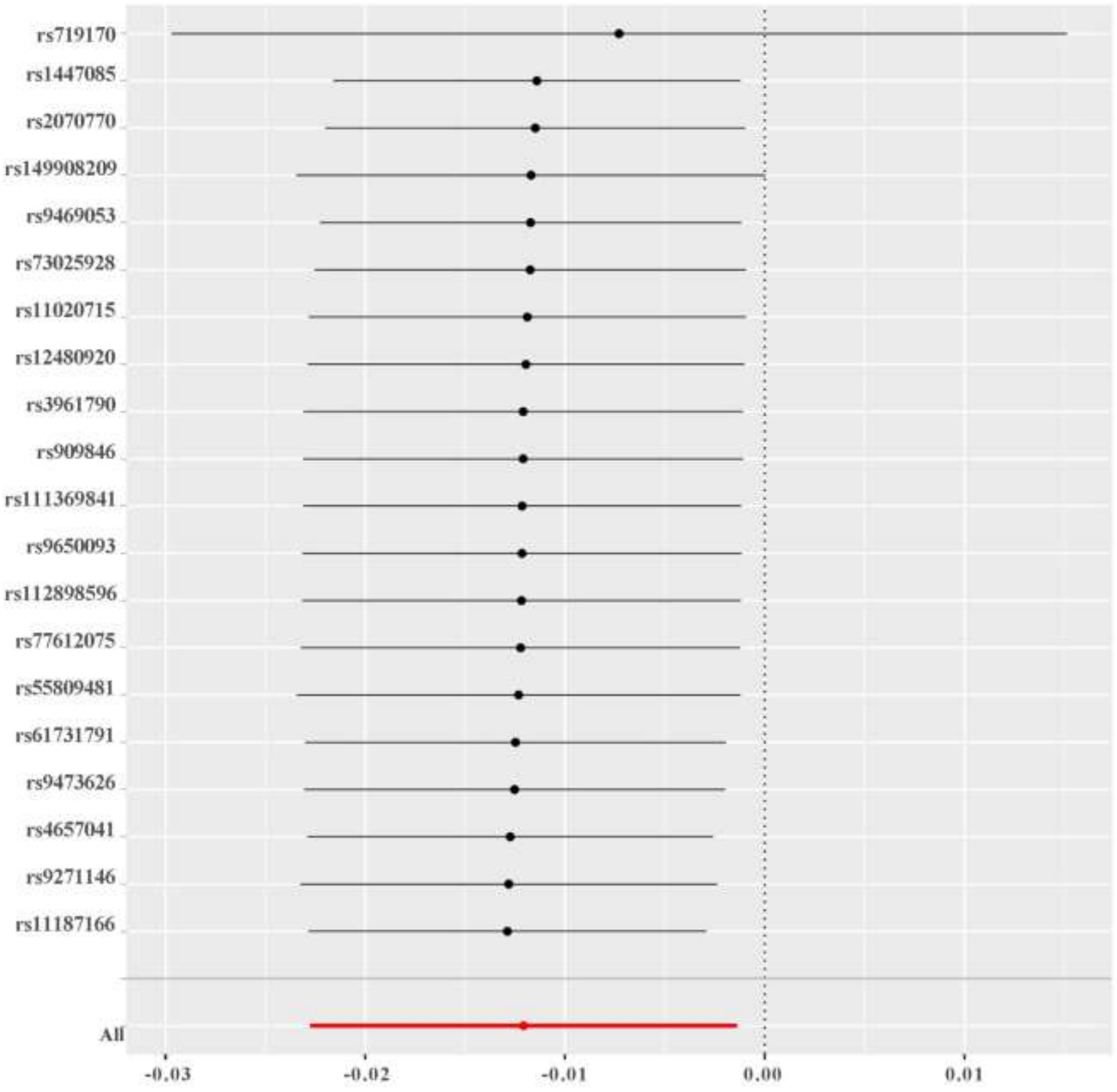

Figure S38: Funnel plot of the effect of CD20 on IgD$^-$ CD38^{dim} on HF.

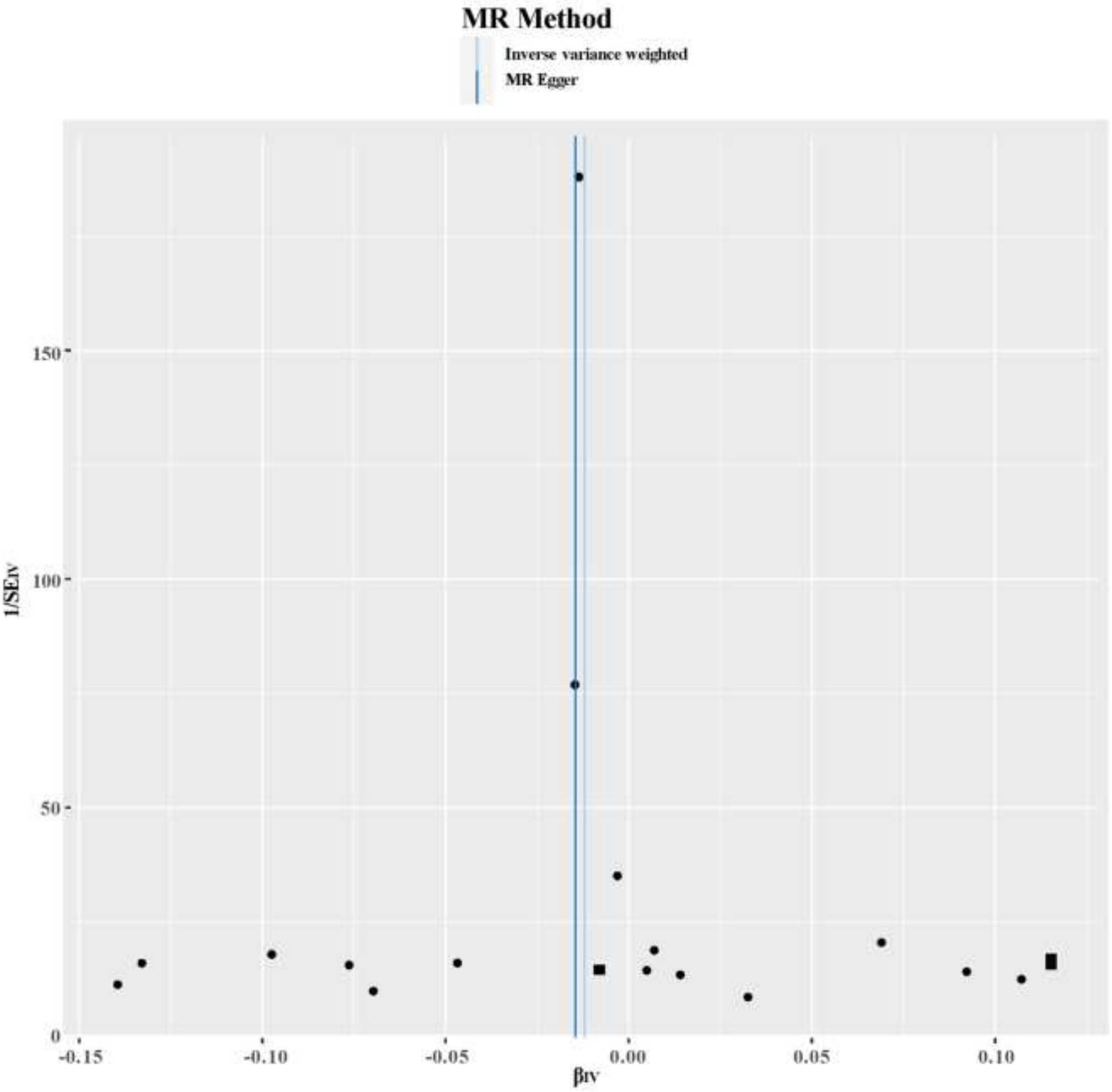

Figure S39: Scatter plot of the effect of CD20 on IgD$^-$ CD38^{dim} on HF.

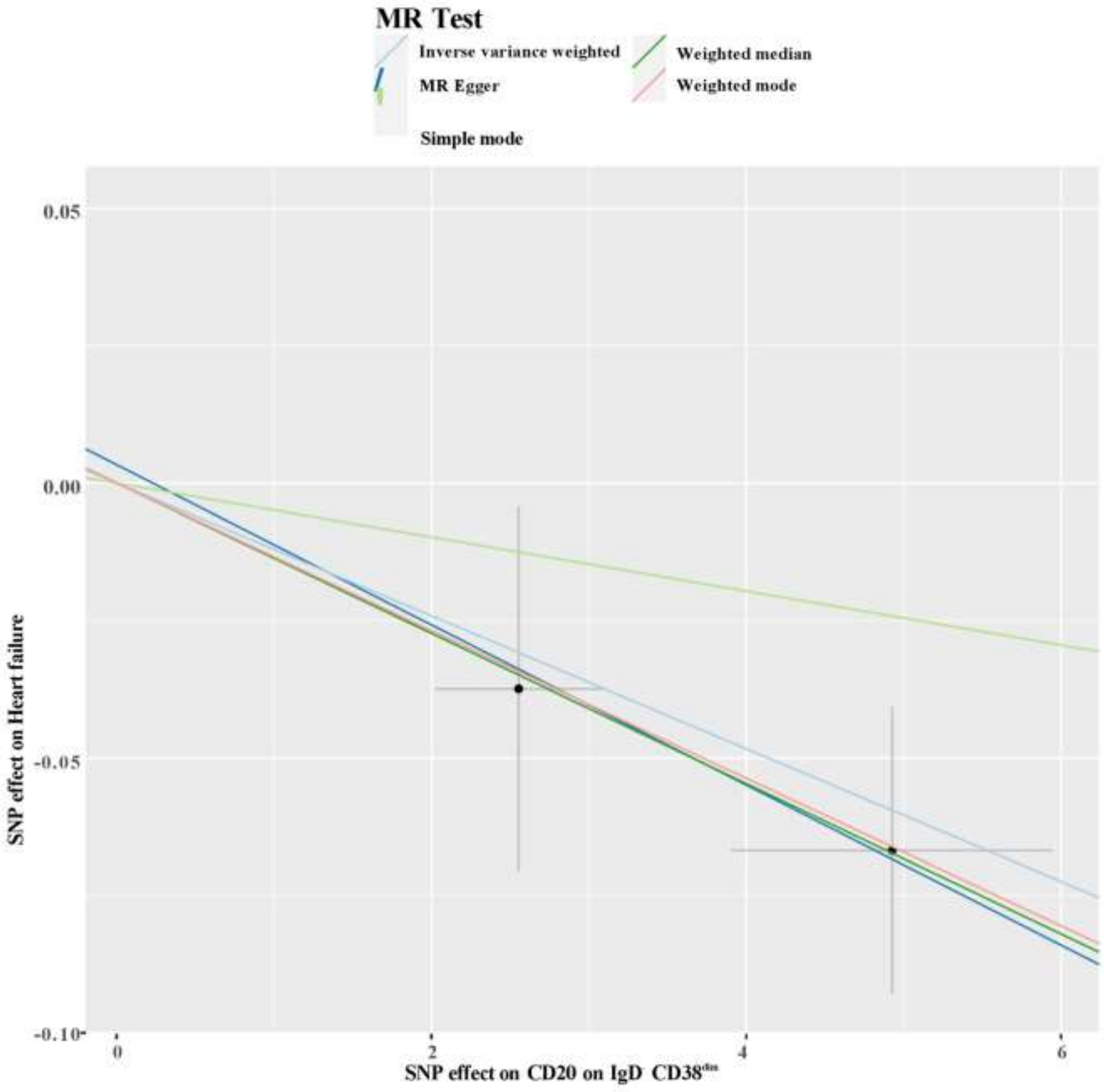

Figure S40: Forest plot of the effect of CD20 on IgD$^-$ CD38^{dim} on HF.

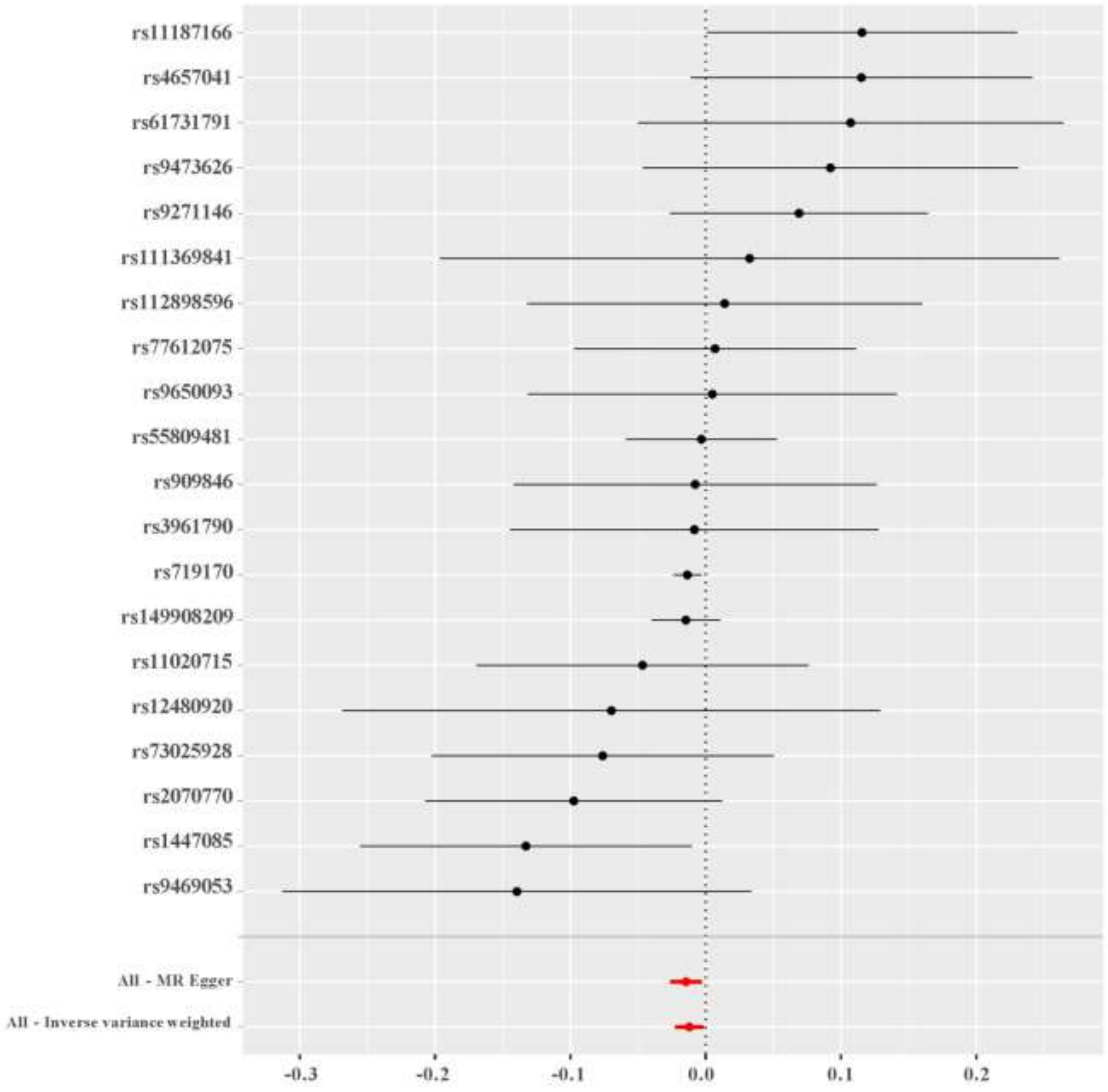

Figure S41: Leave-one-out sensitivity analysis plot of the effect of CD27 on IgD$^-$ CD38^{dim} on HF.

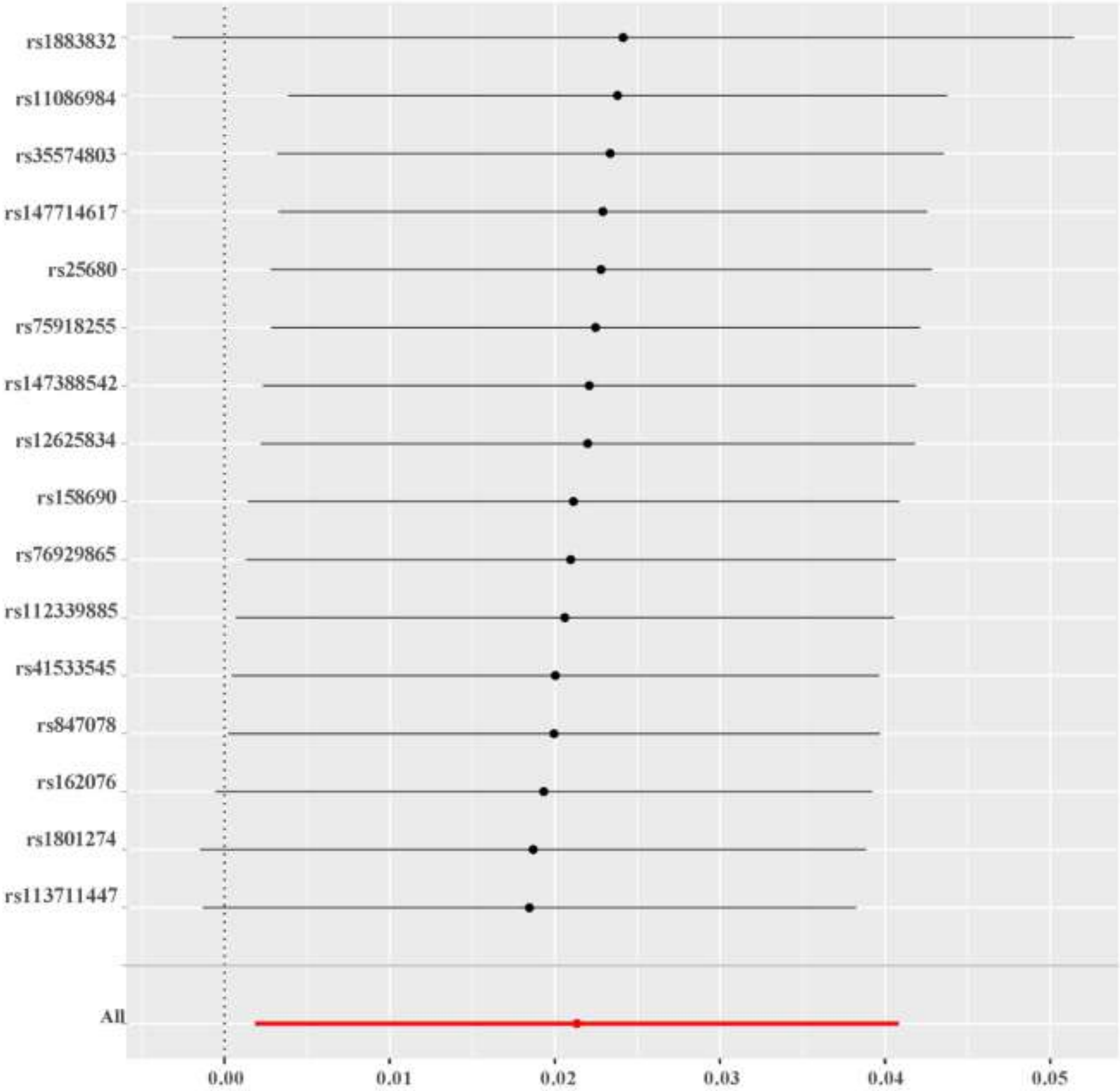

Figure S42: Funnel plot of the effect of CD27 on IgD$^-$ CD38^{dim} on HF.

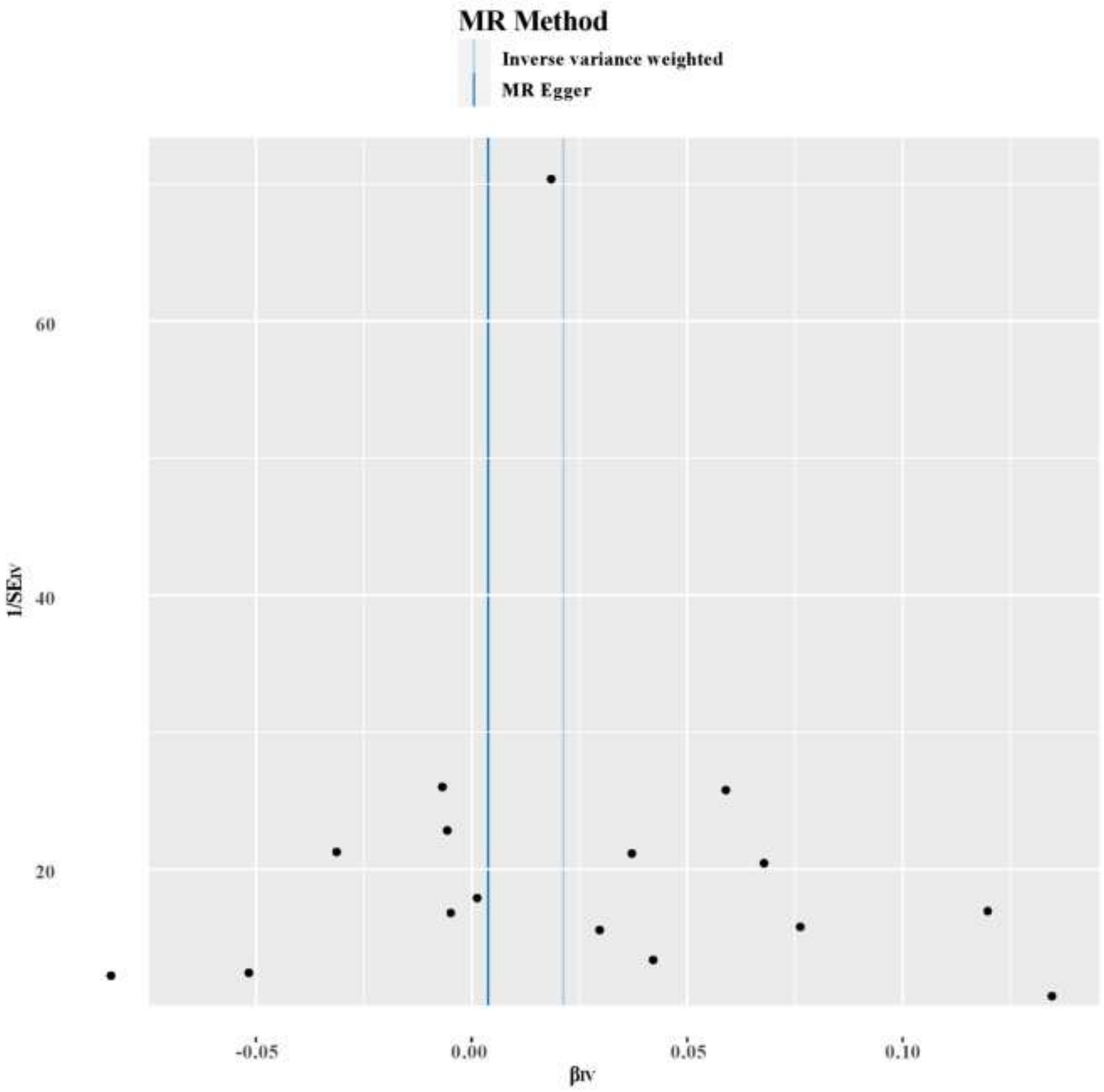

Figure S43: Scatter plot of the effect of CD27 on IgD- CD38dim on HF.

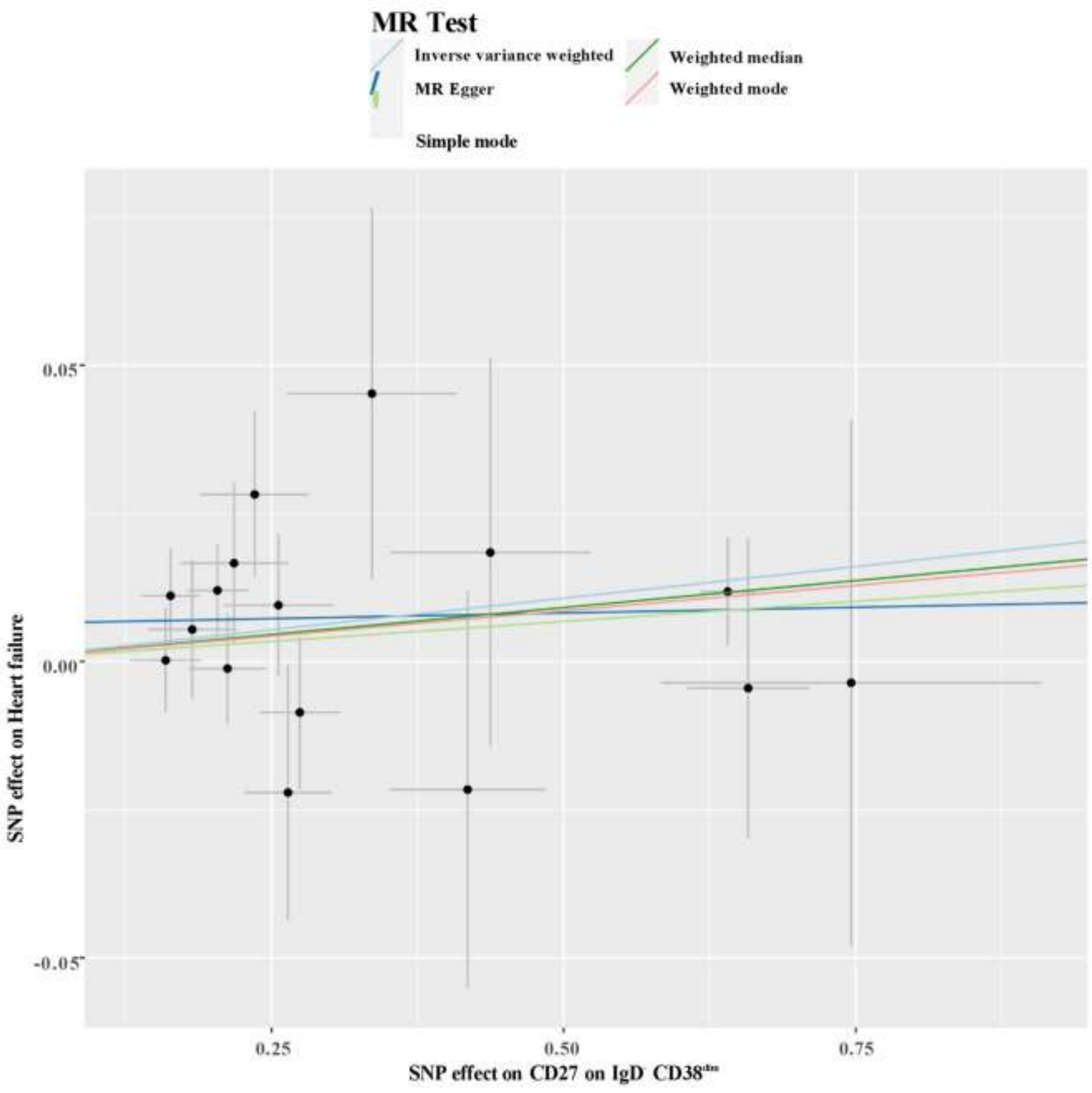

Figure S44: Forest plot of the effect of CD27 on IgD- CD38dim on HF.

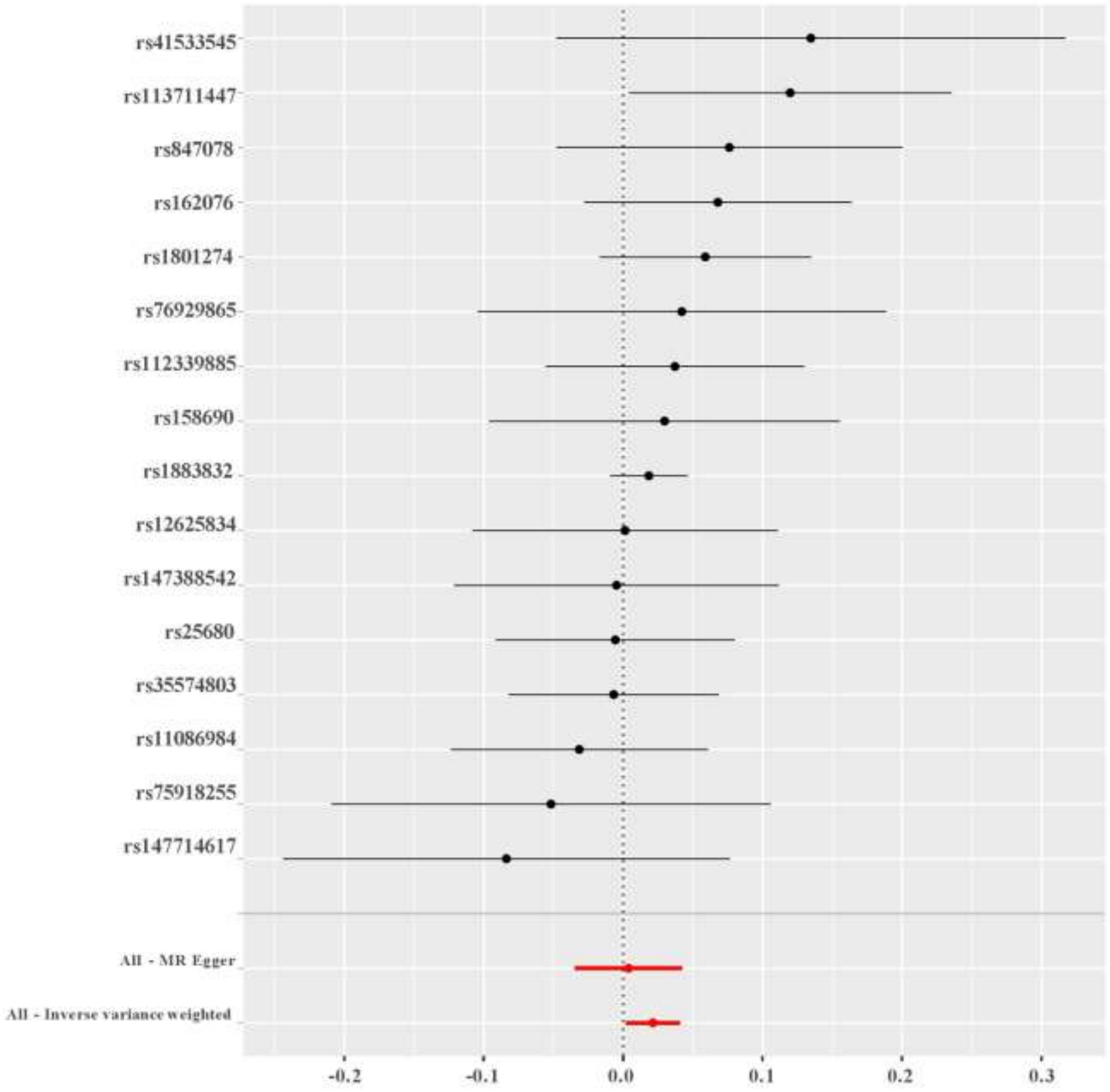

Figure S45: Leave-one-out sensitivity analysis plot of the effect of CD45 on lymphocyte on HF.

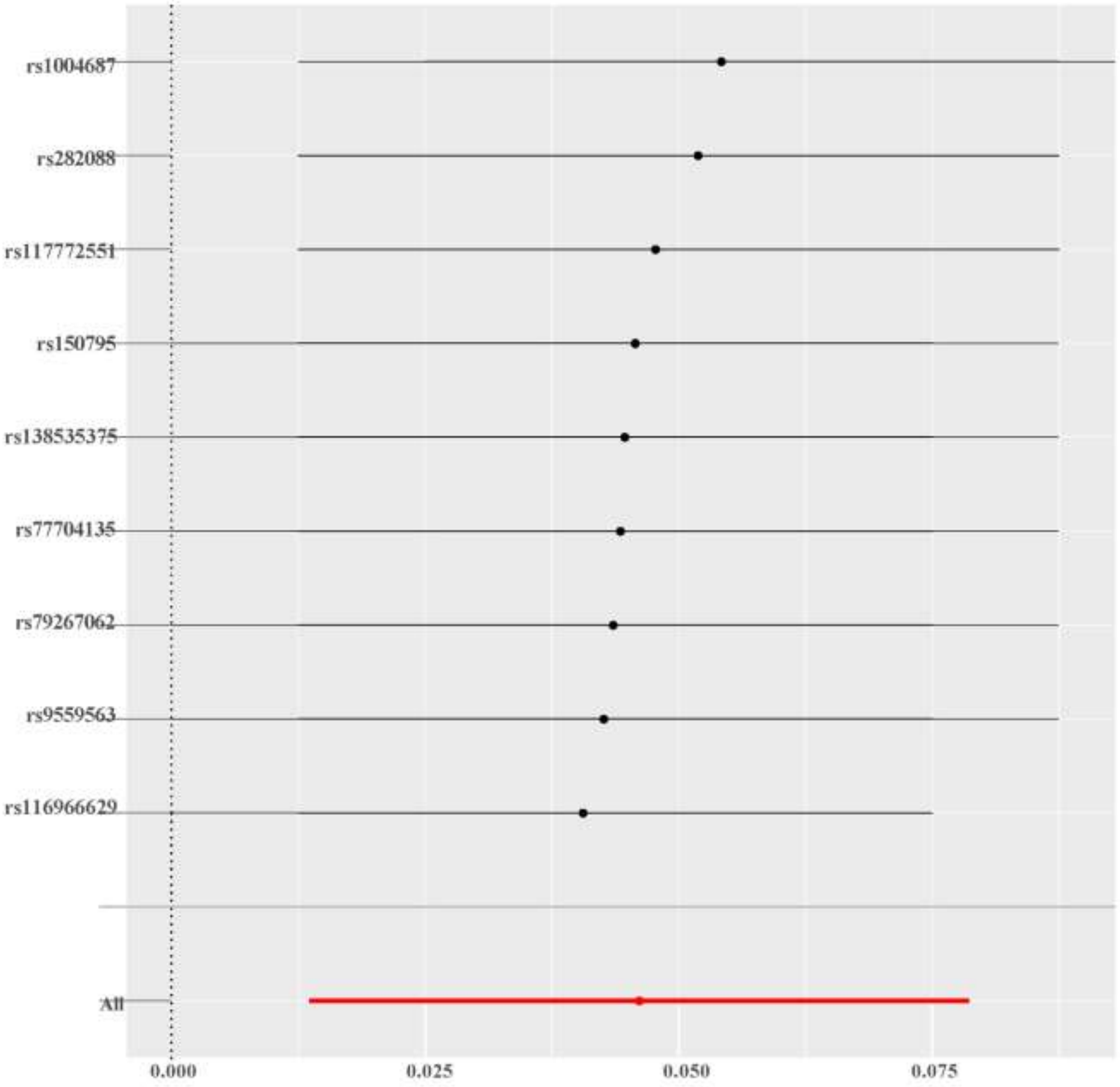

Figure S46: Funnel plot of the effect of CD45 on lymphocyte on HF.

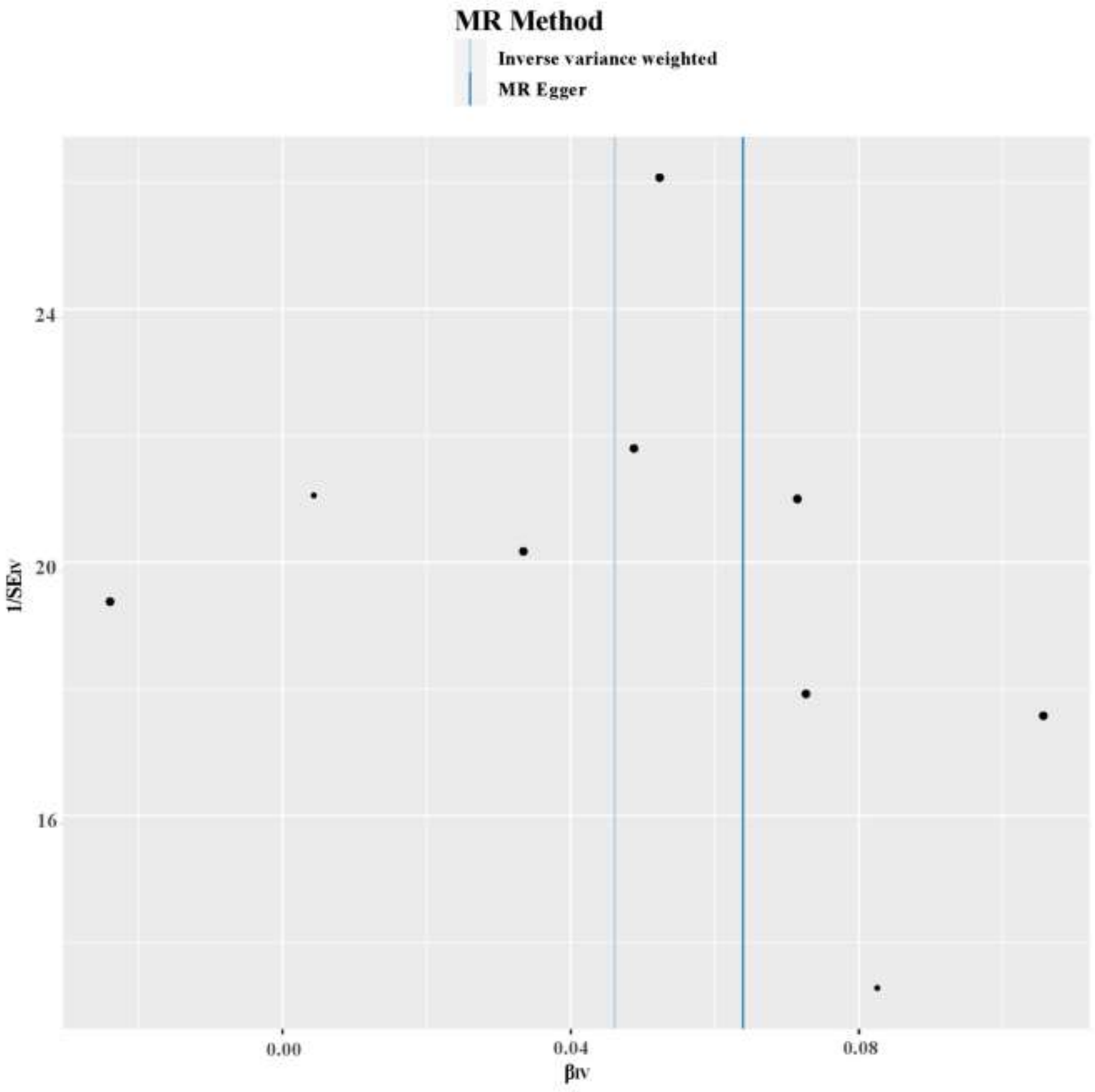

Figure S47: Scatter plot of the effect of CD45 on lymphocyte on HF.

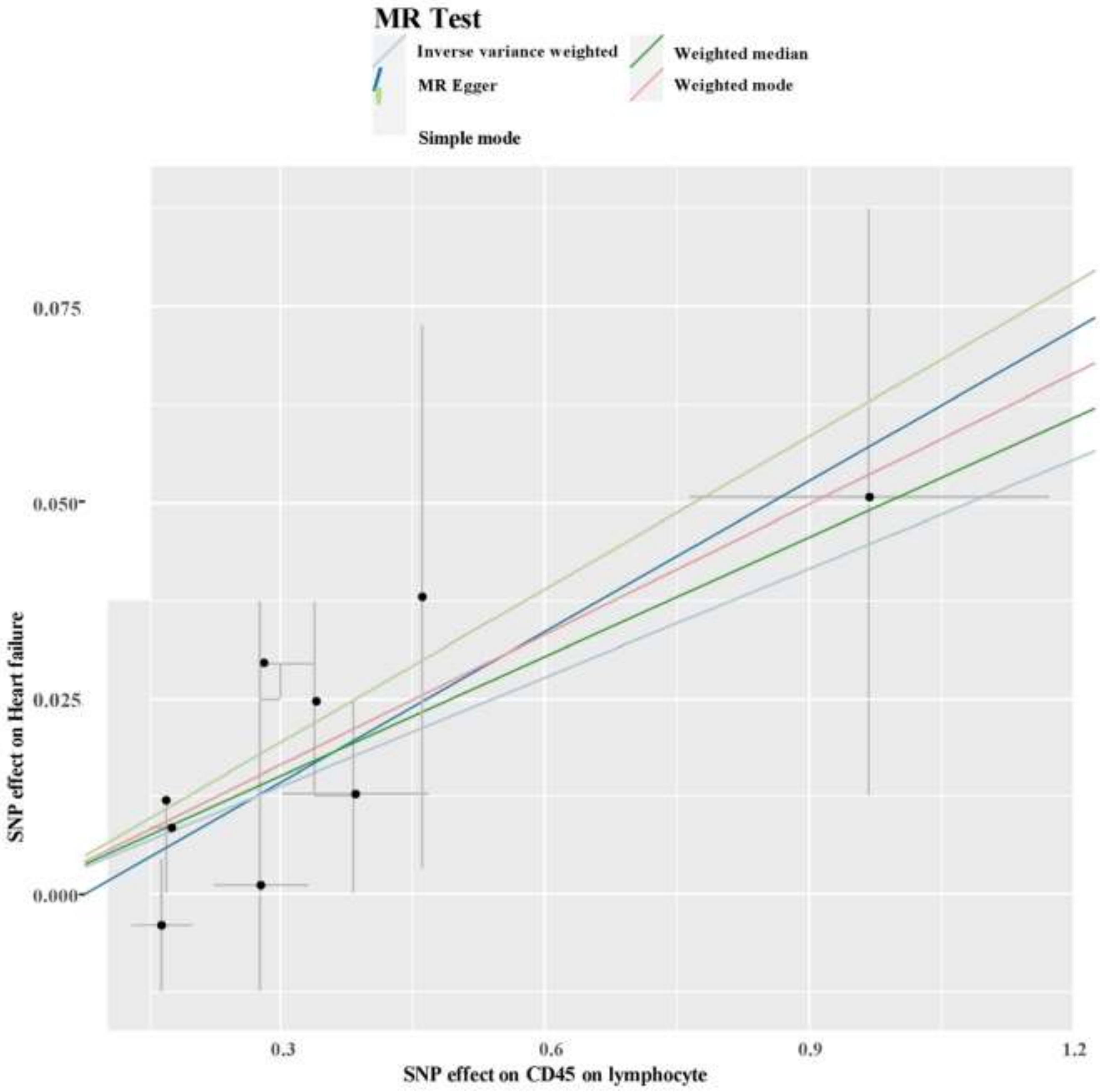

Figure S48: Forest plot of the effect of CD45 on lymphocyte on HF.

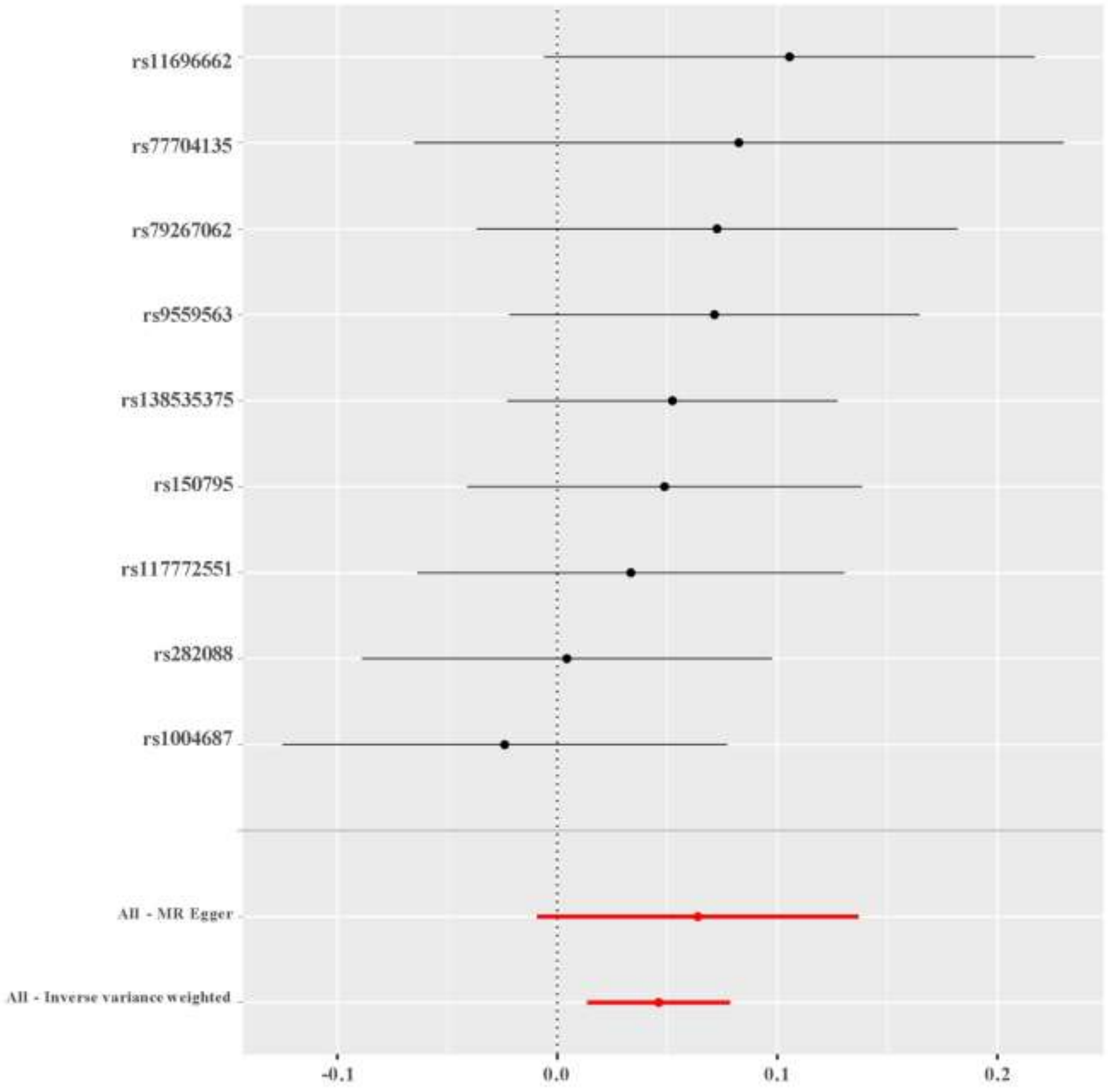

Figure S49: Leave-one-out sensitivity analysis plot of the effect of SSC-A on CD14+ monocyte on HF.

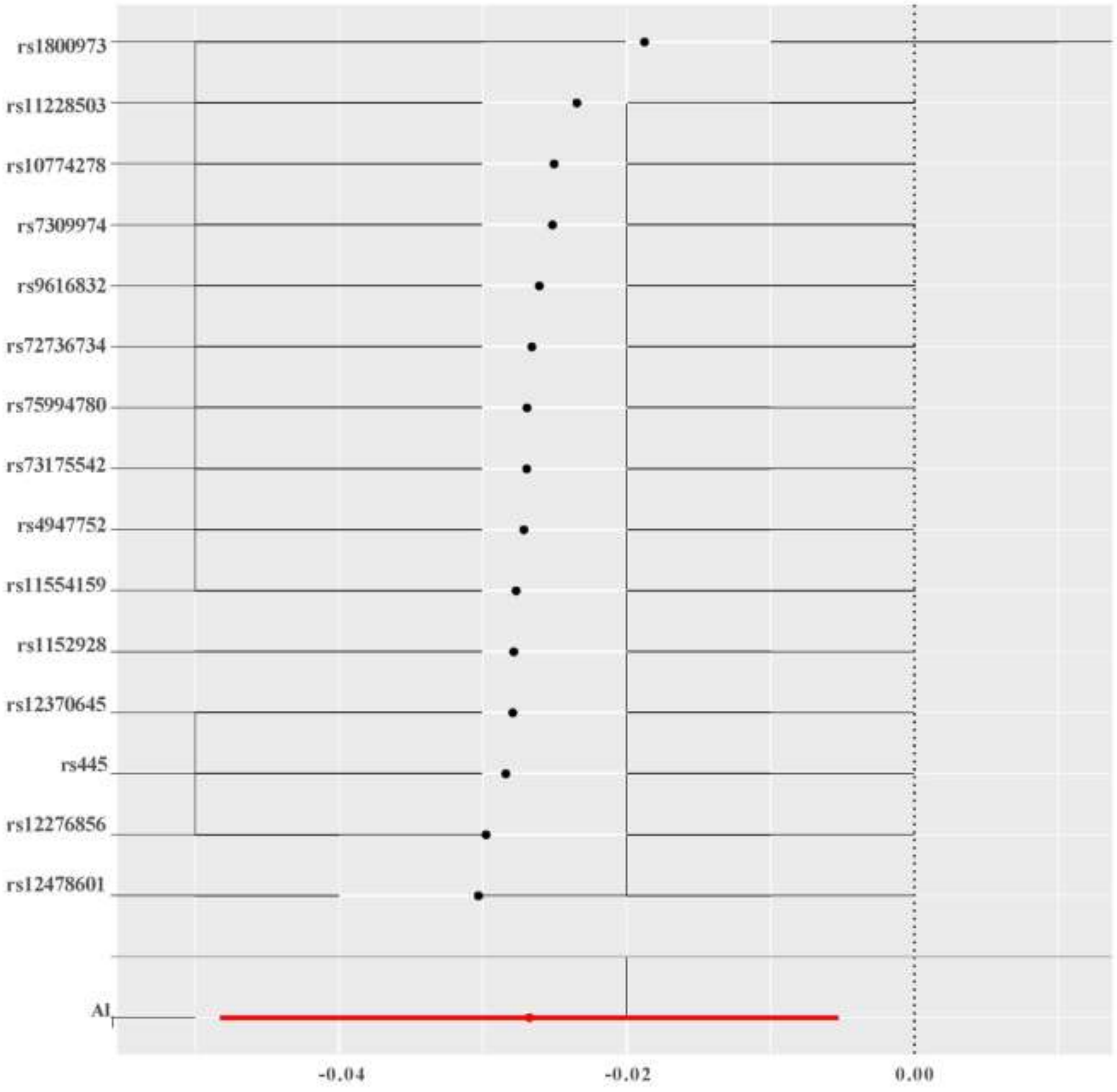

Figure S50: Funnel plot of the effect of SSC-A on CD14$^+$ monocyte on HF.

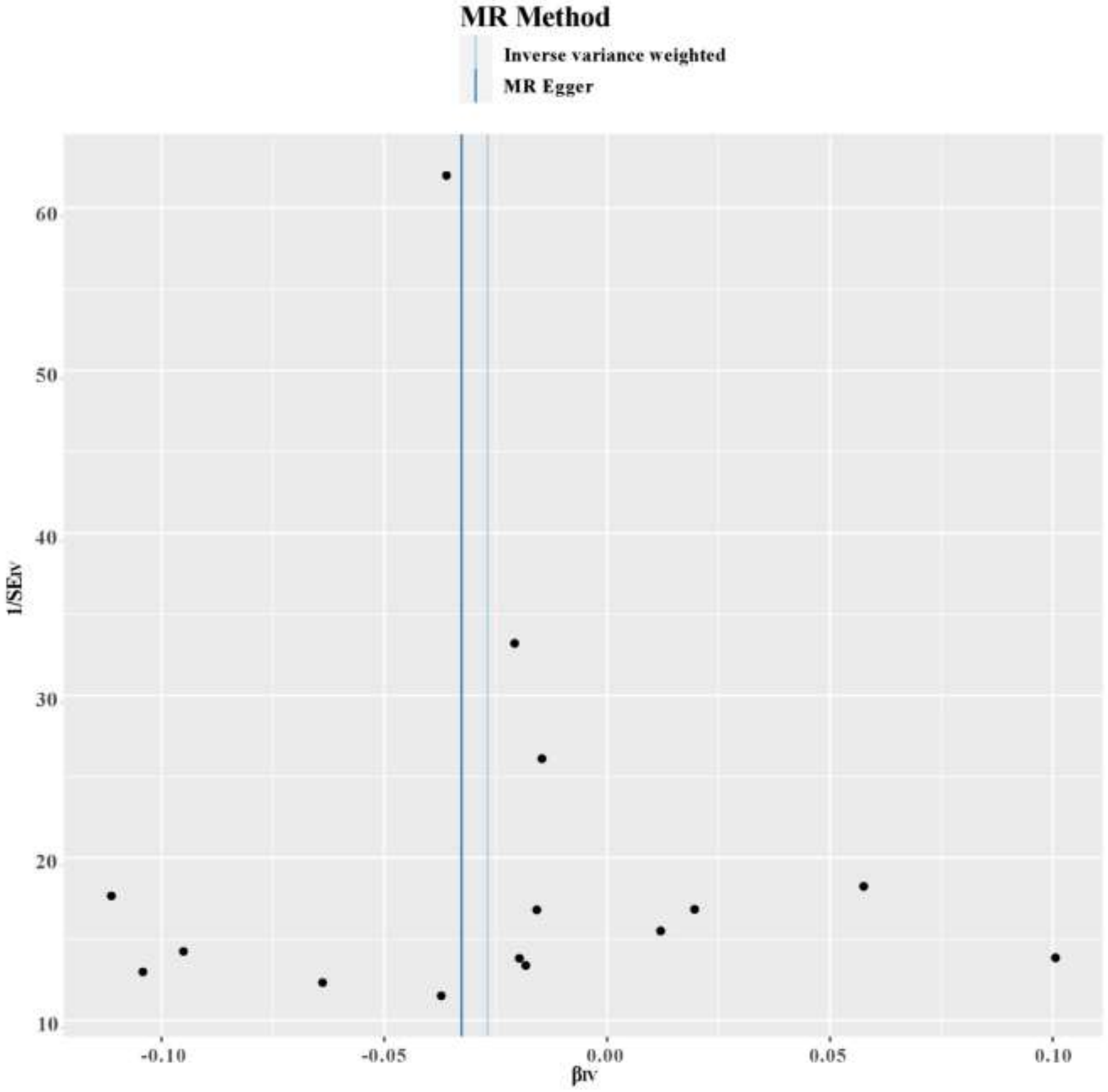

Figure S51: Scatter plot of the effect of SSC-A on CD14⁺ monocyte on HF.

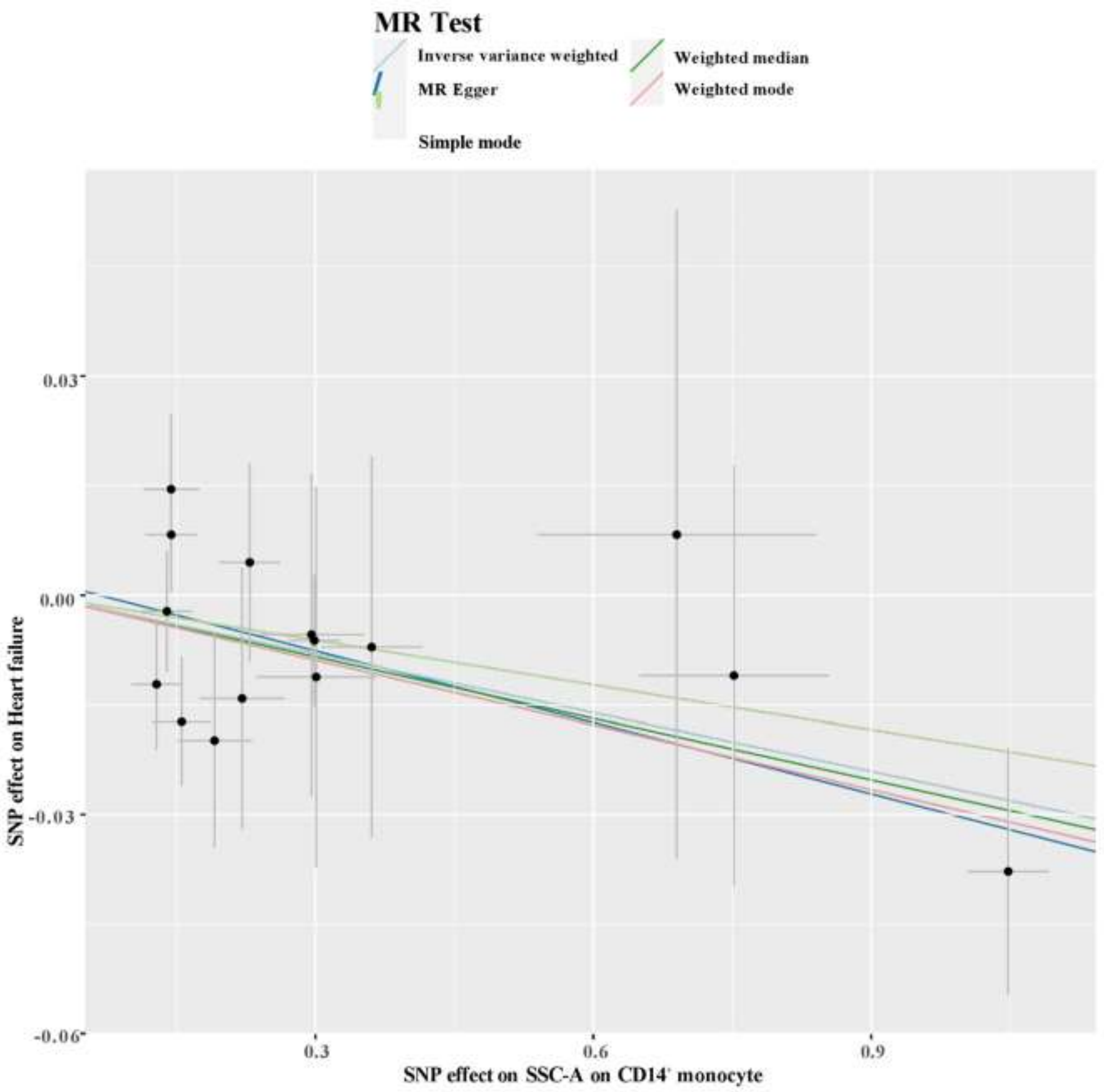

Figure S52: Forest plot of the effect of SSC-A on $CD14^+$ monocyte on HF.

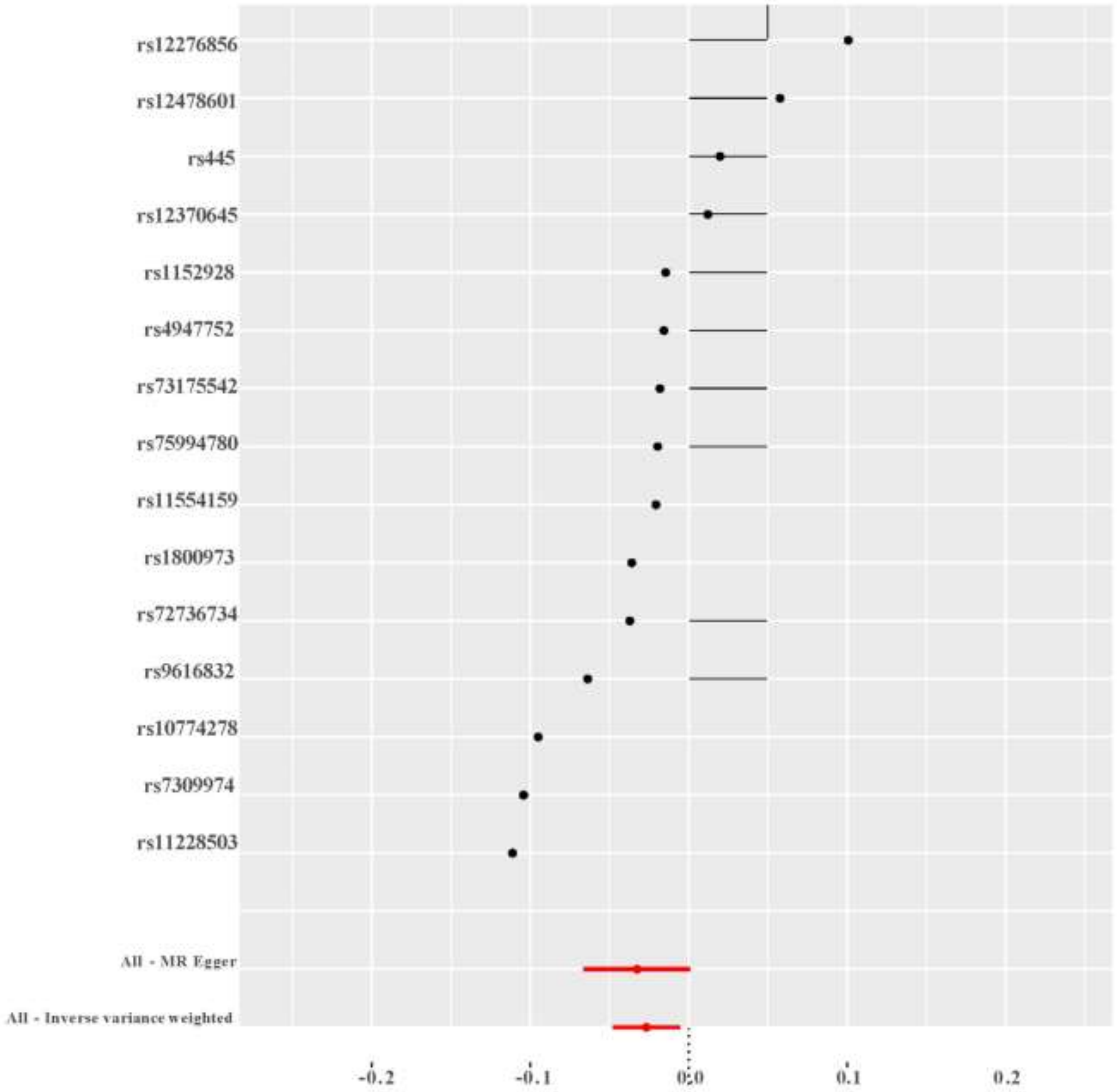

Figure S53: Leave-one-out sensitivity analysis plot of the effect of SSC-A on HLA DR$^+$ NK on HF.

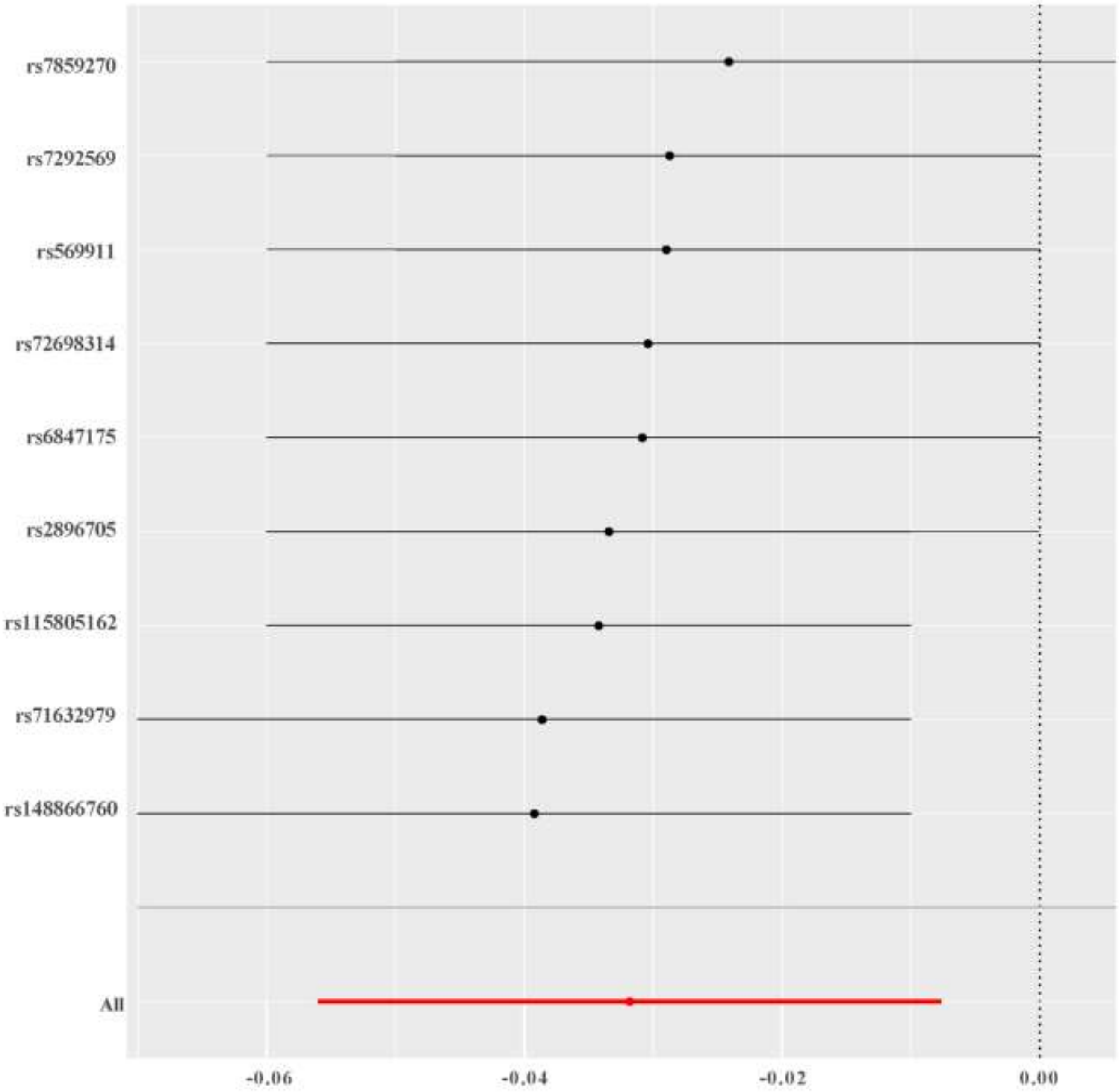

Figure S54: Funnel plot of the effect of SSC-A on HLA DR^+ NK on HF.

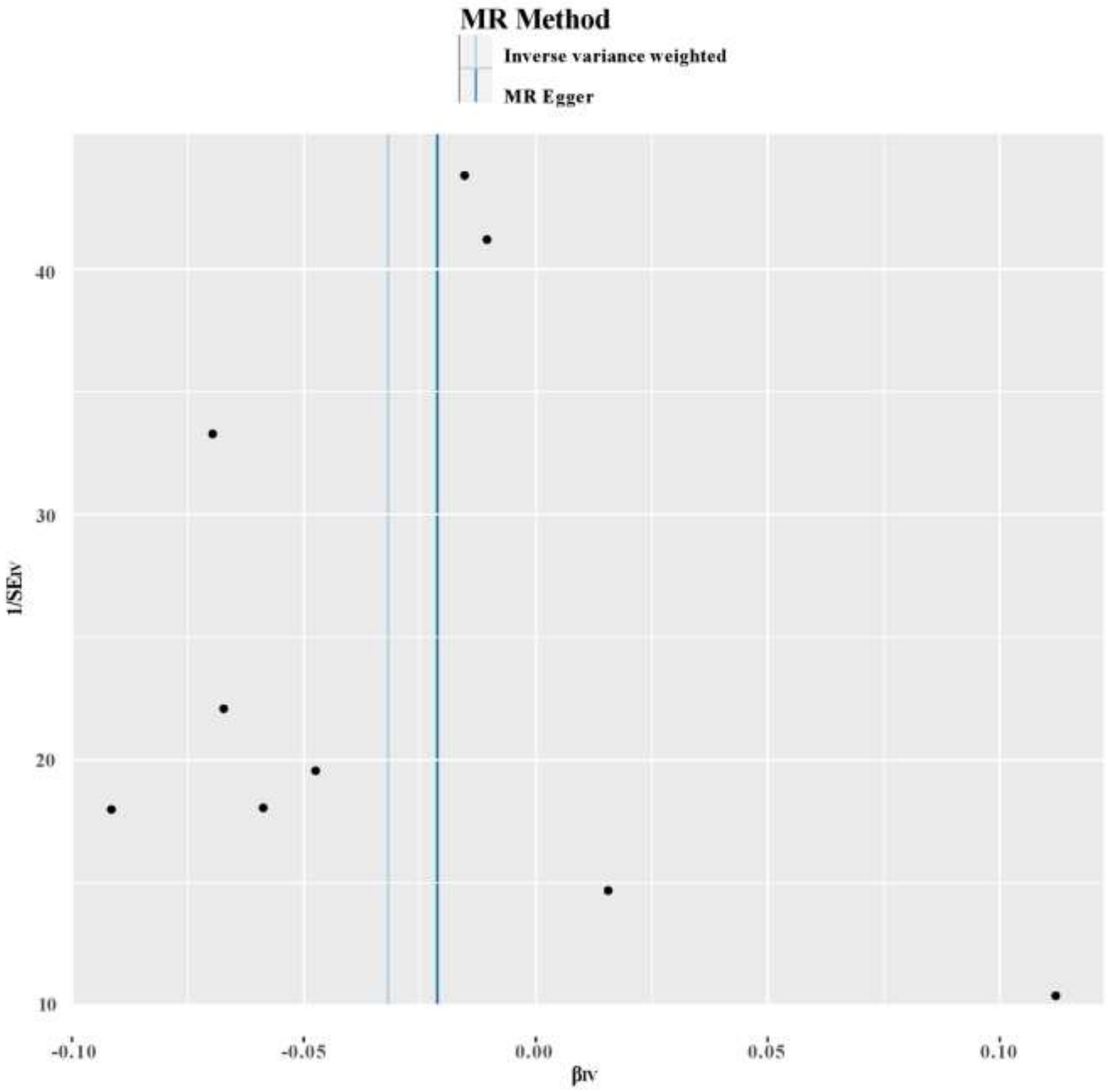

Figure S55: Scatter plot of the effect of SSC-A on HLA DR^+ NK on HF.

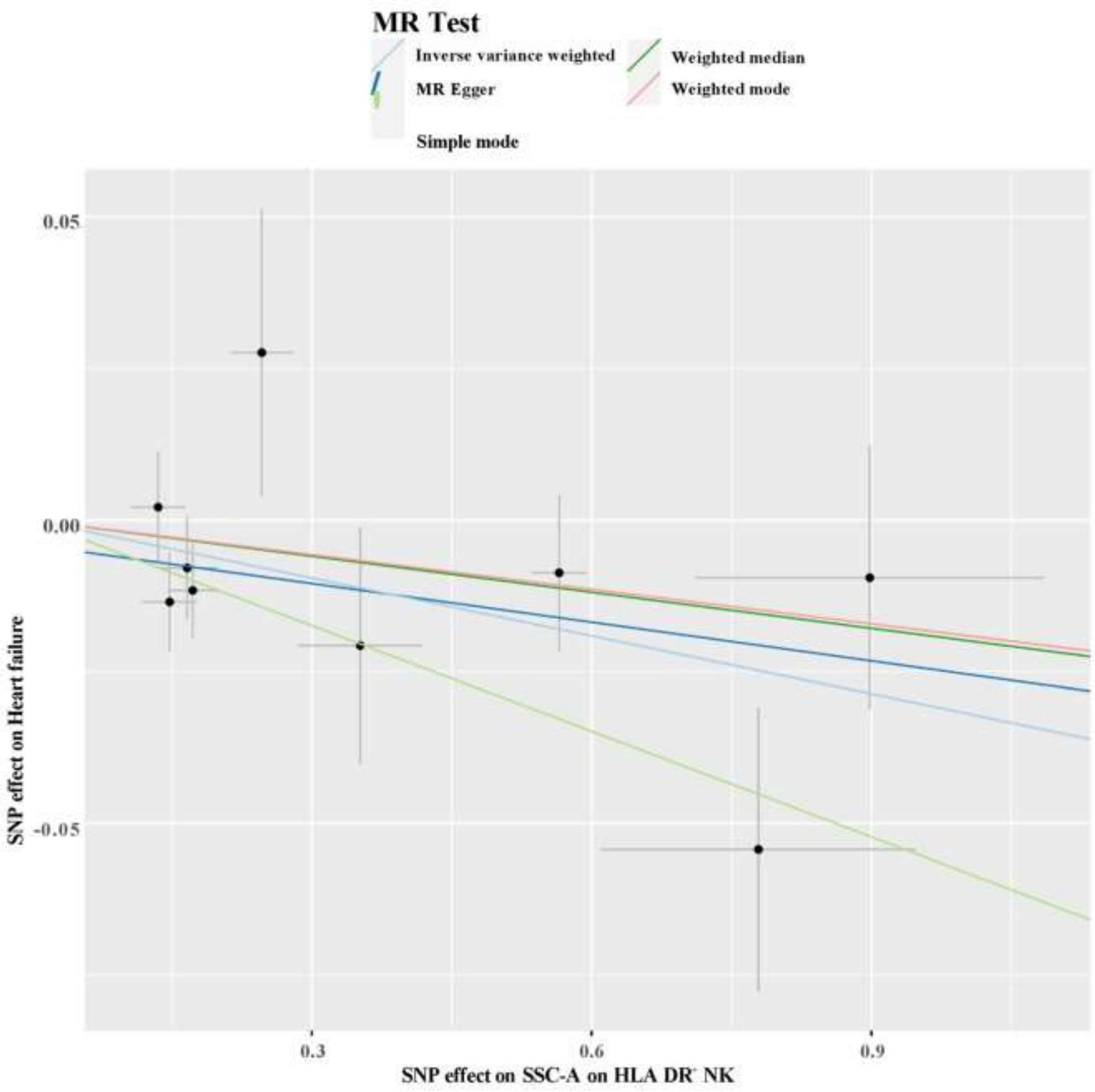

Figure S56: Forest plot of the effect of SSC-A on HLA DR+ NK on HF.

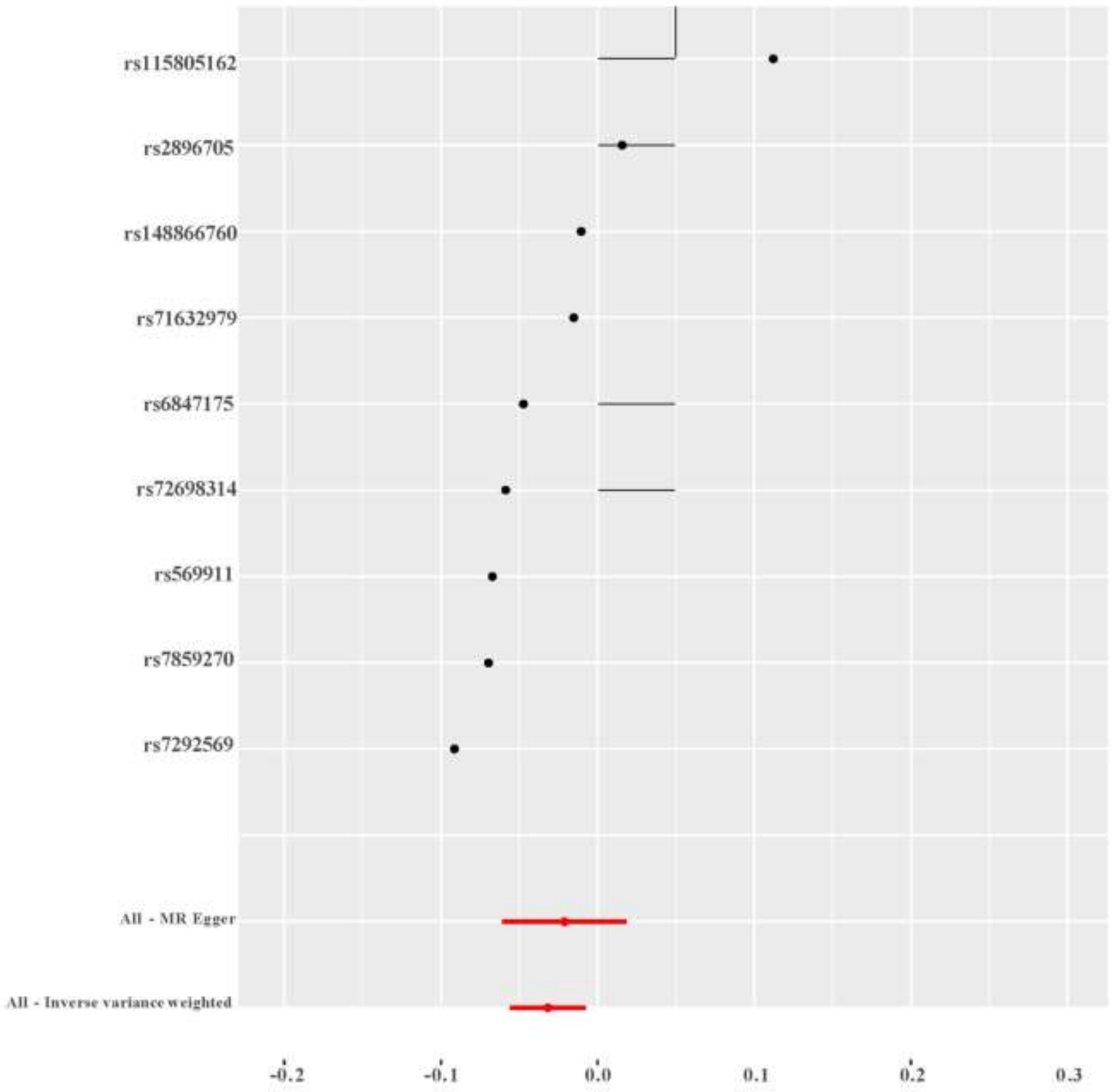

Figure S57: Leave-one-out sensitivity analysis plot of the effect of CD11b on $CD14^+$ monocyte on HF.

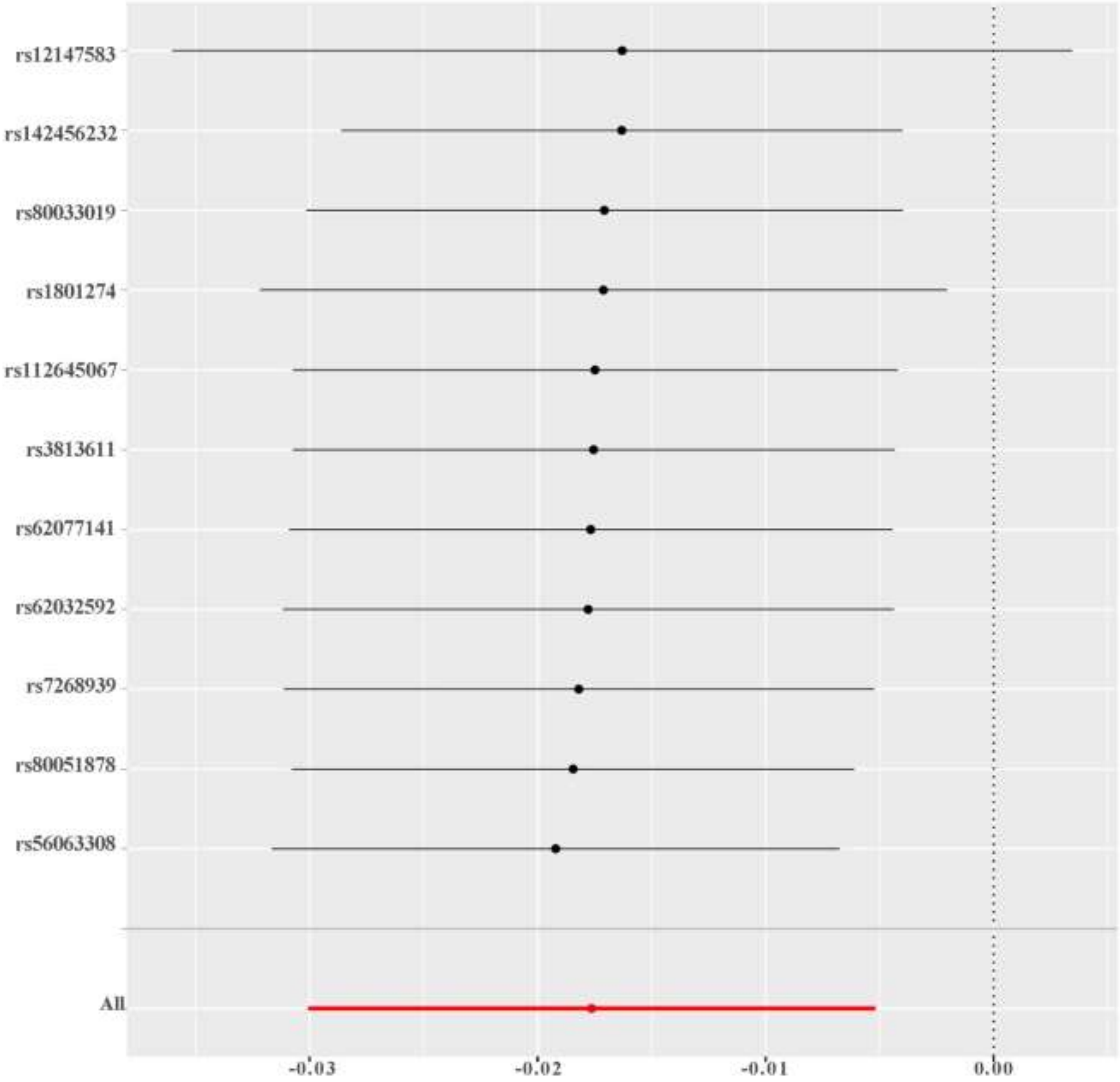

Figure S58: Funnel plot of the effect of CD11b on CD14+ monocyte on HF.

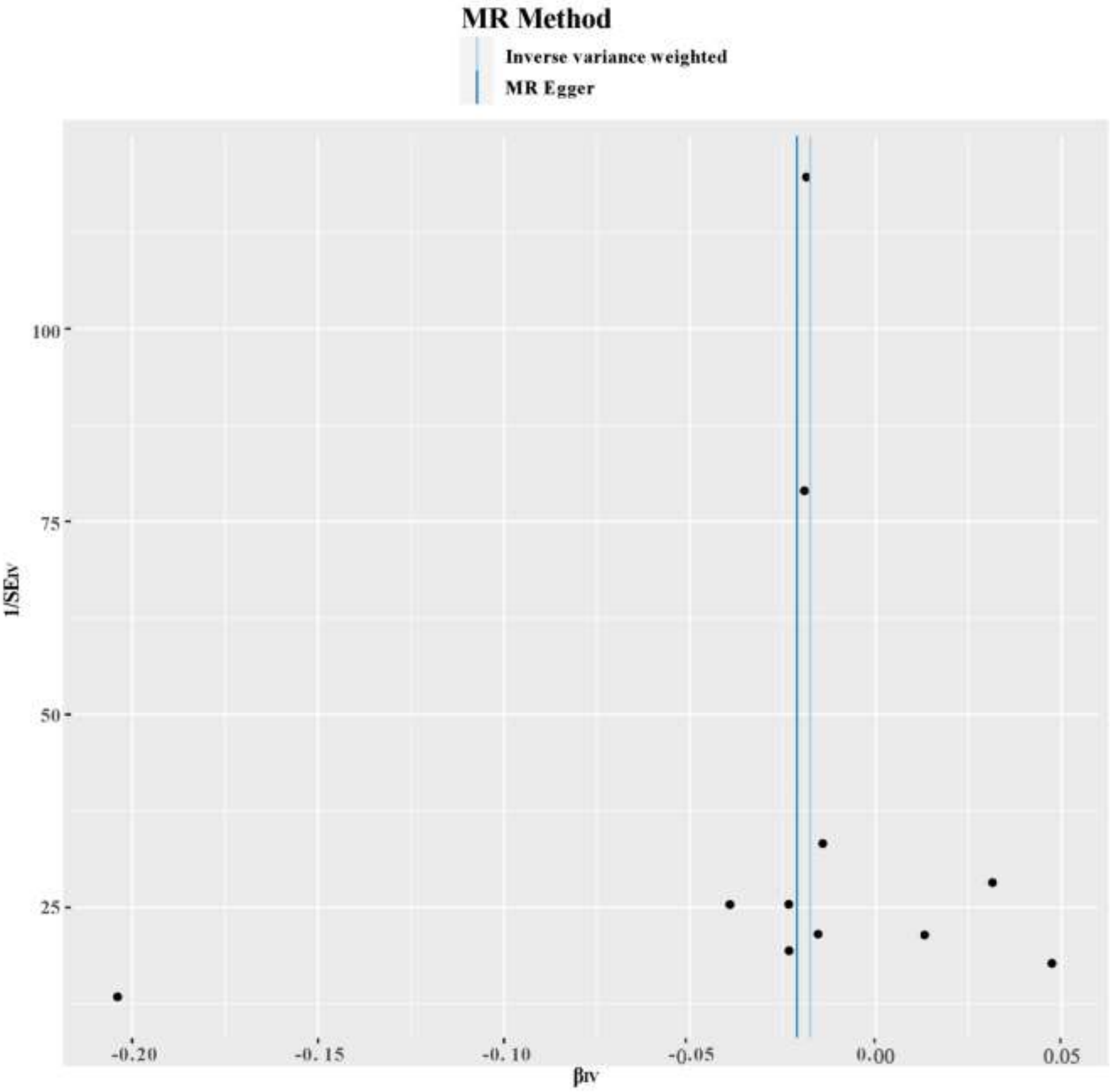

Figure S59: Scatter plot of the effect of CD11b on CD14$^+$ monocyte on HF.

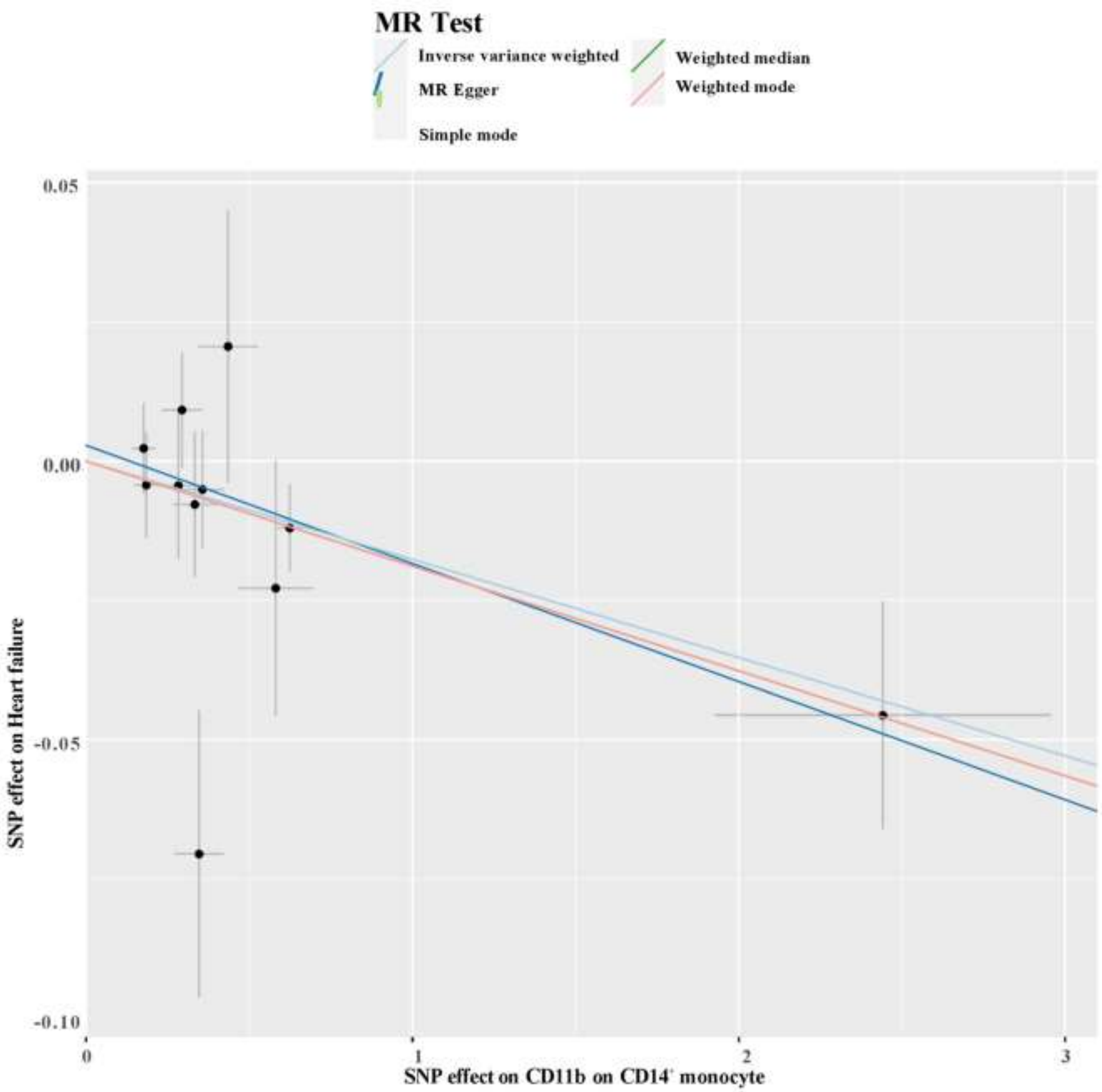

Figure S60: Forest plot of the effect of CD11b on $CD14^{+}$ monocyte on HF.

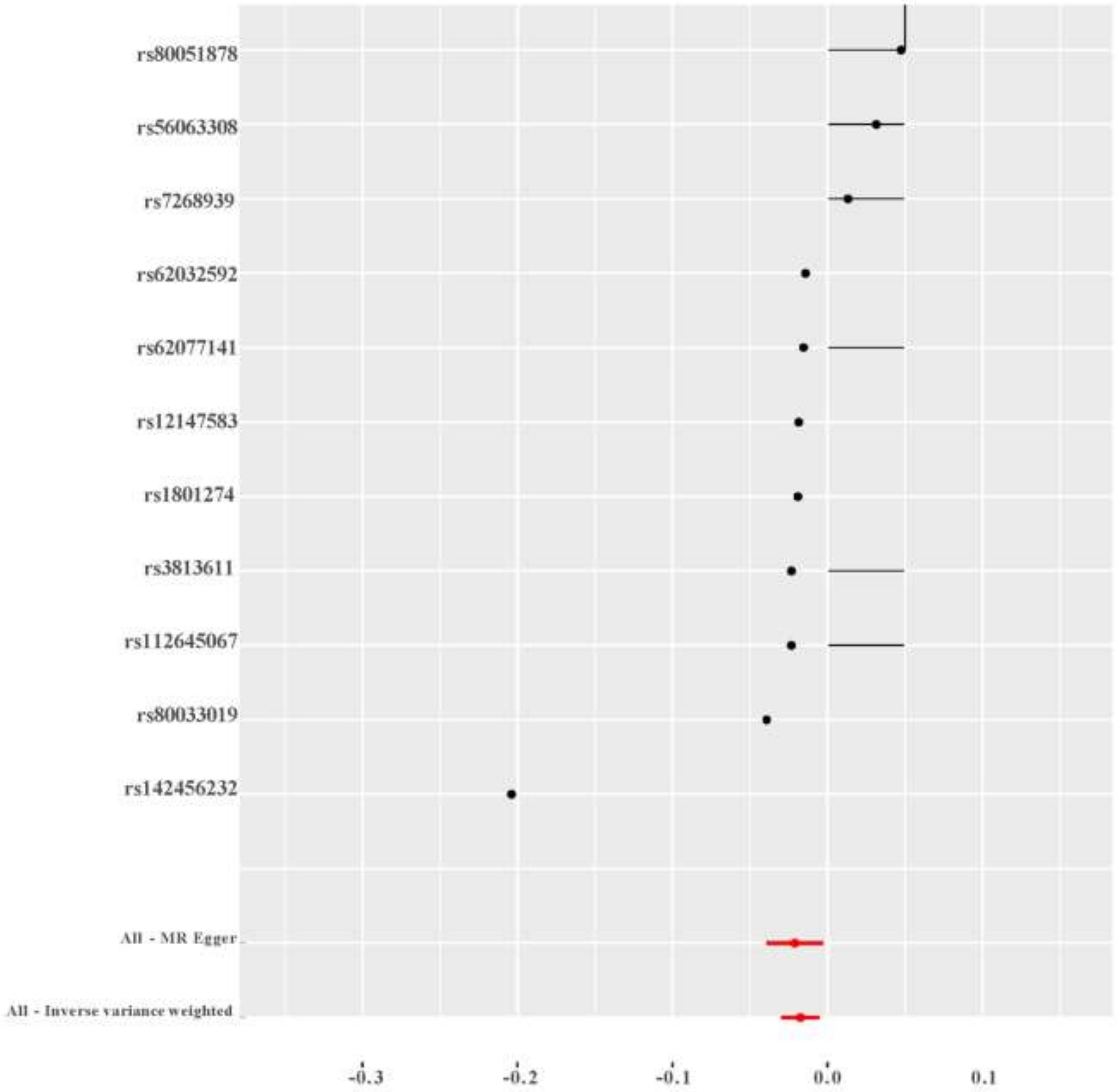

Supplement: Supplementary file 2 [file medi-104-e42530-s002.pdf]
